# Adaptive physiological water conservation explains hypertension and muscle catabolism in experimental chronic renal failure

**DOI:** 10.1111/apha.13629

**Published:** 2021-03-07

**Authors:** Johannes J. Kovarik, Norihiko Morisawa, Johannes Wild, Adriana Marton, Kaoru Takase‐Minegishi, Shintaro Minegishi, Steffen Daub, Jeff M. Sands, Janet D. Klein, James L. Bailey, Jean‐Paul Kovalik, Manfred Rauh, Susanne Karbach, Karl F. Hilgers, Friedrich Luft, Akira Nishiyama, Daisuke Nakano, Kento Kitada, Jens Titze

**Affiliations:** ^1^ Programme in Cardiovascular and Metabolic Disorders Duke‐NUS Medical School Singapore Singapore; ^2^ Clinical Division of Nephrology and Dialysis Department of Internal Medicine III Medical University of Vienna Vienna Austria; ^3^ Department of Pharmacology Faculty of Medicine Kagawa University Kagawa Japan; ^4^ Division for Cardiology 1 Centre for Cardiology Johannes Gutenberg‐University Mainz Mainz Germany; ^5^ Department of Stem Cell and Immune Regulation Yokohama City University Graduate School of Medicine Yokohama Japan; ^6^ Department of Medical Science and Cardiorenal Medicine Yokohama City University Graduate School of Medicine Yokohama Japan; ^7^ Renal Division Department of Medicine Emory University Atlanta GA USA; ^8^ Division of Paediatrics Research Laboratory Erlangen Germany; ^9^ Division of Nephrology and Hypertension University Clinic Erlangen Erlangen Germany; ^10^ Experimental and Clinical Research Center Max Delbrück Center for Molecular Medicine Berlin Germany; ^11^ JSPS Overseas Research Fellow Japan Society for the Promotion of Science Tokyo Japan; ^12^ Division of Nephrology Duke University School of Medicine Durham NC USA

**Keywords:** aestivation, body sodium, body water, dehydration, double‐barrier concept, glucose‐alanine‐shuttle, glycine methylation, hepato‐renal, hypertension, kidney, liver, muscle mass loss, organic osmolytes, purine metabolism, skin, transamination, transepidermal water loss, urea cycle, urine concentration

## Abstract

**Aim:**

We have reported earlier that a high salt intake triggered an *aestivation*‐like natriuretic‐ureotelic body water conservation response that lowered muscle mass and increased blood pressure. Here, we tested the hypothesis that a similar adaptive water conservation response occurs in experimental chronic renal failure.

**Methods:**

In four subsequent experiments in Sprague Dawley rats, we used surgical 5/6 renal mass reduction (5/6 Nx) to induce chronic renal failure. We studied solute and water excretion in 24‐hour metabolic cage experiments, chronic blood pressure by radiotelemetry, chronic metabolic adjustment in liver and skeletal muscle by metabolomics and selected enzyme activity measurements, body Na^+^, K^+^ and water by dry ashing, and acute transepidermal water loss in conjunction with skin blood flow and intra‐arterial blood pressure.

**Results:**

5/6 Nx rats were polyuric, because their kidneys could not sufficiently concentrate the urine. Physiological adaptation to this renal water loss included mobilization of nitrogen and energy from muscle for organic osmolyte production, elevated norepinephrine and copeptin levels with reduced skin blood flow, which by means of compensation reduced their transepidermal water loss. This complex physiologic‐metabolic adjustment across multiple organs allowed the rats to stabilize their body water content despite persisting renal water loss, albeit at the expense of hypertension and catabolic mobilization of muscle protein.

**Conclusion:**

Physiological adaptation to body water loss, termed *aestivation*, is an evolutionary conserved survival strategy and an under‐studied research area in medical physiology, which besides hypertension and muscle mass loss in chronic renal failure may explain many otherwise unexplainable phenomena in medicine.

## 1 INTRODUCTION

Perturbations in salt‐and‐water homeostasis have been implicated in the pathogenesis of arterial hypertension. The intimate relationship between body fluid and blood pressure level is frequently explained by a physiological feedback‐loop model in which an underlying inability of the kidneys to completely excrete surplus dietary salt and water would causally elevate body Na^+^ and fluid content.^1^, ^2^ Expanding on this concept, the pressure‐natriuresis theory suggests that any chronic elevation in blood pressure is aimed to improve the excretion of Na^+^ and water by the kidney, suggesting that hypertension is an adaptive physiological body response that is designed to prevent a chronically increased body Na^+^ and fluid content.^3^, ^4^, ^5^

Studying day‐to‐day changes in blood pressure in relation to 24‐hour solute and water excretion during long‐term balance experiments in humans, we have found that significant spontaneous‐rhythmical fluctuations in body Na^+^ content occurred without parallel changes in blood pressure.^6^ Furthermore, we have reported that increasing salt intake in humans and mice triggered a systemic body water conservation response to overcome the osmotic‐diuretic effect of increased urinary salt excretion, which shows surprising similarities to *aestivation* metabolism in amphibians in fish during physiological adaptation to water shortage.^7^, ^8^ Because these findings are antipodal to current textbook teaching on the pathogenesis of arterial hypertension, we tested our alternative view on the relationship between salt and water homeostasis and blood pressure in rats with chronic renal failure. We hypothesized that an inability to concentrate the urine would induce these evolutionary‐conserved extrarenal water conservation patterns in rats with experimental renal mass reduction.

We show that chronic renal failure leads to renal water loss, which triggers an extrarenal compensatory body response to successfully prevent dehydration. Understanding that the animals activate an *aestivation*‐like systemic metabolic and circulatory body response designed to prevent dehydration not only explains their “renal” hypertension, but also why muscle mass loss occurs in chronic renal failure.

## 2 RESULTS

### 2.1 Experimental renal mass reduction leads to renal water loss

In a 24‐hour metabolic cage experiment, we first tested the hypothesis that 5/6 reduction of renal mass (5/6 Nx) in rats led to reduced renal solute or water excretion (Table 1). We found that neither food intake, which represents solute intake, nor urine solute excretion, was different between control and 5/6 Nx rats. We interpret this finding to show intact solute excretion in 5/6 Nx rats, despite significant renal mass reduction.

**TABLE 1:** 24‐h urine solute excretion, urine solute concentration, plasma solute concentration and water balance in control rats and in rats with 5/6 renal mass reduction (5/6 Nx)

	Control (n = 6)	5/6 Nx (n = 12)	*P* value
*24‐h Food Intake and Solute Excretion*			
Food Intake (g/kg/d)	50.1 ± 4.8	54.0 ± 7.0	.243
UNaV (mmol/kg/d)	3.7 ± 0.4	3.6 ± 1.0	.748
UKV (mmol/kg/d)	8.8 ± 1.2	9.2 ± 1.4	.584
UUreaV (mmol/kg/d)	40.9 ± 4.9	40.0 ± 11.8	.867
*24‐h Water Intake, Urine Volume and Water Balance Gap*			
Water Intake (mL/kg/d)	67.6 ± 2.9	120.1 ± 20.4	<.001
Urine Volume (mL/kg/d)	30.2 ± 3.8	60.7 ± 15.1	<.001
Water Balance Gap (mL/kg/d)	37.0 ± 3.1	60.0 ± 12.6	<.01
Δ Body Weight (g)	−4.7 ± 2.9	−7.3 ± 9.5	.529
*24‐h Urine Solute Concentration and Free Water Clearance*			
Urine [Na^+^] (mmol/L)	124.7 ± 23.4	59.3 ± 12.2	<.001
Urine [K^+^] (mmol/L)	292.7 ± 29.2	156.9 ± 25.8	<.001
Urine [Urea] (mmol/L)	1372 ± 234	677 ± 184	<.001
Urine Osmolality (mOsm/kg)	2004 ± 249	1009 ± 143	<.001
Free Water Clearance (mL/kg/d)	−188.4 ± 23.3	−154.5 ± 30.9	<.05
*Plasma Solute Concentrations and Plasma Osmolality*			
Plasma [Na^+^] (mmol/L)	138.9 ± 1.8	138.8 ± 2.0	.912
Plasma [K^+^] (mmol/L)	6.6 ± 0.5	7.3 ± 0.7	<.05
Plasma [Urea] (mmol/L)	4.1 ± 0.8	9.7 ± 1.6	<.001
Plasma Osmolality (mOsm/kg)	282 ± 7	289 ± 4	<.05

Note

Data are expressed as average ± SD.

**FIGURE 1:**
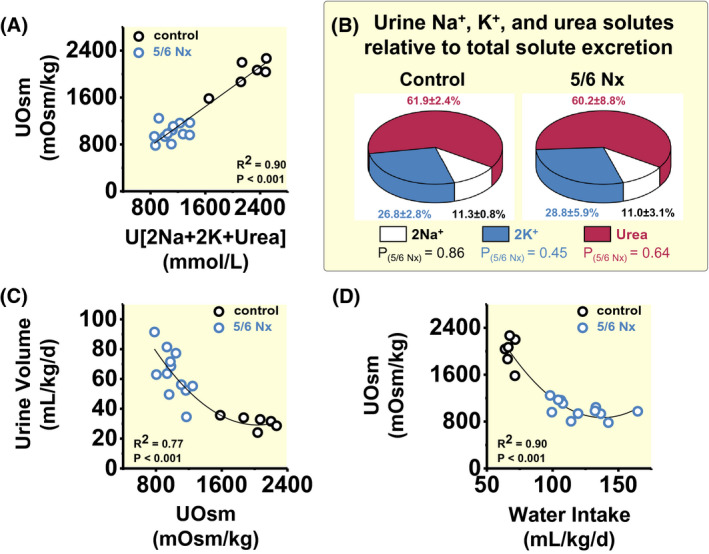
Chronic renal failure leads to renal water loss. A, Contribution of 24‐h urine Na^+^, K^+^ and urea solute concentration to urine osmolality (UOsm) in control rats (n = 6) and in rats with 5/6 renal mass reduction (5/6 Nx; n = 12). Note that Na^+^ and K^+^ excretion are calculated twofold to account for the excretion of unmeasured accompanying anions. B, Relative contribution of Na^+^, K^+^ and urea solutes to urine solute formation in the same rats. Data expressed as average ± SD. C, Relationship between urine osmolality and urine volume in the same rats. D, Relationship between water intake and urine osmolality in the same rats

We next studied urine concentration in 5/6 Nx rats. Renal mass ablation resulted in a 50% reduction in urine osmolality, and increased the urine free‐water excretion by 33.9 mL/kg/d, which corresponded well to the observed 30.5 mL/kg/d increase in urine volume formation (Table 1). The change in the rats’ urine osmolality was almost entirely explained by the reduction of the Na^+^, K^+^, urea, and accompanying anion concentrations in the urine (Figure 1A). The major urine solute was urea (Figure 1B), followed by K^+^ (with accompanying anions) and Na^+^ (with accompanying anions). Renal mass reduction did not change the relative contribution of urea, K^+^ or Na^+^ solutes to solute excretion or solute concentration in the urine.

Compared to their healthy controls, 5/6 Nx rats increased their urine volume by 101%, and their fluid intake by 78% (Table 1). We hypothesized that renal mass reduction led to a urine concentration deficit with polyuria, which triggered increased fluid intake to prevent dehydration (Figure 1C, primary polyuria). The alternative hypothesis, however, was that 5/6 Nx rats might have primarily increased their fluid intake, and therefore had subsequently increased their urine volume (Figure 1D, primary polydipsia). We therefore next analysed their blood plasma and found that, despite increased fluid intake, 5/6 Nx led to an increase in plasma osmolality (Table 1), indicating a body water conservation response to prevent dehydration. Approximately 79% of this water conservation response was explained by an increase in plasma urea concentration, and another 20% of the increase in plasma osmolality was explained by elevated concentration of the plasma K^+^ solutes and their accompanying anions (Table 1). Plasma Na^+^ solutes with accompanying anions did not contribute to the measured change in plasma osmolality (Table 1).

We interpret these findings to show that renal‐mass ablation caused an inability to concentrate the urine, and that the resulting polyuria predisposed the rats to body water loss.

### 2.2 Experimental renal mass reduction leads to arterial hypertension, and to tissue solute and water accumulation

Chronic renal failure is believed to reduce renal solute and water excretion, resulting in body solute and water accumulation, and thereby increasing blood pressure. We therefore studied the relationship between blood pressure, urine solute and water excretion, and tissue Na^+^, K^+^ and water content in our rats.

**FIGURE 2:**
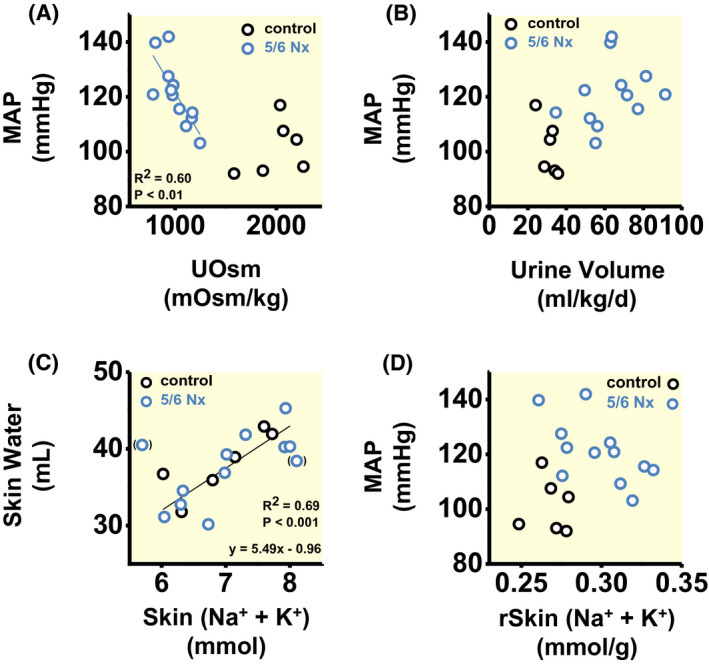
Relationship between renal water loss, tissue water content, and blood pressure. A, Relationship between urine osmolality and mean arterial blood pressure (MAP). B, Relationship between urine volume and blood pressure. C, Relationship between absolute Na^+^+ K^+^ and absolute water content in the skin. ( ): we excluded the data points with lowest and with highest Na^+^+ K^+^ content from regression analysis. D, Relationship between skin Na^+^+ K^+^ content per gram dry skin and mean arterial blood pressure. Data are from the same rats shown in Figure 1 and Tables 1 and 2

Compared to healthy controls, renal mass ablation increased mean arterial blood pressure (101 ± 10 vs 121 ± 12 mmHg; *P* < .01). Within the 5/6 Nx animal group, blood pressure steeply increased with reduced urine concentration (Figure 2A). Their arterial hypertension therefore occurred with increased urine volume (Figure 2B); however, we could not find a similarly significant relationship between urine volume and blood pressure solely within the 5/6 Nx group.

We next studied changes in body solute and water content in our rats by chemical analysis (Table 2). Except for a small reduction in absolute Na^+^ content in the skinned carcass, absolute Na^+^, K^+^ and water content in rats with renal mass reduction was not different from their healthy controls, neither at the total body level, nor in their skin or in the skinned carcass. However, 5/6 Nx rats showed reduced tissue dry weight, indicating catabolism with stunted growth. 5/6 Nx rats therefore increased their tissue Na^+^, K^+^, and water content per tissue mass (Figure S1). Analysis of the tissue solute‐to‐water ratio showed total body and tissue [Na^+^ + K^+^] concentrations that were consistently higher than their plasma [Na^+^ + K^+^] concentrations. This concentration difference persisted in skin tissue, which is bone‐ and cartilage‐free. In the skin, an increase in Na^+^ + K^+^ solutes of 2 mmol was paralleled by an 11 mL increase in water content, resulting in a skin tissue [Na^+^ + K^+^] solute concentration of ≈181 mmol/L (Figure 2C). The resulting accumulation of Na^+^ and K^+^ per skin tissue mass in 5/6 Nx rats (Table 2), which was paralleled by less fluid accumulation than predicted by the theory of isotonic body fluid distribution (Table 2 and Figure 2C), was associated with increased blood pressure (Figure 2D). We found no direct or indirect correlation between skin tissue Na^+^ and K^+^ overload and blood pressure in the 5/6 Nx animal group alone (Figure 2D).

**TABLE 2:** Chemical analysis of Na^+^, K^+^ and water content in total body, skin and skinned rest carcass from the same rats as reported in Table 1

	Control (n = 6)	5/6 Nx (n = 12)	*P* value
*Total body (TB) Na^+^, K^+^ and water content and concentrations*			
TB Wet Weight (g)	274.2 ± 11.1	264.7 ± 21.3	.326
TB Dry Weight (g)	92.6 ± 4.6	85.4 ± 6.9	<.05
TB Na^+^ Content (mmol)	14.59 ± 0.61	14.12 ± 0.77	.212
rTB Na^+^ Content (mmol/g DW)	0.158 ± 0.007	0.166 ± 0.006	<.05
TB K^+^ Content (mmol)	23.47 ± 0.66	22.92 ± 1.69	.454
rTB K^+^ Content (mmol/g DW)	0.254 ± 0.011	0.269 ± 0.014	<.05
TB Water Content (mL)	181.6 ± 6.7	179.3 ± 15.0	.730
rTB Water Content (mL/g DW)	1.963 ± 0.036	2.102 ± 0.086	<.01
TB Na^+^ / Water Ratio (mmol/mL)	0.080 ± 0.003	0.079 ± 0.004	.432
TB K^+^ / Water Ratio (mmol/mL)	0.129 ± 0.005	0.128 ± 0.008	.732
TB (Na^+^ + K^+^) / Water Ratio (mmol/mL)	0.210 ± 0.007	0.207 ± 0.010	.576
*Skinned rest carcass (Car) Na^+^, K^+^ and water content and concentrations*			
Car Wet Weight (g)	210.2 ± 9.2	203.4 ± 14.9	.323
Car Dry Weight (g)	66.7 ± 3.1	61.7 ± 4.2	<.05
Car Na^+^ Content (mmol)	10.55 ± 0.29	10.08 ± 0.41	<.05
rCar Na^+^ Content (mmol/g DW)	0.159 ± 0.007	0.164 ± 0.008	.186
Car K^+^ Content (mmol)	20.58 ± 0.65	19.93 ± 1.54	.342
rCar K^+^ Content (mmol/g DW)	0.309 ± 0.019	0.323 ± 0.019	.153
Car Water Content (mL)	143.6 ± 6.5	141.7 ± 11.1	.712
rCar Water Content (mL/g DW)	2.154 ± 0.055	2.296 ± 0.087	<.01
Car Na^+^ / Water Ratio (mmol/mL)	0.074 ± 0.003	0.071 ± 0.004	.268
Car K^+^ / Water Ratio (mmol/mL)	0.144 ± 0.007	0.141 ± 0.010	.574
Car (Na^+^ + K^+^) / Water Ratio (mmol/mL)	0.217 ± 0.009	0.212 ± 0.012	.408
*Skin (SK) Na^+^, K^+^, and water content and concentrations*			
SK Wet Weight (g)	63.9 ± 6.6	61.3 ± 7.1	0.453
SK Dry Weight (g)	25.9 ± 2.8	23.6 ± 2.9	0.136
SK Na^+^ Content (mmol)	4.09 ± 0.44	4.04 ± 0.50	0.986
rSK Na^+^ Content (mmol/g DW)	0.156 ± 0.007	0.171 ± 0.011	<0.01
SK K^+^ Content (mmol)	2.89 ± 0.31	2.99 ± 0.37	0.609
rSK K^+^ Content (mmol/g DW)	0.112 ± 0.010	0.127 ± 0.013	<0.05
SK Water Content (mL/g DW)	38.0 ± 4.1	37.6 ± 4.6	0.852
rSK Water Content (mL/g DW)	1.471 ± 0.100	1.595 ± 0.134	0.061
SK Na^+^ / Water Ratio (mmol/mL)	0.106 ± 0.006	0.108 ± 0.010	0.708
SK K^+^ / Water Ratio (mmol/mL)	0.077 ± 0.008	0.080 ± 0.012	0.497
SK (Na^+^ + K^+^) / Water Ratio (mmol/mL)	0.183 ± 0.012	0.188 ± 0.021	0.567

Note

Data are expressed as average ± SD. Total body parameters are the sum of rest carcass and skin parameters.

Abbreviations: DW, dry weight; r, solute and water content normalized to dry tissue mass.

We interpret these findings to show that the arterial hypertension that occurred in 5/6 Nx rats, together with increased fluid intake and tissue solute accumulation, was likely part of a systemic body response that was designed to prevent dehydration. We hypothesized that physiological adaptation to water shortage, termed *aestivation*, might stabilize body water content during states of chronic renal water loss. We therefore next tested the hypothesis that the renal water loss had triggered a systemic extrarenal metabolic *aestivation‐like* water conservation response in 5/6 Nx rats.

### 2.3 Extrarenal water conservation motif 1: urea cycle activation and exploitation of energy and nitrogen stored in skeletal muscle

The increase in plasma urea concentration in 5/6 Nx rats, which occurred without a parallel increase in plasma Na^+^ concentration and in the absence of reduced renal urea excretion (Table 1), suggests that renal fluid loss in chronic renal failure may have triggered a compensatory hepatic *aestivation* response. To test this hypothesis, we conducted a second independent experiment, in which we first confirmed the water‐losing renal phenotype in 5/6 Nx rats (Figure S2), and then studied the nitrogen‐dependent metabolic pathways of organic osmolyte production.

#### 2.3.1 Urea cycle activation

**FIGURE 3:**
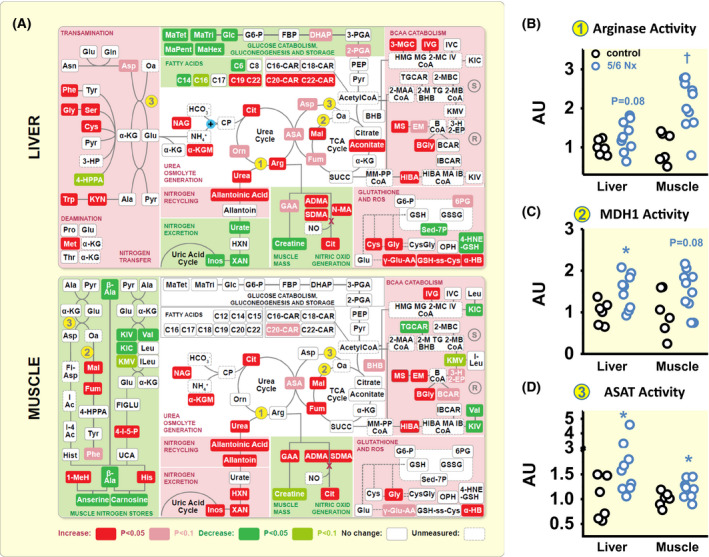
Chronic renal failure leads to reprioritization of liver and muscle nitrogen metabolism for water conservation. A, Metabolomic analysis of differences in nitrogen utilization for urea osmolyte production, urate production, creatine and NO production and nitrogen transfer from the muscle branched‐chain amino acids (BCAA) and muscle dipeptides, carnosine and anserine, between control (n = 6) and 5/6 Nx (n = 10) rats. Key enzymes for urea generation are arginase (#1), cytoplasmatic malate dehydrogenase 1 (MDH1; #2), and aspartate aminotransferase (ASAT; #3). B, Arginase activity in control (n = 6) and 5/6 Nx rats (n = 10). C, Malate dehydrogenase 1 (MDH1) activity in the same rats. D, Aspartate aminotransferase (ASAT) activity in the same rats. *P < .05; †*P* < .01. For abbreviations of the metabolites, see Table S1

Urea production in the liver requires nitrogen transfer from amino acids to glutamate, and nitrogen transfer as NH_4_^+^ for carbamoyl phosphate (CP) synthesis (Figure 3A). We found increased hepatic amino acid levels, increased α‐ketoglutaramate levels (α‐KGM), which is an indicator of hepatic NH_4_^+^ overload^9^; increased N‐acetyl‐glutamate levels (NAG), which is the allosteric effector that activates CP enzyme activity^10^; elevated arginase enzyme activity (the rate‐limiting enzyme for nitrogen transfer from arginine into urea; Figure 3B), and increased hepatic urea content in 5/6 Nx rats. We interpret these findings to show that renal water loss in rats with chronic renal failure triggered the *aestivation* motif of increased urea osmolyte production and accumulation, in an effort to stabilize body water content.

#### 2.3.2 Origin of the nitrogen used for urea synthesis

Because we found no evidence of increased food intake in 5/6 Nx rats and thus concluded that protein intake was similar between the groups (Table 1), we next tested the hypothesis that the urea cycle activation was coupled with endogenous protein exploitation. Besides CP synthesis from HCO_3_^‐^ and NH_4_^+^, nitrogen transfer into the urea cycle relies on the generation of the carbonic acid oxaloacetate (Oa) from malate (Mal), catalysed by the cytoplasmatic form of the enzyme malate dehydrogenase (MDH1; Figure 3A,C), followed by transamination of oxaloacetate to aspartate. This transamination step is catalysed by the cytoplasmatic enzyme aspartate aminotransferase (ASAT; Figure 3A,D). We found that 5/6 Nx rats increased transfer of nitrogen into the urea cycle by promoting cytoplasmatic oxaloacetate generation via increased MDH1 activity (Figure 3C), and by promoting carbonic acid transamination via increased ASAT activity (Figure 3D).

The carbonic acids α‐keto‐β‐methylvalerate (KMV), α‐keto‐isocaproate (KIC), and α‐keto‐isovalerate (KIV) are generated by the catabolism of the branched chain amino acids (BCAA) isoleucine (ILeu), leucine (Leu) and valine (Val; Figure 3A). The catabolism of the BCAAs^11^ is initiated by transfer of their nitrogen group to the carbonic acids pyruvate (Pyr) to form alanine (Ala), oxaloacetate (Ox) to form aspartate (Ast), or α‐ketoglutarate (α‐KG) to form glutamate (Glu) by transamination (Figure 3A). Rats with experimental renal mass reduction showed decreased valine, KMV, KIC and KIV levels in conjunction with elevated intermediates of BCAA catabolism, including their ketogenic intermediate, β‐hydroxybutyrate (BHB; Figure 3A). To better understand the origin of increased BHB in muscle, we in parallel examined ketone production in the liver. We found no signs of increased hepatic ketone body production in 5/6 Nx rats (Figure 3A), indicating that the increased β‐hydroxybutyrate levels in skeletal muscle most likely resulted from exploitation of muscle protein with BCAA catabolism.

Anserine and carnosine are dipeptides that contain 1‐methylhistidine (1‐MeH) or histidine (His) together with β‐alanine (β‐Ala), which is not integrated into the muscle protein matrix. The two dipeptides can therefore be viewed as dedicated metabolic muscle energy and nitrogen reservoirs that can be rapidly mobilized without reducing muscle fibre mass.^12^ We found reduced levels of both peptides in the skeletal muscle of 5/6 Nx rats. The exploitation of the anserine and carnosine muscle reservoir was paralleled by increased histidine and 1‐methylhistidine levels.

We interpret these findings to show that 5/6 Nx rats with renal water loss exploited their muscle BCAAs as well as their anserine and carnosine stores to support hepatic urea osmolyte production in an effort to stabilize their body water content.

### 2.4 Extrarenal water conservation motif 2: experimental renal mass reduction promotes organic osmolyte production by glycine methylation

**FIGURE 4:**
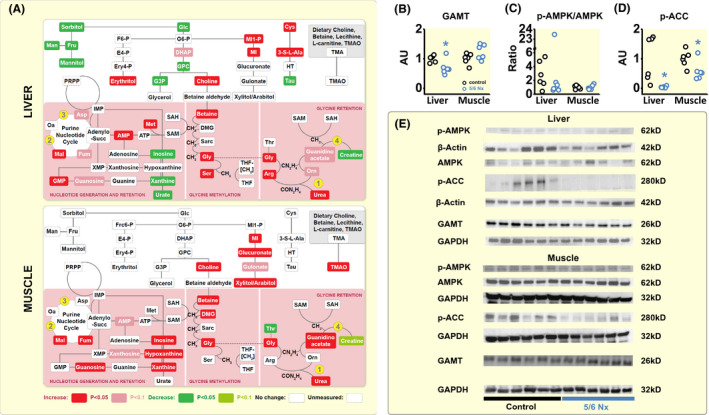
Reprioritization of purine and nitrogen metabolism in favour of methylamine production in rats with chronic renal failure. A, Metabolomic analysis of purine nitrogen and glycine utilization for betaine osmolyte production in the same rats as reported in Figure 3. The biosynthesis of S‐adenosyl‐methionine (SAM) in 5/6 Nx rats, which is responsible for the methylation of glycine during betaine synthesis, is promoted by the cataplerotic enzymes MDH1 (#2) and aspartate aminotransferase (#3) in the purine nucleotide cycle. Parallel increases in arginase activity (#1), in conjunction with reduced activity of guanidino‐acetate methyl transferase (GAMT; #4), reduces nitrogen transfer from arginine to creatine, and increases the availability of glycine as a substrate for organic osmolyte production. B, Densitometric quantification of guanidino‐acetate methyl transferase (GAMT) protein levels in liver and muscle of six control and six rats with 5/6 Nx. C, Densitometric quantification of the ratio of the phosphorylated and unphosphorylated for AMP‐activated kinase (AMPK) in the same rats. D, Densitometric quantification of phosphorylated acetyl‐CoA carboxylase (p‐ACC) protein levels in the same rats. E, Western Blots of AMPK, pAMPK, pACC and GAMT protein in liver and muscle of the control and the 5/6 Nx rats. *P < .05

The methylation of glycine (Gly) generates three important organic osmolytes that stabilize intracellular fluid content: 1‐methyl‐glycine, termed sarcosine (Sarc); 2‐methyl‐glycine (DMG), and 3‐methyl‐glycine, termed betaine (Figure 4A). Furthermore, cells can actively synthesize the glycine by tetrahydrofolate (THF)‐dependent methylation of serine (Ser). We found elevated serine, glycine, and betaine levels in the liver of 5/6 Nx rats (Figure 4A), indicating increased hepatic glycine methylation for organic osmolyte generation.

#### 2.4.1 Metabolic glycine retention

Successful betaine synthesis requires glycine as a substrate. Alternatively, glycine serves as a substrate for hepatic creatine generation. Catalysed by the enzyme arginine:guanidinoacetate amidino transferase, liver reversibly transfers an amidino (CH4N2) group from arginine (Arg) to glycine, resulting in guanidino acetate and ornithine (Orn) generation (Figure 4A). Catalysed by the enzyme guanidino acetate methyl transferase (GAMT), liver then methylates guanidino acetate to creatine (Figure 4A). Our 5/6 Nx rats showed decreased GAMT enzyme activity (Figure 4B) and reduced hepatic creatine levels, while their guanidino acetate, ornithine, glycine, arginine levels were elevated (Figure 4A), and their arginase‐driven urea synthesis was promoted (Figure 3B). We interpret these findings to show that 5/6 Nx rats reprioritized their hepatic arginine metabolism in favour of nitrogen transfer into urea osmolyte production, but enzymatically inhibited the alternative transfer of arginine‐derived amidino groups to creatine by reducing the rate of creatine production from guanidino acetate. Since amidino transfer from arginine to glycine is bidirectional, reduced GAMT enzyme activity thereby supported the availability of glycine as a substrate for betaine synthesis in 5/6 Nx rats.

#### 2.4.2 Glycine methylation

The enzymatic S‐adenosyl‐methionine (S‐Ado‐Met) / S‐adenosyl‐homocysteine (S‐Ado‐Hcys) methylation system transfers a methyl group from methionine to glycine, sarcosine, and dimethyl‐glycine to ultimately form betaine (Figure 4A). Organic osmolyte production by methylation of amino acids is intimately coupled with purine metabolism (Figure 4A). Adenyloscuccinate (AdenyloSucc) biosynthesis for AMP/ATP generation requires glycine, glutamine, and aspartate to form inosine monophosphate (IMP) from 5‐phosporibosyl pyrophosphate (PRPP). Aspartate not only donates the N‐1 atom to the IMP purine ring, but also donates the –NH2 group in the adenylosuccinate synthetase reaction that forms adenylo‐succincate from IMP. This reaction in turn releases the citric acid cycle intermediate fumarate, which, catalysed by cytosolic MDH‐1 and ASAT, forms a cytoplasmatic purine nucleotide cycle for sustainable adenylosuccinate/AMP production (Figure 4A). The increased MDH‐1 and ASAT enzyme activity we observed in 5/6 Nx rats (Figure 3C,D) therefore not only supported urea osmolyte synthesis (Figure 3A), but also parallel purine biosynthesis (Figure 4A).

Besides increased nitrogen‐ and energy‐intense nucleotide generation, the retention of nucleotides in tissue can be viewed as an additional energy‐efficient way to support the methylation machinery for organic osmolyte production. Uricotelic animals, such as reptiles, birds and insects, utilize uric acid for NH_4_^+^ elimination instead of urea. Our ureotelic 5/6 Nx rats with chronic renal water loss showed a different metabolic response (Figure 4A). Despite elevated hepatic AMP and elevated GMP/Guanosine levels, we found reduced inosine, xanthine and urate levels in the livers of 5/6 Nx rats, suggesting reduced xanthine oxidase‐mediated generation of urate. The rats with chronic renal failure thus metabolically promoted the nitrogen transfer necessary for nucleotide generation, and in parallel reduced hepatic elimination of nitrogenous waste from purine catabolism, resulting in elevated hepatic AMP levels.

An alternative explanation for the elevated AMP levels, however, is energy deficit, which lowers ATP levels and thereby increases tissue AMP content. We therefore next analysed the protein expression levels of the cellular energy level sensor, AMP‐kinase (AMPK). Despite elevated AMP levels, we found no increase in the ratio between phosphorylated and unphosphorylated AMPK in 5/6 Nx rats (Figure 4C), while the protein levels of phosphorylated acetyl‐CoA‐carboxylase (Figure 4D,E) were reduced. This finding suggests that 5/6 Nx rats reduced triglyceride synthesis and thereby shifted metabolism towards the observed exploitation of endogenous fat stores (Figure 3A), and that this metabolic fuel switch was sufficient to prevent energy‐deficiency with increased AMPK and p‐AMPK levels.

We interpret these findings to show that the body water conservation response in 5/6 Nx rats included preferential nitrogen transfer from arginine into urea solute production, which is coupled with reduced nitrogen transfer into creatine to promote glycine substrate accumulation for organic osmolyte production by glycine methylation. Reduction of hepatic uric acid synthesis additionally predisposes to purine retention, which supports the S‐adenosine‐methionine/homocysteine methylation machinery for organic osmolyte generation.

### 2.5 Extrarenal water conservation motif 3: experimental renal mass reduction promotes cutaneous vasoconstriction to reduce transepidermal water loss

**FIGURE 5:**
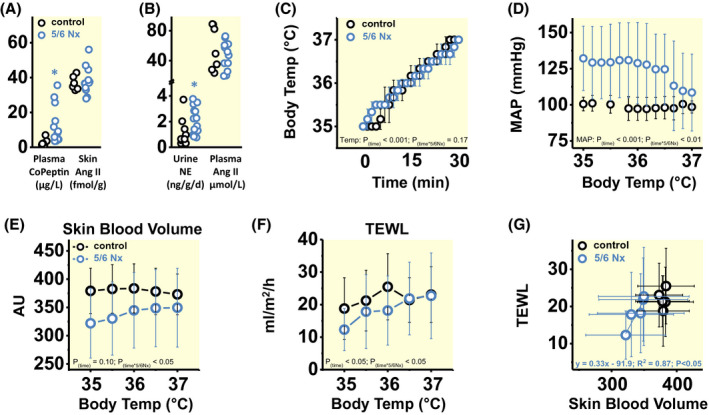
Rats with chronic renal failure reduce cutaneous blood flow to limit transepidermal water loss, albeit at the expense of arterial hypertension. A, Plasma copeptin and skin angiotensin II (Ang II) levels in the same rats as described in Figures 1 and 2. B, 24‐h urine norepinephrine (NE) excretion and plasma Ang 2 levels in control (n = 10) and 5/6 Nx rats (n = 14), in which we in parallel measured their plasma Ang 2 levels (controls: n = 7; 5/6 Nx: n = 12). C, Time‐dependent increase in body core temperature in anaesthetized control (n = 3) and 5/6 Nx rats (n = 3) exposed to 33°C ambient temperature; and D, Changes in intra‐arterial mean arterial blood pressure (MAP) in response to increasing body temperature. E, Cutaneous blood volume in response to increasing body core temperature in control (n = 5) and 5/6 Nx (n = 6) rats. F, Transepidermal water loss (TEWL) in response to increasing body core temperature in the same rats as in (E). G, Relationship between skin blood volume and TEWL in the same rats as in (E and F). Data are expressed as average ± SD and analysed by multivariate or univariate General Linear Model (A and B), univariate General Linear Model for Repetitive Measurements (C‐F), and simple linear regression (G). AU, arbitrary units. *P < .05

We finally studied the relationship between extrarenal water conservation, vasoconstriction and blood pressure level in our rats. Arterial hypertension is invariably coupled with an increase in peripheral vascular resistance. We found four important reasons why peripheral vascular resistance was increased in 5/6 Nx rats. First, metabolomic analysis showed increased levels of asymmetric and symmetric dimethyl‐arginine (ADMA; SDMA) and mono‐methyl‐arginine (MMA) in 5/6 Nx rats (Figure 3A). We interpret this finding to show that 5/6 Nx rats with activated methylation machinery not only generated more betaine from glycine (Figure 4), but also increased the methylation of arginine. The increased generation of the potent endothelial nitric oxide (NO) synthase inhibitors, MMA, ADMA and SDMA, results in reduced vascular NO production and increases vascular tone. Second, we found increased levels of γ‐glutamyl‐aminoacids (γ‐glutamyl‐AA), cysteine‐glutathione disulfide (GSH‐ss‐Cys), α‐hydroxybutyrate (α‐HB) in 5/6 Nx rats (Figure 3A), suggesting oxidative stress with endothelial dysfunction. Third, we found a 14‐fold increase in plasma copeptin levels in 5/6 Nx rats (Figure 5A), indicating increased antidiuretic hormone (ADH; vasopressin) action. Fourth, we found increased 24‐hour norepinephrine excretion (NE) in the urine of 5/6 Nx rats (Figure 5B), indicating increased sympathetic nerve tone. Only the peptide levels of the strong vasoconstrictor angiotensin II (Ang 2) were not different between control and 5/6 Nx rats, neither in skin tissue (Figure 5A), nor in plasma (Figure 5B).

Redistribution of blood volume and regulation of skin blood flow are closely related to the release of thermic energy and water across the skin. Vasodilation and the opening of arterio‐venous shunts translocate a relevant fraction of the total body blood volume from more central areas of the circulation into the vascular counter current system of the skin. The more blood is transferred from the body core to the surface of the skin, the more heat transfer can occur to prevent increasing core temperature during exercise and/or environmental heat exposure. The resulting heat dissipation for maintenance of normal body temperature, however, couples with an increase in transepidermal water loss.^13^ During thermoregulation, the diameter of cutaneous skin vessels is largely controlled by adrenergic norepinephrine‐driven vasoconstriction.^14^ This nerve‐driven vasoconstrictor tone is gradually withdrawn with increasing body temperature, resulting in vasodilation of cutaneous blood vessels for improved heat dissipation, which lowers blood pressure,^15^ albeit at the expense of increased transepidermal water loss.

We thus hypothesised that physiological adaptation to renal water loss in 5/6 Nx rats might include vasoconstriction of cutaneous blood vessels in an effort to reduce the cutaneous blood volume, which in turn would reduce the animals' transepithelial water loss for body water conservation, albeit at the expense of increased peripheral resistance and elevated blood pressure. We tested this hypothesis in additional experiments in anaesthetized rats in which we monitored blood pressure via an arterial line and increased their body temperature by exposing them to 33°C ambient temperature (Figure 5C‐G). Heat exposure increased body core temperature in both control and 5/6 Nx rats (Figure 5C). However, with an increase in body temperature above 36.5°C, blood pressure in anaesthetized 5/6 Nx rats rapidly fell to the control level (Figure 5D). This finding suggests that with increasing body temperature, pre‐existing cutaneous vasoconstriction in 5/6 Nx rats ceased to allow for improved dermal heat dissipation, and that the opening of skin vessels for improved heat transfer was sufficient to reduce blood pressure back to the control level.

We therefore next tested the hypothesis that 5/6 Nx rats showed reduced cutaneous blood pooling and lower transepidermal water loss, which would cease with increasing body core temperature. Rats with chronic renal failure at 35⁰C body temperature showed lower skin blood volume than controls, which increased back to the higher control level with increasing body temperature at 36⁰C‐37⁰C (Figure 5E). With lower skin blood volume than controls, 5/6 Nx rats showed the expected reduction in transepidermal water loss (TEWL; Figure 5F), which increased back to the control level once the skin blood volume was increased with higher body temperature. The body temperature‐driven reopening of skin vessels with its surrogate marker of increased skin blood volume abolished the cutaneous water conservation response in 5/6 Nx rats (Figure 5G). Rats with chronic renal failure additionally showed a tendency towards higher skin blood flow velocity, which was not modulated by changes in body temperature (Figure S3A). Therefore, 5/6 Nx rats showed no direct relationship between skin blood flow velocity and the observed body temperature‐driven changes in TEWL (Figure S3B). The detectable increase in cutaneous blood flow that occurred with increasing body temperature in 5/6 Nx rats (Figure S3C), which was paralleled by increased TEWL (Figure S3D), was mainly attributable to the normalization of cutaneous blood pooling with increasing body temperature (Figure 5E).

We interpret these findings to show that 5/6 Nx rats reduced cutaneous blood flow and thereby limited their transepidermal water loss in an effort to compensate for their renal water loss. This adaptive physiological dermal water conservation response was reversible once 5/6 Nx rats additionally translocated more blood volume from the central circulation to the skin surface for body heat dissipation. The observation that the reversal of this transepidermal water conservation response, which requires dilation of cutaneous skin vessels, was paralleled by a normalization of blood pressure suggests that cutaneous vasoconstriction may be an important causal pathophysiological factor in the development of renal hypertension.

## 3 DISCUSSION

Studying urine solute and water excretion in conjunction with body solute content, water content and telemetric blood pressure recording, we report three findings that are antipodal to established thinking in medical salt and water physiology and hypertension‐related research.

*First*, we found that our rats with chronic renal failure excreted the appropriate amount of Na^+^, K^+^ and urea solutes in their urine, and they instead had to adjust to renal water loss because of an inability to concentrate their urine. Our data confirm an earlier report demonstrating that surgical 5/6 renal mass ablation leads to renal water loss without commensurate increases in 24‐hour Na^+^ and K^+^ solute excretion.^16^ These findings are antipodal to the idea that experimental chronic renal failure results in an inability to excrete sufficient amounts of Na^+^, K^+^, urea and water.

*Second*, we found that the rats’ body response to this dehydration stress was characterized by a primarily urea‐driven increase in plasma osmolality and the accumulation of Na^+^, K^+^, urea and other organic osmolytes in the tissues. In line with our previous studies,^17^, ^18^, ^19^, ^20^ but not in line with a recent study analysing small tissue samples in WKY and spontaneously hypertensive rats,^21^ we found that the (Na^+^+K^+^)‐to‐water ratio in the rat skin is substantially higher than the [Na^+^+K^+^] concentration in plasma. This result is expected,^22^ because the keratinocyte layer generates a regional microenvironment with high Na^+^ and K^+^ concentration,^23^, ^24^, ^25^, ^26^, ^27^, ^28^, ^29^, ^30^ which is non‐invasively detectable in‐vivo in humans with ultra‐high field 23NaMRI.^31^ Our simple chemical analysis cannot address the question whether these local cutaneous concentration gradients were altered in 5/6 Nx rats. The observed accumulation of Na^+^, K^+^ and water per unit tissue mass in 5/6 Nx rats occurred at constant absolute skin Na^+^ and K^+^ content because of a reduction in skin tissue mass, and therefore could not have been detected even in most accurate salt and water balance studies.^32^ The observed changes in tissue solute and water composition and plasma osmolality occurred in the absence of any change in plasma Na^+^ concentration. These findings are antithetical to the idea that body solute and water physiology follows a simple passive equilibrium, in which the pattern of renal solute and water excretion would easily predict the changes in body solute and water composition.

*Third*, we found that the blood pressure level in 5/6 Nx was directly related to the degree of their renal urine concentration deficit, which resulted in higher urine volumes and predisposed the animals to dehydration. This finding suggests that the cause of the rats’ arterial hypertension develops in the context of an adaptive physiological body response designed to prevent dehydration. This conclusion is antithetical to the concept that the pathophysiological cause of arterial hypertension originates from a body response that is designed to prevent body Na^+^ and water overload during states of reduced renal Na^+^ and water excretion.^3^ This theory is not easily applicable to explain the hypertension in our 5/6 Nx rats, because it is difficult to hypothesize that an organism that requires adaptive physiological water conservation to prevent dehydration would utilize physiological adaptive pressure natriuresis which would even further worsen renal water loss (Figure S4). Furthermore, our data suggest that compensatory vasoconstriction in an effort to limit further water loss across intact biological barriers may functionally explain the observed hypertension in 5/6 Nx rats, because successful stabilization of body water content at the expense of higher vascular tone will inevitably increase systemic blood pressure levels. We suggest that the resulting hypertension was rather an adverse side effect of successful water conservation by vasoconstriction.

While standard medical physiology literature on the principles of body solute and water homeostasis did not allow us to interpret these seemingly paradoxical study results, our data are in line with an alternative view on the principles of solute and water physiology that is well‐established in the zoological sciences. Physiological adaptation to water shortage, termed *aestivation*, is an evolutionary preserved physiological‐adaptive survival strategy that allowed life to move out of the aquatic environments to the arid land.^33^, ^34^, ^35^ Amphibians that enter brackish water with higher salinity than their usual environment experience acute movement of water out of their body, predisposing them to dehydration stress.^36^ In an effort to resist body water loss, *aestivators* produce and accumulate solutes in their bodies. *Aestivators* limit renal and dermal excretion of urea and thereby increase urea levels in tissues and plasma.^37^ Besides urea, they accumulate Na^+^, Cl^‐^ and other organic osmolytes, such as amino acid derivatives like methylated glycine products in their biological barriers. The resulting solute accumulation allows *aestivators* to maintain body hydration in hostile ‘dehydrating’ environments.^36^, ^54^ Aestivating lungfish or cartilaginous fishes in high‐salinity marine environments primarily utilize urea for water conservation, without a notable contribution of Na^+^ solutes.^55^, ^56^, ^57^, ^58^ The perhaps major advantage of such urea‐ and other organic osmolyte‐driven water conservation over Na^+^ and K^+^ solute‐driven water conservation is that organic osmolytes can be generated by exploitation of endogenous fuel stores, which makes the physiological‐adaptive process of body water conservation more autonomous and less reliant on dietary environmental factors. Successful water conservation therefore not only requires efficient transporter‐driven recycling of Na^+^, K^+^, urea and other organic osmolyte solutes in the biological barriers of the kidney, gut and skin. During dehydration stress body water conservation additionally relies on increased production of intracellular organic osmolytes.^59^ De‐novo synthesis of urea solutes, which readily equilibrate in the extra‐ and intracellular space, has been the most frequently studied dehydration response in *aestivators*. Because organic solute production is both energy intense and nitrogen dependent, its increased biosynthesis as a solute for water conservation relies on the catabolism of protein.^60^, ^61^, ^62^, ^63^, ^64^, ^65^, ^66^, ^67^, ^68^ Thus, successful water conservation is paralleled by substantial fuel switches in systemic energy metabolism.^7^, ^8^

The word ‘*aestivation*’ derives from the Latin word for ‘summer’ (aestas) or ‘heat’ (aestus) and describes a series of such evolutionarily conserved metabolic switches that allow organisms to survive arid conditions with reduced fuel and restricted water availability.^69^, ^70^, ^71^ Combining modern metabolomics analysis with traditional techniques for clinical investigation of body fluid homeostasis, we show that these key metabolic *aestivation* principles are not restricted to the metabolism of plants, fish, toads, frogs, salamander or other amphibia, but also occur in our mammalian rodent model of experimental renal failure. This alternative physiological view, seemingly paradoxical at first gance, ultimately provides with a simple and straightforward explanation for the development of sarcopenia and elevated blood pressure in chronic renal failure.

First, we find that the “renal uremic phenotype” in chronic kidney failure with accumulation of urea, salt, and water in the body is not explained by reduced excretion of solutes and water by diseased kidneys, but instead is understandable as a powerful extrarenal adaptive *aestivation* response that is designed to prevent lethal dehydration by accumulation and production of solutes in body tissues. The dependency of the underlying water conservation patterns on energy and nitrogen transfer for organic osmolyte production explains why chronic renal failure will ultimately lead to reduced muscle mass (Figure S5).

Second, we find that the “renal hypertension phenotype” is difficult to explain by a complicated adaptive body response designed to overcome an inability of the kidneys to excrete sufficient amounts of salt and water.^1^, ^2^, ^3^, ^72^ Rather, the blood pressure increase in our rats with chronic renal failure can be understood as an adaptive water conservation response that utilizes vascular vasoconstriction to limit body water loss. The idea that local control of skin blood volume and skin blood flow may be a critical component for systemic blood pressure regulation in essential hypertension is not new,^13^, ^80^ and the importance of skin barrier physiology for systemic blood pressure control has been highlighted in several recent experimental studies.^15^, ^20^, ^29^, ^73^, ^74^, ^75^, ^81^, ^82^ Recapitulating this concept in rats with chronic renal water loss adds the substantiated hypothesis that cutaneous vasoconstriction might be a relevant component of a multifactorial, systemic, adaptive physiological water conservation response which limits body water loss across the skin, albeit at the expense of arterial hypertension (Figure S5). In line with the concept that reduced NO production in keratinocytes may lead to cutaneous vasoconstriction and thereby cause essential hypertension,^15^, ^73^, ^74^, ^77^, ^78^, ^79^, ^82^ our metabolomic analysis suggests that 5/6 Nx rats systemically reprioritize nitrogen transfer from arginine in favour of urea and other organic osmolyte solute production for water conservation, while nitrogen transfer from arginine to NO is inhibited (Figures 3 and 4). Furthermore, the increased ADH release as part of the body water conservation response in 5/6 Nx rats may not only have promoted residual free water reabsorption in their remnant 1/6 kidney mass, but is also a strong vasoconstrictor which reduces cutaneous blood flow,^83^, ^84^, ^85^, ^86^, ^87^, ^88^, ^89^, ^90^, ^91^ reduces transepidermal water loss ^92^, ^93^, ^94^, ^95^, ^96^ and increases blood pressure.^97^

However, regulation of skin blood volume and flow is dominated by adrenergic norepinephrine‐driven vasoconstriction.^14^ Edholm et al reported earlier that extant vasoconstrictor nerve activity ceases with acute heat stress, and cutaneous vasodilation ensues.^98^ In line with this observation, the systemic body water conservation response in our 5/6 Nx rats also included increased norepinephrine excretion in the urine, indicating increased sympathetic nerve activity. The observed increase in reactive oxygen species generation (Figure 3A), the reprioritization of arginine metabolism with endogenous eNOS inhibition (Figure 3A), and elevated ADH levels (Figure 5A) suggest that the renal hypertension in 5/6 Nx rats develops with multifactorial generalized vasoconstriction, which might be difficult to reverse (resistant hypertension). We were surprised to find that despite this generalized predisposition to increased vascular tone, we found rapid correction of reduced skin blood volume (Figure 5E), reduced transepidermal water loss (Figure 5F) and elevated blood pressure (Figure 5D) once our 5/6 Nx rats needed to dissipate more heat with increasing body temperature, albeit at the expense of increased transepidermal water loss (Figure 5G and Figure S6). This finding suggests that modulation of sympathetic/parasympathetic nerve activity could acutely override the multifactorial predisposition to vasoconstriction once improved heat dissipation was necessary. We hypothesize that any chronic predisposition to dehydration will predispose to dermal vasoconstriction, which may cause chronic blood pressure elevation. As we did not measure blood flow in the other barriers gut, kidney and lungs, it remains unclear to which extent the observed water‐conserving and blood pressure‐increasing effect in 5/6 Nx rats is explained by selective changes in the skin circulation.

### 3.1 Physiological relevance

Despite the fundamental importance of *aestivation* as a physiological response for body water conservation, its metabolic principles are not usually a topic of structured medical education. A detailed description of *aestivation* and its underlying regulatory mechanisms can be found elsewhere.^34^, ^99^ We have reported earlier that mice with intact renal function show comparable *aestivation*‐like water conservation patterns to limit Na^+^‐ and Cl^‐^‐driven osmotic diuresis on a high‐salt diet.^7^ It is tempting to speculate that additional dietary salt loading in 5/6 Nx rats might augment the renal water loss and trigger an even more pronounced extrarenal water conservation response with more severe exploitation of muscle protein for organic solute generation, more pronounced dermal water conservation, and exaggerated arterial hypertension.

Besides the here described disturbance in free water reabsorption, renal water loss can occur because of osmotic diuresis during excess Na^+^ (with accompanying anions), K^+^ (with accompanying anions), urea or glucose solute excretion. We hypothesize that all these states will be accompanied by the activation of various *aestivation* motifs for adaptive physiological water conservation. *Aestivation* metabolism is an evolutionary survival strategy that, besides water conservation, induces cellular hypometabolism and thereby preserves organ function. Increased urinary Na^+^ and glucose excretion with SGLT2 inhibitor treatment likely triggers such adaptive *aestivation* responses, which improve cellular lifespan and thereby may contribute to the cardiorenal protection that has been observed with this class of therapeutic agents.^100^ We currently study whether treatment with loop diuretics, thiazides, mineracorticoid receptor antagonists and/or aquaporin 2 inhibitors similarly triggers *aestivation* motifs for chronic water conservation. We conclude that adaptive *aestivation* metabolism in the various body organs is an under‐studied research area that, besides hypertension and muscle mass loss in chronic renal failure, may explain many otherwise unexplainable phenomena in medicine.

## 4 MATERIALS AND METHODS

### 4.1 Animal studies 1‐4

The Animal Research Committee of Kagawa University and Emory University approved the experimental protocols and procedures. We used male Sprague‐Dawley (SD) rats (Japan SLC Inc, Shizuoka, Japan and Charles River Laboratories, Wilmington, MA). The animals were housed under controlled temperature (24°C ± 2°C) and humidity (55% ± 5%) on a 12‐hour light/dark cycle. We fed the animals rodent diets with either 0.2% Na^+^, 0.9% K^+^ and 23.1% protein content (Kagawa University experiments), or with 0.4% Na^+^, 1.2% K^+^ and 28.5% protein content (Emory University experiments).

### 4.2 Experimental protocol 1

We implanted radiotelemetry transmitters (Data Sciences International (DSI), St Paul, MN, USA) into the rats and performed a left kidney resection of the upper and lower thirds (n = 12) or a sham surgery (n = 6) at 7 weeks of age in one surgical procedure. Then, we performed a right uni‐nephrectomy or a sham surgery at 8 weeks of age. Control rats and 5/6Nx rats were provided free access to standard rat chow and to tap water. After one week of recovery, we measured body weight, daily food and water intake. We measured the rats’ blood pressure by radiotelemetry on day 0, day 6 and day 13 after recovery. On Day 14 after recovery, we placed the rats into metabolic cages, collected their urine samples and measured their food intake for 24 hours. We terminated the experiment after metabolic cage urine collection, sacrificed the rats and harvested tissues.

### 4.3 Experimental protocol 2

We performed a left kidney resection of the upper and lower thirds (n = 10) or a sham surgery (n = 6) at 7 weeks of age. Then, we performed a right uni‐nephrectomy or a sham surgery at 8 weeks of age. Control rats and 5/6Nx rats were provided free access to standard rat chow and to tap water. 3 weeks after uni‐nephrectomy or sham surgery, we placed the rats into metabolic cages, collected their urine samples and measured their food intake for 24 hours. We then sacrificed the rats and harvested tissues for western blot, enzyme activity assays and metabolome analysis.

### 4.4 Experimental protocol 3

We generated control (n = 3) and 5/6Nx (n = 3) rats as described above in experimental protocol‐2. At 11 weeks of age, we anaesthetized the rats using 1.0%‐3.0% isoflurane and inserted an arterial line. We increased room temperature to 33 degrees, then measured their rectal temperature, heart rate and blood pressure every minute for consecutive 30 minutes.

### 4.5 Experimental protocol 4

We generated control (n = 5) and 5/6Nx (n = 6) rats as described above in the experimental protocol‐2. At 11 weeks of age, we anaesthetized the rats using 1.0%‐3.0% isoflurane, increased room temperature to 33 degrees, and measured rectal temperature, skin blood velocity/flow/blood mass and transepidermal water loss every six minutes for consecutive 30 minutes.

### 4.6 Blood pressure measurements: experimental protocol 1

We used a radiotelemetry system (DSI) in experimental protocol 1 as described previously.^101^ A radiofrequency transmitter (PA‐C40; DSI) and a receiver (RPC‐1; DSI) were used to measure blood pressure and heart rate. Data were collected and analysed using Dataquest ART version 4.3 (DSI). *Experimental protocol 3:* We inserted a polyethylene catheter (PE‐60; Becton Dickinson and Company, Sparks, MD, USA) into the abdominal aorta via the right femoral artery for blood pressure measurement and analysed data using Power Lab (PowerLab 8/30; AD Instruments).

### 4.7 Urine collection (experimental protocol 1 and 2)

The rats were placed into metabolic cages with free access to regular diet and tap water, and 24 hours of urine volume and fluid intake were measured. All rats underwent a 12‐hour acclimatization period in metabolic cages before urine collection.

### 4.8 Electrolyte and osmolality measurement (experimental protocol 1 and 2)

Sodium and potassium concentration in plasma and urine was measured by using an automated analyser (7020‐Automatic Analyzer; Hitachi High‐Technologies). Plasma and urine osmolality were measured by vapour pressure osmometry (Wescor).

### 4.9 Skin and carcass electrolyte and water content (experimental protocol 1)

Skin and carcass samples were desiccated at 90°C for 72‐hours as described previously.^102^ Water content was calculated from the difference between wet weight and dry weight. We ashed the dry samples at a maximum of 450°C for total 92‐hours. Then, we further ashed the samples at 600℃ for 42‐hours. We dissolved the skin ashes in 20 mL of 10% HNO3 and the carcass ashes in 50 mL of 5% HNO3. We measured sodium and potassium concentration of these samples by an atomic absorption spectrometer (AA‐7000; Shimadzu).

### 4.10 Urinary norepinephrine measurement (experimental protocol 1)

We collected urine in the tubes including 2% of 6N HCl. Urinary norepinephrine concentration was measured using high‐performance liquid chromatography (FALCO biosystems Ltd).

### 4.11 Angiotensin II measurement (experimental protocol 1)

We measured Ang II levels of plasma and skin by radioimmunoassay (RIA) as described in more detail previously.^103^ Frozen skin tissues were homogenized in 10 mL ice‐cold methanol. The homogenate was centrifuged at 3000 rpm for 10 minutes, and the supernatant was evaporated overnight. Then, the evaporated skin samples were solubilized in 6 mL of angiotensin assay buffer which is composed of 50 mmol/L sodium phosphate, 1 mmol/L ethylenediaminetetraacetate (EDTA), 0.25 mmol/L thimerosal, 2.5 mg/mL of bovine serum albumin. Additionally, we collected blood samples into the tube including the inhibitor cocktails, which is composed of 30 μL of 500 mmol/L EDTA, 15 μL of 1 mmol/L enalaprilat, and 30 μL of 125 mmol/L o‐phenanthroline and 0.2 mmol/L pepstatin in 95% ethanol, centrifuged at 3000 rpm for 20 minutes, and collected the supernatant. We then extracted angiotensin II peptide using phenyl‐bonded, solid‐phase extraction columns from the plasma samples (Bond Elut, Agilent Technologies Inc) and the skin samples (ISOLUTE, Biotage). Next, the extracted samples were evaporated overnight and solved in 500 µL of the Angiotensin assay buffer. We finally measured angiotensin II peptide by RIA.

### 4.12 Copeptin and enzyme activity assay (experimental protocol 1 and 2)

Plasma copeptin level (Peninsula Laboratories), Arginase (Sigma‐Aldrich), malate dehydrogenase (MDH)‐1 (Abcam) and aspartate aminotransferase (ASAT) (Abcam) activities were measured by commercially available kits according to the manufacturers’ protocols. We have described the detailed protocol including sample preparation previously.^7^

### 4.13 Western blotting analysis (experimental protocol 2)

We quantified protein expression in liver and muscle by Western blotting as described previously.^7^ We used the following commercially available primary antibodies: rabbit polyclonal anti‐guanidinoacetate N‐methyltransferase antibody (1:1000; Abcam; ab229467); rabbit polyclonal anti–p–acetyl‐CoA carboxylase (Ser79) antibody (1:2000 for liver, 1:6000 for muscle; Cell Signaling Technology, Danvers, MA; 3661); rabbit polyclonal anti–p–AMPKα (Thr172) antibody (1:2000 for liver, 1:6000 for muscle; Cell Signaling Technology; 4188); rabbit polyclonal anti‐AMPKα antibody (1:2000 for liver, 1:4000 for muscle; Cell Signaling Technology; 1532); and HRP‐conjugated anti–β‐actin (1:10,000; Sigma‐Aldrich; A3854) or anti‐GAPDH antibody (1:10,000; Sigma‐Aldrich; G9295).

### 4.14 Metabolomic profile analysis (experimental protocol 2)

Liquid nitrogen snap‐frozen liver and muscle samples from 6 sham and 10 rats with experimental renal mass reduction were aliquoted and used for detection and relative quantification of metabolites by Metabolon as previously described.^104^ All metabolites except for urea were measured by ultra‐high‐performance LC‐MS/MS (UPLC‐MS/MS) (Metabolyn; Metabolon Inc). We focused on understanding key metabolic components of glucose, amino acid, fatty acid, urea, methyl‐glycine and purine metabolism.

### 4.15 Adaptive circulatory water conservation response in the skin (experimental protocol 4)

We quantified transepidermal water loss at the hair‐removed lateral back skin by measuring cutaneous water evaporation (VAPO SCAN AS‐VT100RS, ASCH JAPAN Co., Ltd). The "VAPO SCAN AS‐VT100RS" has a closed chamber system, which quantifies transepidermal water loss by the changes in the absolute humidity in the chamber system independently of the external environment.^105^, ^106^, ^107^ We quantified skin blood volume, skin blood flow and skin blood flow velocity at the earflap by Doppler sonography (FLO‐C1, OMEGAWAVE).^108^ Quantification of transepidermal water loss and skin Doppler sonography in experimental animals requires anaesthesia, which leads to peripheral vasodilation and thereby increases TEWL, reduces body temperature and lowers blood pressure. The following adaptive changes in skin circulation, cutaneous water conservation and blood pressure during our rewarming experiments therefore occurred from a slightly hypothermic starting point.

### 4.16 Statistical analysis

We used SPSS Version 25 for data analysis. Data are expressed as averages ± SD. Data and the statistical analysis syntax file are available online at https://data.mendeley.com/datasets/4ty475fmjy/draft#folder‐4b192be9‐2ab6‐48c1‐8bee‐1035ae6fc54c

We analysed the relationship between the sum of Na^+^, K^+^ and urea solutes and total solutes in the urine by linear or polynomial regression, and the relationship between urine osmolality and urine volume or water intake and urine volume by polynomial regression. We analysed for differences in the relative contribution of Na^+^, K^+^ or urea solutes to total urine solutes between control and 5/6 Nx rats by multivariate General Linear Model analysis (Figure 1).

We analysed the relationship between urine osmolality and mean arterial blood pressure, the relationship between urine volume and mean arterial pressure, the relationship between the sum of skin Na^+^+K^+^ content and skin water content, and the relationship between the sum of skin Na^+^+K^+^ content and mean arterial blood pressure by linear regression in control and in 5/6 Nx rats. In case that both animal groups showed comparable relationships, we calculated and plotted the regression across both groups. In case that none or only one of the groups showed a significant relationship between the parameters, we only plot the resulting parameter associations without reporting a regression line (Figure 2).

Differences in tissue metabolite content between control and 5/6 Nx rats were analysed by Welch's Two‐Sample t‐test. We analysed differences in tissue arginase, malate dehydrogenase 1 and aspartate aminotransferase activity by non‐parametric Mann‐Whitney U Test. We analysed differences in guanidinoacetate methyltransferase, phosphorylated acetyl‐CoA carboxylase and phosphorylated AMP kinase tissue protein level by non‐parametric Mann‐Whitney U Test (if sample not normally distributed), or by unpaired T‐Test (if sample normally distributed), depending on the result of One‐Sample Kolmogorov‐Smirnov‐Test for normal distribution (Figures 3 and 4).

We analysed differences in plasma copeptin, skin angiotensin II, 24 urine norepinephrine and plasma angiotensin II levels between control and 5/6 Nx rats by multivariate or univariate General Linear Model analysis. We analysed the difference in body temperature, mean arterial blood pressure, skin blood volume, transepidermal water loss, skin blood flow velocity and skin blood flow between control rats and 5/6 Nx rats in response to increased environmental temperature by General Linear Model for Repeated Measurements analysis (univariate ANOVA analysis) (Figure 5 and Figure S3).

We analysed the differences in 24‐hours urine solute and water excretion and the differences in tissue Na^+^, K^+^ and water content between control and 5/6 Nx rats by multivariate General Linear Model analysis (Tables 1 and 2).

## CONFLICT OF INTEREST

The authors declare no competing interests.

## AUTHOR CONTRIBUTIONS

JJK, NM and KK designed and conducted the experiments, conducted sample analyses, and analysed the data; JW, AM, KT‐M. and J.‐PK contributed to the project analysis approach and data interpretation; SM and SD conducted sample analyses; J.‐P.K, M.R, SK, JMS, JDK, KFH and FCL contributed to project design and edited the manuscript; JMS, JDK, JLB, AN and DN designed and implemented the animal experiments; KK and JT articulated the research hypothesis, designed and implemented the research approach, analysed and interpreted the data, and wrote the manuscript.

## Supporting information

Fig S1‐S6

Table S1

Table S2
