# Supplementary material for: Adaptive physiological water conservation explains hypertension and muscle catabolism in experimental chronic renal failure
Source: Acta Physiol (Oxf). 2021 Mar 7;232(1):e13629. doi: 10.1111/apha.13629 (PMC8244025; doi:10.1111/apha.13629)
Supplement: Supplementary file 1 — Fig S1‐S6 [file APHA-232-e13629-s002.pptx]

## Slide 1
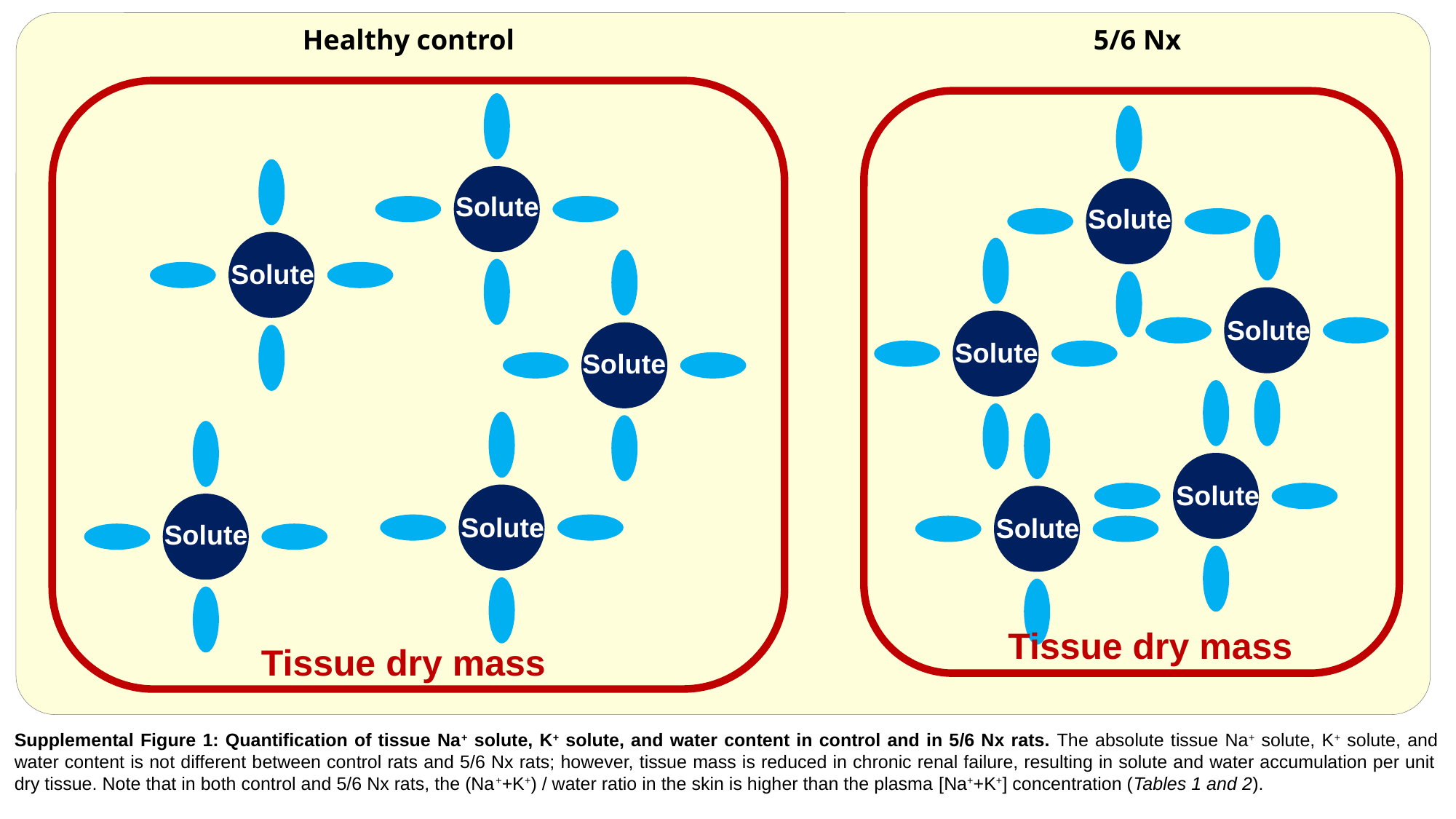

5/6 Nx
Solute
Solute
Solute
Solute
Solute
Tissue dry mass
Healthy control
Solute
Solute
Solute
Solute
Solute
Tissue dry mass
Supplemental Figure 1: Quantification of tissue Na+ solute, K+ solute, and water content in control and in 5/6 Nx rats. The absolute tissue Na+ solute, K+ solute, and water content is not different between control rats and 5/6 Nx rats; however, tissue mass is reduced in chronic renal failure, resulting in solute and water accumulation per unit dry tissue. Note that in both control and 5/6 Nx rats, the (Na++K+) / water ratio in the skin is higher than the plasma [Na++K+] concentration (Tables 1 and 2).

## Slide 2
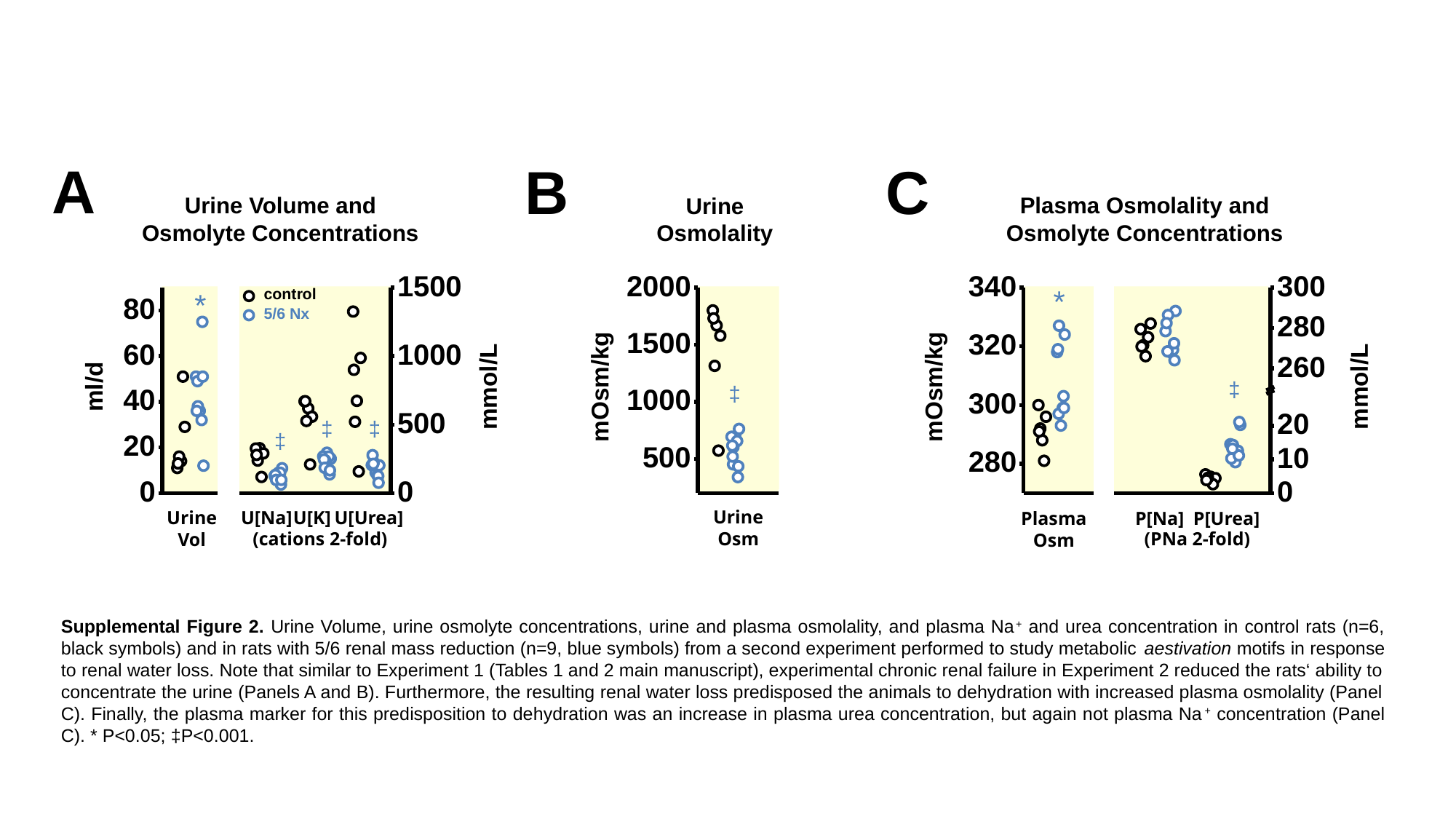

A
B
C
Urine Volume and
Osmolyte Concentrations
Plasma Osmolality and
Osmolyte Concentrations
Urine
Osmolality
*
*
ml/d
mmol/L
mOsm/kg
mOsm/kg
mmol/L
‡
‡
‡
‡
‡
Urine
Osm
U[Na]
U[K]
U[Urea]
(cations 2-fold)
Urine
Vol
P[Na]
P[Urea]
(PNa 2-fold)
Plasma
Osm
Supplemental Figure 2. Urine Volume, urine osmolyte concentrations, urine and plasma osmolality, and plasma Na+ and urea concentration in control rats (n=6, black symbols) and in rats with 5/6 renal mass reduction (n=9, blue symbols) from a second experiment performed to study metabolic aestivation motifs in response to renal water loss. Note that similar to Experiment 1 (Tables 1 and 2 main manuscript), experimental chronic renal failure in Experiment 2 reduced the rats‘ ability to concentrate the urine (Panels A and B). Furthermore, the resulting renal water loss predisposed the animals to dehydration with increased plasma osmolality (Panel C). Finally, the plasma marker for this predisposition to dehydration was an increase in plasma urea concentration, but again not plasma Na+ concentration (Panel C). * P<0.05; ‡P<0.001.

## Slide 3
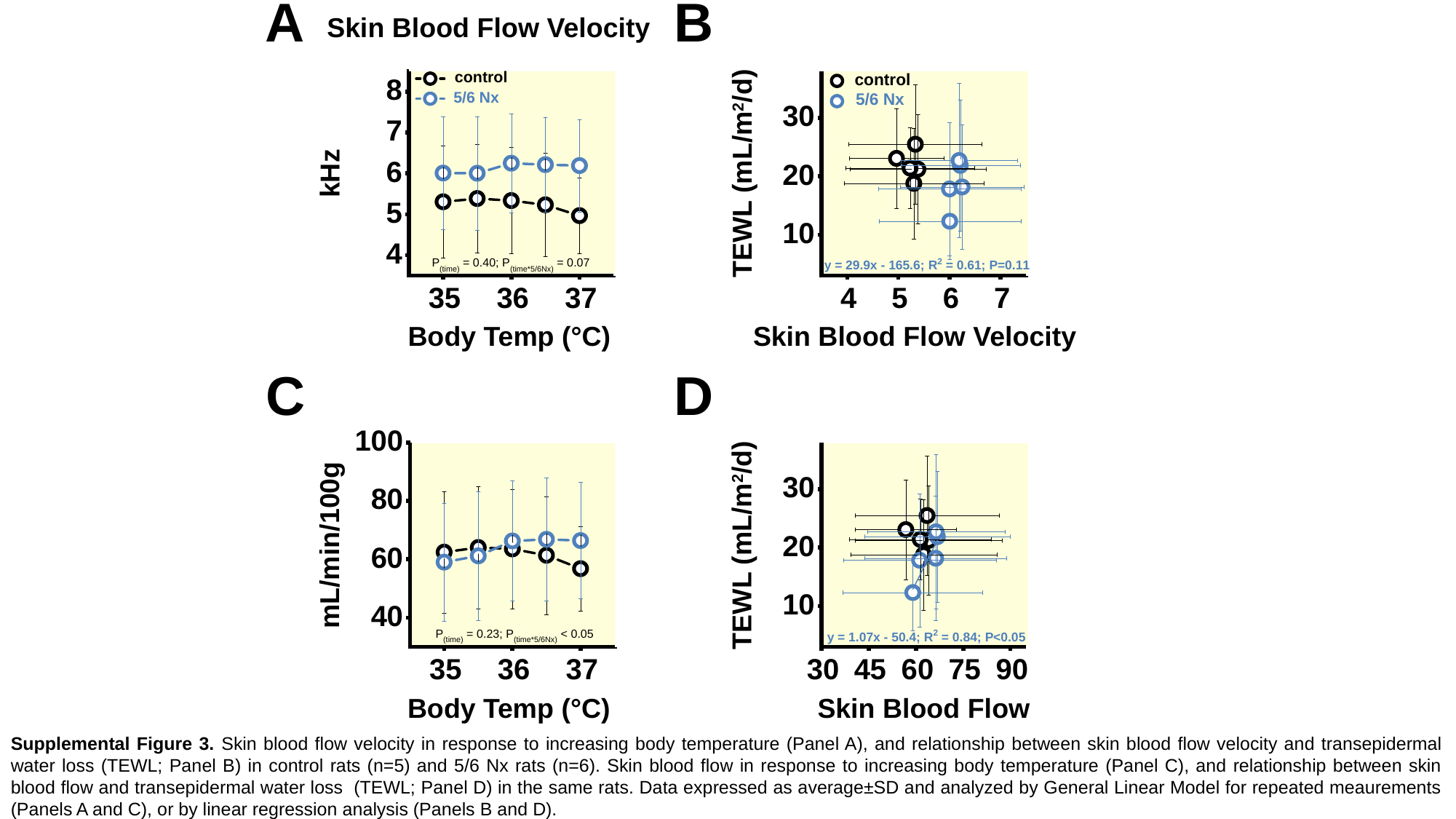

A
B
Skin Blood Flow Velocity
kHz
Body Temp (°C)
TEWL (mL/m2/d)
Skin Blood Flow Velocity
C
D
TEWL (mL/m2/d)
Skin Blood Flow
mL/min/100g
Body Temp (°C)
Supplemental Figure 3. Skin blood flow velocity in response to increasing body temperature (Panel A), and relationship between skin blood flow velocity and transepidermal water loss (TEWL; Panel B) in control rats (n=5) and 5/6 Nx rats (n=6). Skin blood flow in response to increasing body temperature (Panel C), and relationship between skin blood flow and transepidermal water loss (TEWL; Panel D) in the same rats. Data expressed as average±SD and analyzed by General Linear Model for repeated meaurements (Panels A and C), or by linear regression analysis (Panels B and D).

## Slide 4
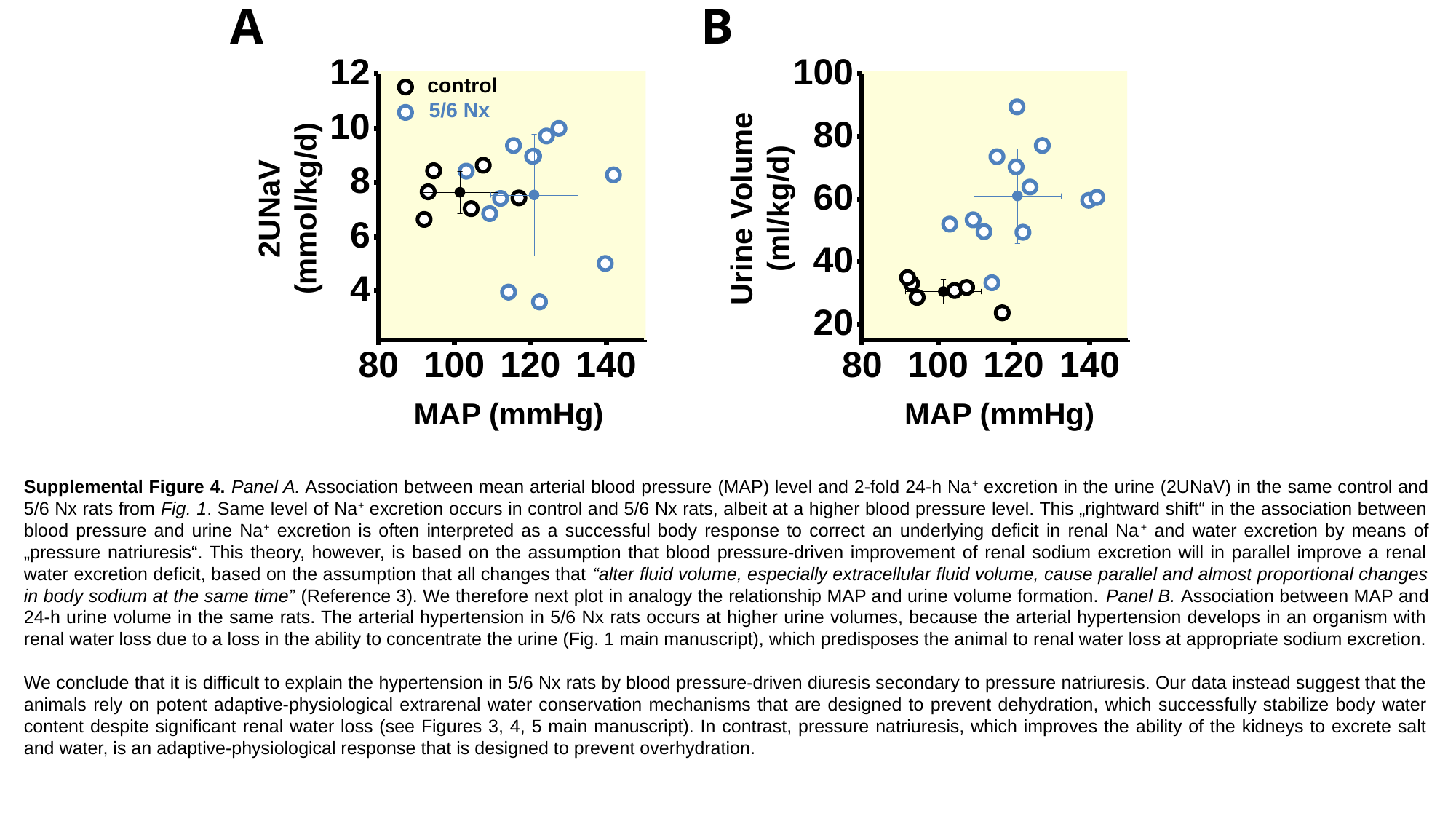

A
B
Urine Volume
(ml/kg/d)
2UNaV
(mmol/kg/d)
MAP (mmHg)
MAP (mmHg)
Supplemental Figure 4. Panel A. Association between mean arterial blood pressure (MAP) level and 2-fold 24-h Na+ excretion in the urine (2UNaV) in the same control and 5/6 Nx rats from Fig. 1. Same level of Na+ excretion occurs in control and 5/6 Nx rats, albeit at a higher blood pressure level. This „rightward shift“ in the association between blood pressure and urine Na+ excretion is often interpreted as a successful body response to correct an underlying deficit in renal Na+ and water excretion by means of „pressure natriuresis“. This theory, however, is based on the assumption that blood pressure-driven improvement of renal sodium excretion will in parallel improve a renal water excretion deficit, based on the assumption that all changes that “alter fluid volume, especially extracellular fluid volume, cause parallel and almost proportional changes in body sodium at the same time” (Reference 3). We therefore next plot in analogy the relationship MAP and urine volume formation. Panel B. Association between MAP and 24-h urine volume in the same rats. The arterial hypertension in 5/6 Nx rats occurs at higher urine volumes, because the arterial hypertension develops in an organism with renal water loss due to a loss in the ability to concentrate the urine (Fig. 1 main manuscript), which predisposes the animal to renal water loss at appropriate sodium excretion.
We conclude that it is difficult to explain the hypertension in 5/6 Nx rats by blood pressure-driven diuresis secondary to pressure natriuresis. Our data instead suggest that the animals rely on potent adaptive-physiological extrarenal water conservation mechanisms that are designed to prevent dehydration, which successfully stabilize body water content despite significant renal water loss (see Figures 3, 4, 5 main manuscript). In contrast, pressure natriuresis, which improves the ability of the kidneys to excrete salt and water, is an adaptive-physiological response that is designed to prevent overhydration.

## Slide 5
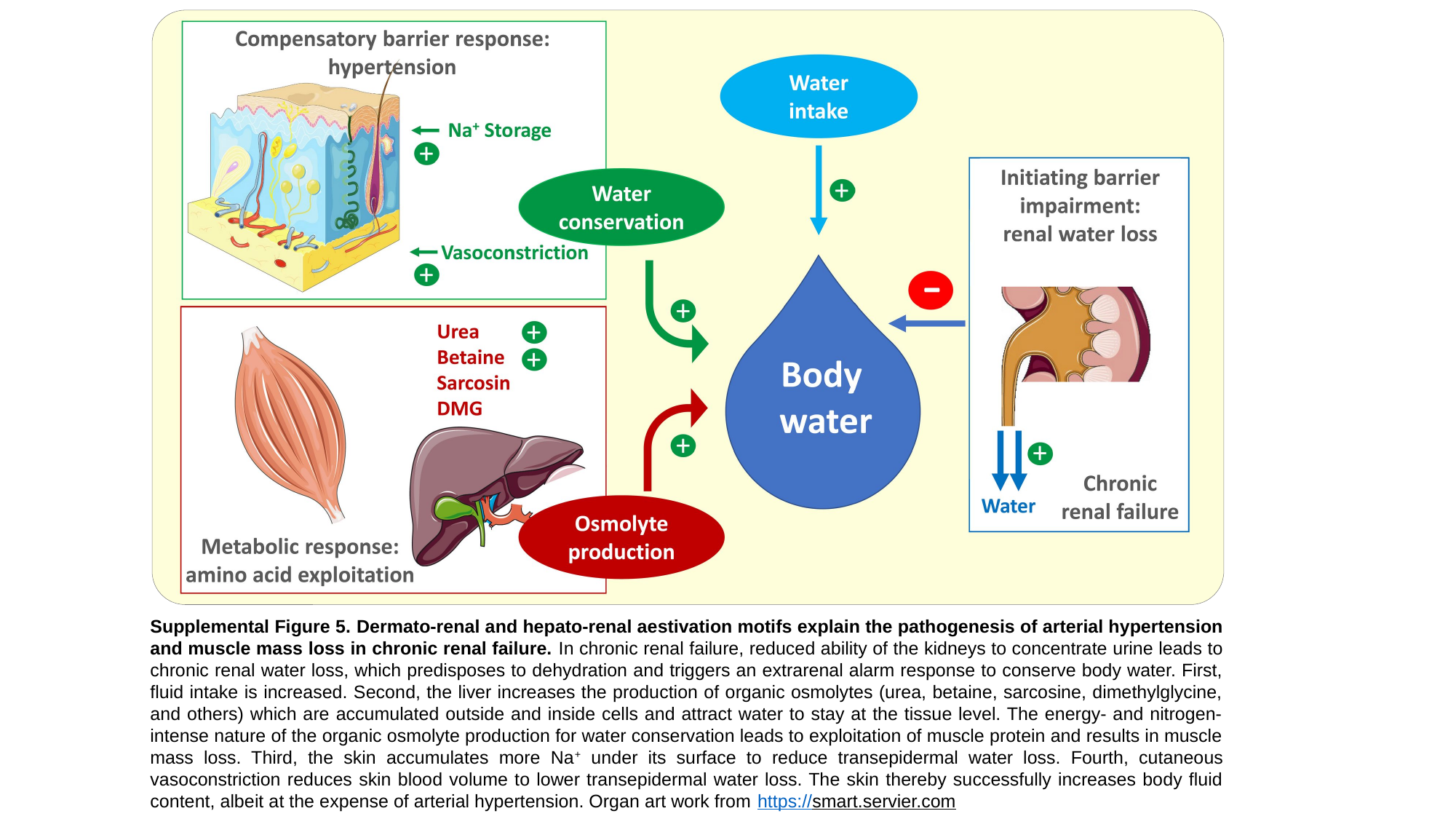

Supplemental Figure 5. Dermato-renal and hepato-renal aestivation motifs explain the pathogenesis of arterial hypertension and muscle mass loss in chronic renal failure. In chronic renal failure, reduced ability of the kidneys to concentrate urine leads to chronic renal water loss, which predisposes to dehydration and triggers an extrarenal alarm response to conserve body water. First, fluid intake is increased. Second, the liver increases the production of organic osmolytes (urea, betaine, sarcosine, dimethylglycine, and others) which are accumulated outside and inside cells and attract water to stay at the tissue level. The energy- and nitrogen-intense nature of the organic osmolyte production for water conservation leads to exploitation of muscle protein and results in muscle mass loss. Third, the skin accumulates more Na+ under its surface to reduce transepidermal water loss. Fourth, cutaneous vasoconstriction reduces skin blood volume to lower transepidermal water loss. The skin thereby successfully increases body fluid content, albeit at the expense of arterial hypertension. Organ art work from https://smart.servier.com

## Slide 6
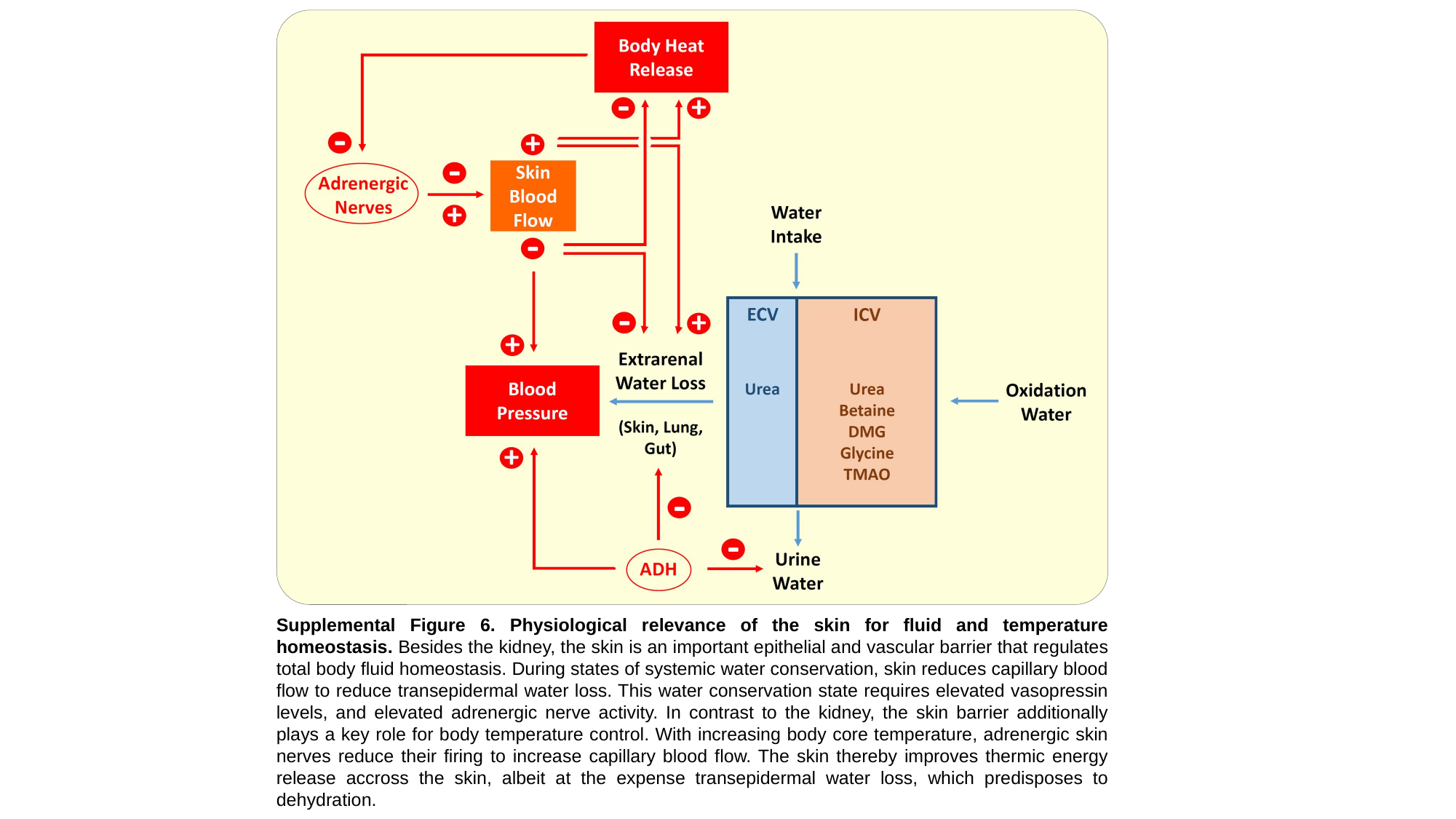

Supplemental Figure 6. Physiological relevance of the skin for fluid and temperature homeostasis. Besides the kidney, the skin is an important epithelial and vascular barrier that regulates total body fluid homeostasis. During states of systemic water conservation, skin reduces capillary blood flow to reduce transepidermal water loss. This water conservation state requires elevated vasopressin levels, and elevated adrenergic nerve activity. In contrast to the kidney, the skin barrier additionally plays a key role for body temperature control. With increasing body core temperature, adrenergic skin nerves reduce their firing to increase capillary blood flow. The skin thereby improves thermic energy release accross the skin, albeit at the expense transepidermal water loss, which predisposes to dehydration.
