# Supplementary material for: Adaptive physiological water conservation explains hypertension and muscle catabolism in experimental chronic renal failure
Source: Acta Physiol (Oxf). 2021 Mar 7;232(1):e13629. doi: 10.1111/apha.13629 (PMC8244025; doi:10.1111/apha.13629)
Supplement: Supplementary file 2 — Table S1 [file APHA-232-e13629-s001.pdf]

Supplemental Table 1: Inventory list of liver and muscle metabolites

Abbreviations used (Figure 3 and 4)	Other abbreviations	Metabolite
1-MeH	1-MH	1-methylhistidine
2-M-BHB-CoA	-	2-methyl-3-hydroxybutyryl CoA
2-MAA-CoA	-	2-methylacetoacetyl CoA
2-MB-CoA	-	2-methylbutyryl CoA
2-MBC	-	2-methylbutyrylcarnitine
2-MC-CoA	-	2-methylcrotoconyl CoA
2-PGA	2-PG, 2PG	2-phosphoglycerate
3-H-2EP	-	3-hydroxy-2-ethylpropionate
3-HP	-	3-hydroxypyruvate
3-MGC	-	3-methylglutarylcarnitine
3-PGA	PGA	3-phosphoglycerate
3-S-L-Ala		3-sulfo-L-alanine
4-HNE-GSH	-	4-hydroxy-nonenal-glutathione
4-HPPA	-	4-hydroxyphenylpyruvic acid
4-I-5-P	-	4-imidazole 5-propionate
6PG	6PG	6-phosphogluconate
Adenylo-Succ	-	adenylosuccinate
ADMA	-	assymetric dimethylarginine
Ala	A	alanine
AMP	-	adenosine monophosphate
Arg	R	arginine
ASA	-	argininosuccinate
Asn	N	asparagine
Asp	D	aspartate
ATP	-	adenosine triphosphate
B-CoA	-	butyryl CoA
BCAR	-	butyrylcarnitine
BGly	-	butyrylglycine
BHB	BOH	beta-hydroxybutyrate
C12	-	laurate
C14	-	myristate (14:0)
C15	-	pentadecanoate
C16	-	palmitate (16:0)
C16-CAR	Pal	palmitoylcarnitine (C16)
C17	-	margarate (17:0)
C18	-	stearate
C18-CAR	-	stearoylcarnitine (C18)
C19	-	nonadecanoate (19:0)
C20	-	arachidate
C20-CAR	-	arachidoylcarnitine (C20)
C22	-	behenate (22:0)
C22-CAR	-	behenoylcarnitine (C22)

C6	-	caproate (6:0)
C8	-	caprylate (8:0)
CAR	-	carnitine
GSH-ss-Cys	-	cystein-glutathione disulfide
Cit	-	citrulline
CP	-	carbamoyl phosphate
Cys	C	cysteine
CysGly	Cys-Gly	cysteinylglycine
DHAP	-	dihydroxyacetone phosphate
DMG	-	dimethylglycine
E4-P	-	erythrose 4-phosphate
EM	-	ethylmalonate
Ery4-P	-	erythritol 4-phosphate
Fi-Asp	-	N-formimino-L-aspartate
FIGLU	-	formiminoglutamate
FBP	F1,6BP	fructose 1,6-bisphosphate
F6-P	Fru-6-P	fructose-6-phosphate
Fru	-	fructose
Fum	-	fumarate
G3P	GA3P, GADP, GAP, TP, GALP, PGAL	glycerol-3-phosphate
Glc	G, Gluc, Glu, etc	glucose
G6-P	G6P	glucose-6-phosphate
Gln	Q	glutamine
Glu	E	glutamate
Gly	G	glycine
GMP	-	guanosine monophosphate
GPC	GroPCho	glycerophosphorylcholine
GSH	-	reduced glutathione
GSSG	-	oxidized glutathione
GSH-ss-Cys	-	cysteine-glutathione disulfide
GAA	Gua	guanidinoacetate
HCO3	-	bicarbonate
HIBA	3-OHIB	3-hydroxyisobutyrate
HIBA-CoA	-	3-hydroxyisobutyryl CoA
His	H	histidine
Hist	H	histamine
HMG-CoA	-	3-hydroxy-3-methylglutaryl CoA
HT	HTU	hypotaurine
HXN	HPX	hypoxanthine
I Ac	-	Imidazolone acetate
I-4 Ac	-	imidazole-4 acetate
IB-CoA	-	isobutyryl CoA
IBCAR	-	isobutyrylcarnitine
Ileu	I	isoleucine
IMP	-	inosine 5'-monophosphate
Inos	I	inosine
IV-CoA	-	isovaleryl CoA

IVC	-	isovalerylcarnitine
IVG	-	isovalerylglycine
KIC	A-KIC, alpha-KIC	alpha-ketoisocaproate or 4-methyl-2-oxopentanoate
KIV	A-KIV, alpha-KIV	alpha-ketoisovalerate or 3-methyl-2-oxobutyrate
KMV	KMVA	keto-β-methylvalerate or 3-methyl-2-oxovalerate
KYN	-	kynurenine
Leu	L	leucine
MA-CoA	-	methylacrylyl CoA
MaHex	-	maltohexaose
Mal	M	malate
Man	-	mannose
MaPent	-	maltopentaose
MaTet	-	maltotetraose
MaTri	-	maltotriose
Met	M	methionine
MG-CoA	-	2-methylglutaconyl CoA
MI	-	myoinositol
MI1-P	-	myoinositol-1-phosphate
MM-CoA	-	methylmalonyl CoA
MS	-	methylsuccinate
NAG	-	N-acetylglutamate
NH4+	-	ammonium
N-MA	-	N-monomethylarginine
NO	-	nitric oxide
OPH	OA	ophthalmate
Orn	-	ornithine
Oa	OAA	oxaloacetate
PEP	-	phosphoenolpyruvate
Phe	F	phenylalanine
PP-CoA	-	propionyl CoA
Pro	P	proline
PRPP	-	phosphoribosylpyrophosphate
Pyr	-	pyruvate
SAH	-	S-adenosylhomocysteine
SAM	SAM-e, AdoMet	S-adenosylmethionine
Sarc	-	sarcosine
SDMA	-	symmetric dimethylarginine
Sed-7P	-	sedoheptulose-7-phosphate
Ser	S	serine
SUCC	Suc	succinate
Tau	Taur	taurine
TG-CoA	-	tiglylCoA
TGCAR	-	tiglylcarnitine
THF	THFA	tetrahydrofolate

THF-[CH2]	-	methylenetetrahydrofolate
Thr	T	threonine
TMA	-	trimethylamine
TMAO	-	trimethylamine-N-oxide
Trp	W	tryptophane
Tyr	Y	tyrosine
UCA	-	urocanate
Val	V	valine
XAN	-	xanthine
XMP	-	xanthosine monophosphate
α-HB	2-HB	2-hydroxybutyrate
α-KB	alpha-KBA, AKB	alpha-ketobutyrate
α-KG	alpha-KGA, AKG	alpha-ketoglutarate
α-KGM	KGM	alpha-ketoglutaramate
β-Ala	bAla	beta-alanine
γ-Glu-AA	-	γ-glutamyl-aminoacids
