# Supplementary material for: Adaptive physiological water conservation explains hypertension and muscle catabolism in experimental chronic renal failure
Source: Acta Physiol (Oxf). 2021 Mar 7;232(1):e13629. doi: 10.1111/apha.13629 (PMC8244025; doi:10.1111/apha.13629)
Supplement: Supplementary file 3 — Table S2 [file APHA-232-e13629-s003.pdf]

Supplemental Table 2

METABOLOMICS FROM LIVER TISSUE

Heat map of statistically significant biochemicals profiled in this study.

Comparison groups significantly different:
- Red: $p \leq 0.05$, group means fold of change ≥ 1.00
- Dark green: $p \leq 0.05$, group means fold of change < 1.00

Comparison groups trending towards significant difference:
- Pink: $0.05 < p < 0.10$, group means fold of change ≥ 1.00
- Light green: $0.05 < p < 0.10$, group means fold of change < 1.00

Pathway Sort Order	Super Pathway	Sub Pathway	Biochemical Name	Platform	Comp ID	KEGG	HMDB	PUBCHEM	Fold of Change, Welch's Two-Sample t-Test: Nephrectomy / Sham	Statistical Values, Welch's Two-Sample t-Test: Nephrectomy / Sham p-value
1		Glycine, Serine and Threonine Metabolism	glycine	LC/MS pos early	58	C00037	HMDB00123	750	1.19	0.0374
2			N-acetylglycine	LC/MS pos early	27710		HMDB00532	10972	1.53	0.0446
4			sarcosine	LC/MS pos early	1516	C00213	HMDB00271	1088	1.35	0.5165
5			dimethylglycine	LC/MS pos early	5086	C01026	HMDB00092	673	1.16	0.3884
6			betaine	LC/MS pos early	3141	C00719	HMDB00043	247	1.40	0.0071
7			betaine aldehyde	LC/MS pos early	15499	C00576	HMDB01252	249	1.28	0.5763
9			serine	LC/MS pos early	1648	C00065	HMDB00187	5951	1.16	0.0158
10			N-acetylserine	LC/MS pos early	37076		HMDB02931	65249	1.50	0.0106
16			threonine	LC/MS pos early	1284	C00188	HMDB00167	6288	1.10	0.2268
17			N-acetylthreonine	LC/MS neg	33939			152204	1.69	0.0021
28		Alanine and Aspartate Metabolism	alanine	LC/MS neg	1126	C00041	HMDB00161	5950	1.18	0.2362
30			N-acetylalanine	LC/MS polar	1585	C02847	HMDB00766	88064	1.68	0.0054
34			aspartate	LC/MS pos early	443	C00049	HMDB00191	5960	1.22	0.0711
35			N-acetylaspartate (NAA)	LC/MS neg	22185	C01042	HMDB00812	65065	5.12	0.0000
38			asparagine	LC/MS pos early	512	C00152	HMDB00168	6267	1.19	0.1295
39			N-acetylasparagine	LC/MS pos early	33942		HMDB06028	99715	1.38	0.1225
41		Glutamate Metabolism	glutamate	LC/MS pos early	57	C00025	HMDB00148	611	0.99	0.9962
42			glutamine	LC/MS pos early	53	C00064	HMDB00641	5961	0.88	0.1358
43			alpha-ketoglutaramate*	LC/MS polar	62101				3.31	0.0002
44			N-acetylglutamate	LC/MS pos early	15720	C00624	HMDB01138	70914	1.77	0.0350
45			N-acetylglutamine	LC/MS pos early	33943	C02716	HMDB06029	182230	1.25	0.6448
47			4-hydroxyglutamate	LC/MS pos early	40499	C03079	HMDB01344	439902	2.50	0.0043
49			gamma-carboxyglutamate	LC/MS pos early	38754		HMDB41900	40772	1.86	0.0000
50			glutamate, gamma-methyl ester	LC/MS pos early	33487		HMDB61715	68662	1.08	0.6900
51			pyroglutamine*	LC/MS pos early	46225			134508	1.55	0.0369
53			beta-citrylglutamate	LC/MS neg	54923	C20775		72715786	1.26	0.2388
55			carboxyethyl-GABA	LC/MS pos early	40007		HMDB02201	2572	2.43	0.0001
56			N-methyl-GABA	LC/MS pos early	39577	C15987		70703	1.67	0.0000
59			S-1-pyrroline-5-carboxylate	LC/MS pos early	42370	C04322	HMDB01301	1196	1.52	0.0105
65		Histidine Metabolism	histidine	LC/MS neg	59	C00135	HMDB00177	6274	1.24	0.0042
66			1-methylhistidine	LC/MS pos early	30460	C01152	HMDB00001	92105	4.51	0.0001
67			3-methylhistidine	LC/MS pos early	15677	C01152	HMDB00479	64969	2.43	0.0000
68			N-acetylhistidine	LC/MS pos early	33946	C02997	HMDB32055	75619	0.99	0.7556
69			N-acetyl-3-methylhistidine*	LC/MS pos early	43256			193270	2.76	0.0000
70			N-acetyl-1-methylhistidine*	LC/MS pos early	43255			193270	3.75	0.0000
74			imidazole propionate	LC/MS pos early	40730		HMDB02271	70630	2.64	0.0006
75			formiminoglutamate	LC/MS pos early	43493	C00439	HMDB00854	439233	0.55	0.3057
76			imidazole lactate	LC/MS pos early	15716	C05568	HMDB02320	440129	4.36	0.0000
79			N-acetylcarnosine	LC/MS neg	43488		HMDB12881	9903482	2.97	0.0086
80			anserine	LC/MS pos early	15747	C01262	HMDB00194	112072	1.04	0.8476
81			histamine	LC/MS pos early	1574	C00388	HMDB00870	774	0.98	0.5507
83			1-methyl-4-imidazoleacetate	LC/MS pos early	32350	C05828	HMDB02820	75810	3.31	0.0001
84			1-ribosyl-imidazoleacetate*	LC/MS pos early	61868	C05131	HMDB02331	5117448	3.48	0.0000
85			4-imidazoleacetate	LC/MS pos early	32349	C02835	HMDB02024	96215	1.52	0.0268
89			N-acetylhistamine	LC/MS pos early	48679	C05135	HMDB13253	69602	1.10	0.8829
91			lysine	LC/MS pos early	1301	C00047	HMDB00182	5962	1.14	0.0939

92			N2-acetyllysine	LC/MS polar	36751	C12989	HMDB00446	92907	1.22	0.0520
93			N6-acetyllysine	LC/MS neg	36752	C02727	HMDB00206	92832	2.09	0.0015
98			N6,N6,N6-trimethyllysine	LC/MS pos early	1498	C03793	HMDB01325	440120	1.14	0.1377
100			5-(galactosylhydroxy)-L-lysine	LC/MS pos early	43582				1.58	0.0000
101		Lysine Metabolism	saccharopine	LC/MS pos early	1495	C00449	HMDB00279	160556	0.88	0.6612
102			2-aminoadipate	LC/MS polar	6146	C00956	HMDB00510	469	1.14	0.5748
106			glutarylcarnitine (C5-DC)	LC/MS pos early	44664		HMDB13130	71464488	1.87	0.0056
110			pipecolate	LC/MS pos early	1444	C00408	HMDB00070	849	2.49	0.0000
114			5-aminovalerate	LC/MS neg	18319	C00431	HMDB03355	138	1.56	0.0003
115			N,N,N-trimethyl-5-aminovalerate	LC/MS pos early	57687				1.59	0.0293
116			phenylalanine	LC/MS pos early	64	C00079	HMDB00159	6140	1.21	0.0150
117		Phenylalanine Metabolism	N-acetylphenylalanine	LC/MS neg	33950	C03519	HMDB00512	74839	1.86	0.0350
118			1-carboxyethylphenylalanine	LC/MS neg	62566				1.82	0.0261
122			phenyllactate (PLA)	LC/MS neg	22130	C05607	HMDB00779	3848	2.25	0.0027
132			tyrosine	LC/MS pos early	1299	C00082	HMDB00158	6057	1.13	0.1261
133			N-acetyltyrosine	LC/MS neg	32390		HMDB00866	68310	1.48	0.0441
134			1-carboxyethyltyrosine	LC/MS neg	62564				1.64	0.0454
142			4-hydroxyphenylpyruvate	LC/MS neg	1669	C01179	HMDB00707	979	0.72	0.0670
145		Tyrosine Metabolism	3-(4-hydroxyphenyl)lactate	LC/MS neg	32197	C03672	HMDB00755	9378	9.77	0.0000
149			phenol sulfate	LC/MS neg	32553	C02180	HMDB60015	74426	5.16	0.0000
163			3-methoxytyrosine	LC/MS pos early	12017		HMDB01434	1670	0.83	0.0496
185			p-cresol glucuronide*	LC/MS neg	48841		HMDB11686	154035	1.26	0.3766
187			N-formylphenylalanine	LC/MS neg	48433			759256	0.93	0.7675
207			tryptophan	LC/MS pos early	54	C00078	HMDB00929	6305	1.22	0.0008
214			C-glycosyltryptophan	LC/MS pos early	48782			10981970	1.34	0.0015
222			kynurenate	LC/MS neg	1417	C01717	HMDB00715	3845	4.35	0.0037
225			anthranilate	LC/MS pos early	57746	C00108	HMDB01123	227	1.24	0.2608
230		Tryptophan Metabolism	picolinate	LC/MS pos early	1512	C10164	HMDB02243	1018	2.61	0.0014
241			indolepropionate	LC/MS neg	32405		HMDB02302	3744	0.54	0.0141
246	Amino Acid		indole-3-carboxylate	LC/MS neg	38116	C19837	HMDB03320	69867	0.91	0.4037
248			indoleacetylglycine	LC/MS neg	54730			446640	3.13	0.0001
251			3-indoxyl sulfate	LC/MS neg	27672		HMDB00682	10258	5.97	0.0000
257			leucine	LC/MS pos early	60			5246661	1.11	0.1365
258			N-acetylleucine	LC/MS neg	1587	C02710	HMDB11756	70912	0.95	0.8114
259			1-carboxyethylleucine	LC/MS neg	62559				1.51	0.0557
268			isovalerylglycine	LC/MS neg	35107		HMDB00678	546304	1.79	0.0308
269			isovalerylcarnitine (C5)	LC/MS pos early	34407		HMDB00688	6426851	1.07	0.8867
276			beta-hydroxyisovalerate	LC/MS polar	12129		HMDB00754	69362	1.12	0.2359
283			3-methylglutarylcarnitine (2)	LC/MS pos early	46548		HMDB00552	128145	3.09	0.0020
292			isoleucine	LC/MS pos early	1125	C00407	HMDB00172	6306	1.10	0.1258
294			N-acetylisoleucine	LC/MS neg	33967		HMDB61684	2802421	1.36	0.0555
295		Leucine, Isoleucine and Valine Metabolism	1-carboxyethylisoleucine	LC/MS neg	62558				1.92	0.0147
297			alpha-hydroxyisovalerate	LC/MS polar	46537		HMDB00407	99823	11.30	0.0000
299			2-methylbutyrylcarnitine (C5)	LC/MS pos early	45095		HMDB00378	6426901	1.54	0.2068
307			ethylmalonate	LC/MS polar	15765		HMDB00622	11756	1.40	0.0617
308			methylsuccinate	LC/MS polar	15745		HMDB01844	10349	2.10	0.0017
314			valine	LC/MS pos early	1649	C00183	HMDB00883	6287	1.09	0.1650
315			N-acetylvaline	LC/MS neg	1591		HMDB11757	66789	1.07	0.5525
316			1-carboxyethylvaline	LC/MS neg	62562				1.76	0.0579
321			isobutyrylcarnitine (C4)	LC/MS pos early	33441		HMDB00736	168379	1.74	0.1664
323			3-hydroxyisobutyrate	LC/MS polar	1549	C06001	HMDB00336	87	1.57	0.0430
327			methionine	LC/MS neg	1302	C00073	HMDB00696	6137	1.16	0.0048
328			N-acetylmethionine	LC/MS neg	1589	C02712	HMDB11745	448580	1.97	0.0118
329			N-formylmethionine	LC/MS neg	2829	C03145	HMDB01015	439750	1.42	0.1645
331			methionine sulfone	LC/MS pos early	44878			69961	7.10	0.0000
332			methionine sulfoxide	LC/MS pos early	18374	C02989	HMDB02005	158980	1.14	0.2272
338			S-adenosylmethionine (SAM)	LC/MS pos early	15915	C00019	HMDB01185	34756	1.08	0.6670
339			S-adenosylhomocysteine (SAH)	LC/MS neg	42382	C00021	HMDB00939	439155	1.04	0.6917
343			cystathionine	LC/MS pos early	15705	C02291	HMDB00099	439258	1.16	0.4146

345		Methionine, Cysteine, SAM and Taurine Metabolism	cysteine	LC/MS pos early	1868	C00097	HMDB00574	5862	1.99	0.0092
346			N-acetylcysteine	LC/MS pos early	1586	C06809	HMDB01890	12035	2.14	0.0000
347			S-methylcysteine	LC/MS pos early	39592		HMDB02108	24417	0.90	0.3403
356			cysteine sulfinic acid	LC/MS pos early	37443	C00606	HMDB00996	109	2.63	0.0128
357			hypotaurine	LC/MS pos early	590	C00519	HMDB00965	107812	0.83	0.4770
358			taurine	LC/MS neg	2125	C00245	HMDB00251	1123	0.35	0.0001
359			N-acetyltaurine	LC/MS neg	48187			159864	0.40	0.0004
361			taurocyamine	LC/MS pos early	35117	C01959	HMDB03584	68340	1.39	0.0844
363			3-sulfo-L-alanine	LC/MS polar	47089	C00506	HMDB02757	72886	4.77	0.0000
366			argininosuccinate	LC/MS pos early	15497	C03406	HMDB00052	828	2.23	0.0878
367			urea	LC/MS pos early	1670	C00086	HMDB00294	1176	2.83	0.0000
369			ornithine	LC/MS pos early	1493	C00077	HMDB03374	6262	1.19	0.0582
373			citrulline	LC/MS pos early	2132	C00327	HMDB00904	9750	1.68	0.0040
374			homoarginine	LC/MS pos early	22137	C01924	HMDB00670	9085	1.05	0.4930
375			homocitrulline	LC/MS polar	22138	C02427	HMDB00679	65072	3.34	0.0000
376			proline	LC/MS pos early	1898	C00148	HMDB00162	145742	1.11	0.1775
379			dimethylarginine (SDMA + ADMA)	LC/MS pos early	36808	C03626	HMDB01539	123831	1.62	0.0012
380			N-acetylarginine	LC/MS pos early	33953	C02562	HMDB04620	67427	1.61	0.0103
381		Urea cycle; Arginine and Proline Metabolism	N-acetylcitrulline	LC/MS neg	48434	C15532	HMDB00856	656979	1.57	0.1188
383			N-delta-acetylornithine	LC/MS polar	43249			9920500	1.67	0.0000
384			N-alpha-acetylornithine	LC/MS pos early	32984	C00437	HMDB03357	439232	1.89	0.0125
385			N2,N5-diacetylornithine	LC/MS neg	43591			10398396	1.19	0.1794
388			trans-4-hydroxyproline	LC/MS pos early	32306	C01157	HMDB00725	5810	1.81	0.0001
390			pro-hydroxy-pro	LC/MS pos early	35127		HMDB06695	11673055	3.03	0.0014
393			N-methylproline	LC/MS pos early	37431			557	1.39	0.2193
395			N-monomethylarginine	LC/MS pos early	43586	C03884	HMDB29416	132862	1.39	0.0245
396			argininate*	LC/MS pos early	57461		HMDB03148	160437	3.06	0.0000
399			guanidinoacetate	LC/MS pos early	43802	C00581	HMDB00128	763	1.60	0.0813
400		Creatine Metabolism	creatine	LC/MS pos early	27718	C00300	HMDB00064	586	0.69	0.0051
401			creatinine	LC/MS pos early	513	C00791	HMDB00562	588	2.84	0.0000
409			N-acetyl-isoputreanine*	LC/MS pos early	62309				3.20	0.0006
410		Polyamine Metabolism	spermidine	LC/MS pos early	485	C00315	HMDB01257	1102	1.21	0.0348
414			acisoga	LC/MS pos early	43258			129397	2.37	0.0003
418			5-methylthioadenosine (MTA)	LC/MS pos early	1419	C00170	HMDB01173	439176	1.28	0.1463
426			1-methylguanidine	LC/MS pos early	48114	C02294	HMDB01522	10111	2.24	0.0031
427		Guanidino and Acetamido Metabolism	4-guanidinobutanoate	LC/MS pos early	15681	C01035	HMDB03464	500	4.30	0.0000
428			guanidinosuccinate	LC/MS neg	32446	C03139	HMDB03157	97856	3.60	0.0009
430			glutathione, reduced (GSH)	LC/MS pos early	2127	C00051	HMDB00125	124886	0.66	0.1330
431			glutathione, oxidized (GSSG)	LC/MS pos early	27727	C00127	HMDB03337	65359	1.04	0.5987
433			cysteine-glutathione disulfide	LC/MS pos early	35159		HMDB00656	4247235	2.50	0.0491
434			S-methylglutathione	LC/MS pos early	33944	C11347		3605667	0.43	0.0046
435		Glutathione Metabolism	S-lactoylglutathione	LC/MS neg	15731	C03451	HMDB01066	440018	0.67	0.2861
436			cysteinylglycine	LC/MS pos early	35637	C01419	HMDB00078	439498	0.86	0.4951
439			5-oxoproline	LC/MS neg	1494	C01879	HMDB00267	7405	1.26	0.1244
442			2-hydroxybutyrate/2-hydroxyisobutyrate	LC/MS polar	52281				1.90	0.0259
444			ophthalmate	LC/MS pos early	34592		HMDB05765	7018721	1.21	0.3896
447			4-hydroxy-nonenal-glutathione	LC/MS neg	48487				0.45	0.0120
451			gamma-glutamylalanine	LC/MS pos early	37063		HMDB29142	440103	4.28	0.0051
453			gamma-glutamylglutamate	LC/MS neg	36738	C05282	HMDB11737	92865	2.21	0.0004
454			gamma-glutamylglutamine	LC/MS pos early	2730	C05283	HMDB11738	150914	2.27	0.0452
455			gamma-glutamylglycine	LC/MS pos early	33949		HMDB11667	165527	2.66	0.0272
456			gamma-glutamylhistidine	LC/MS pos early	18245			7017195	1.96	0.0331
457			gamma-glutamylisoleucine*	LC/MS pos early	34456		HMDB11170	14253342	1.46	0.0137
458			gamma-glutamylleucine	LC/MS pos early	18369		HMDB11171	151023	1.50	0.0175
459		Gamma-glutamyl Amino Acid	gamma-glutamyl-alpha-lysine	LC/MS pos early	55015			65254	2.50	0.0254
460			gamma-glutamyl-epsilon-lysine	LC/MS pos early	33934		HMDB03869	7015685	1.46	0.0003
461			gamma-glutamylmethionine	LC/MS pos early	44872		HMDB29155	7009567	2.24	0.0012
462			gamma-glutamylphenylalanine	LC/MS pos early	33422		HMDB00594	111299	1.96	0.0252
463	Peptide		gamma-glutamylthreonine	LC/MS pos early	33364		HMDB29159	76078708	2.33	0.0051

465	Peptide		gamma-glutamyltyrosine	LC/MS pos early	2734		HMDB11741	94340	2.58	0.0272
466			gamma-glutamylvaline	LC/MS pos early	43829		HMDB11172	7015683	1.39	0.0357
469			gamma-glutamyl-2-aminobutyrate	LC/MS pos early	37092				1.22	0.3539
611			glycylisoleucine	LC/MS pos early	36659		HMDB28844	88079	1.21	0.2465
612			glycylleucine	LC/MS pos early	34398	C02155	HMDB00759	92843	1.64	0.0009
622			glycylvaline	LC/MS pos early	18357		HMDB28854	97417	1.41	0.0031
646		Dipeptide	isoleucylglycine	LC/MS neg	40008		HMDB28907	342532	1.83	0.0088
664			leucylglycine	LC/MS pos early	40045		HMDB28929	79070	1.73	0.0138
715			phenylalanylglycine	LC/MS pos early	41370		HMDB28995	98207	2.17	0.0022
734			prolylglycine	LC/MS pos early	40703		HMDB11178	6426709	1.48	0.0031
815			valylglycine	LC/MS neg	40475		HMDB29127	136487	1.76	0.0185
897		Acetylated Peptides	phenylacetylglycine	LC/MS neg	33945	C05598	HMDB00821	68144	2.39	0.0047
922			1,5-anhydroglucitol (1,5-AG)	LC/MS neg	20675	C07326	HMDB02712	64960	1.22	0.0909
925			glucose	LC/MS polar	20488	C00031	HMDB00122	79025	0.81	0.0039
926			glucose 6-phosphate	LC/MS polar	31260	C00668	HMDB01401	5958	0.97	0.9707
932			fructose 1,6-diphosphate/glucose 1,6-diphosphate/myo-i	LC/MS neg	46896	C00354			1.60	0.2113
934			dihydroxyacetone phosphate (DHAP)	LC/MS pos early	15522	C00111	HMDB01473	668	2.18	0.0887
938		Glycolysis, Gluconeogenesis, and Pyruvate Metabolism	2-phosphoglycerate	LC/MS polar	35629	C00631	HMDB03391	59	1.54	0.0987
939			3-phosphoglycerate	LC/MS neg	1414	C00597	HMDB00807	724	1.11	0.2612
940			phosphoenolpyruvate (PEP)	LC/MS neg	597	C00074	HMDB00263	1005	1.08	0.3838
941			pyruvate	LC/MS neg	42582	C00022	HMDB00243	1060	0.81	0.2457
942			lactate	LC/MS neg	527	C00186	HMDB00190	612	1.30	0.1276
945			glycerate	LC/MS polar	1572	C00258	HMDB00139	752	1.08	0.4699
948			6-phosphogluconate	LC/MS neg	15442	C00345	HMDB01316	91493	1.50	0.0785
951		Pentose Phosphate Pathway	ribose 1-phosphate	LC/MS polar	1763	C00620	HMDB01489	439236	0.77	0.0444
953			sedoheptulose-7-phosphate	LC/MS neg	35649	C05382	HMDB01068	616	0.33	0.0243
959			ribose	LC/MS polar	1471	C00121	HMDB00283	5779	0.91	0.3723
960			ribitol	LC/MS polar	15772	C00474	HMDB00508	6912	0.80	0.0385
961			ribonate	LC/MS polar	27731	C01685	HMDB00867	5460677	0.70	0.0160
982		Pentose Metabolism	arabitol/xylitol	LC/MS polar	48885	C01904		6912	1.10	0.2808
983			ribulose/xylulose	LC/MS polar	48340			5289590	0.82	0.4070
984			arabonate/xylonate	LC/MS polar	48255				1.26	0.0042
985	Carbohydrate		sedoheptulose	LC/MS polar	53237		HMDB03219	5459879	0.60	0.0001
986			ribulonate/xylulonate*	LC/MS polar	61858				0.72	0.0009
987			maltohexaose	LC/MS pos early	35170	C01936	HMDB12253	439606	0.56	0.0384
989			maltopentaose	LC/MS polar	35163	C06218	HMDB12254	13489094	0.30	0.0004
990		Glycogen Metabolism	maltotetraose	LC/MS polar	15910	C02052	HMDB01296	446495	0.35	0.0036
992			maltotriose	LC/MS polar	44688	C01835	HMDB01262	439586	0.48	0.0153
995			maltose	LC/MS polar	15586	C00208	HMDB00163	10991489	0.62	0.0225
1035			fructose	LC/MS polar	48195	C00095	HMDB00660	5984	0.59	0.0008
1040			mannitol/sorbitol	LC/MS polar	46142	C00794	HMDB00247	5780	0.56	0.0005
1041		Fructose, Mannose and Galactose Metabolism	mannose	LC/MS polar	584	C00159	HMDB00169	18950	0.79	0.0071
1052			galactose 1-phosphate	LC/MS polar	15706	C00446	HMDB00645	123912	1.16	0.2459
1060			galactonate	LC/MS polar	27719	C00880	HMDB00565	128869	2.09	0.2457
1077		Nucleotide Sugar	cytidine 5'-monophospho-N-acetylneuraminic acid	LC/MS polar	36831	C00128	HMDB01176	448209	1.15	0.2112
1088			glucuronate	LC/MS polar	15443	C00191	HMDB00127	444791	0.92	0.3792
1092			N-acetylglucosamine 6-phosphate	LC/MS polar	15107	C00357	HMDB02817	439219	1.29	0.0136
1093			N-acetyl-glucosamine 1-phosphate	LC/MS polar	15741	C04256	HMDB01367	440364	1.38	0.0100
1099		Aminosugar Metabolism	N-acetylneuraminate	LC/MS polar	32377	C00270	HMDB00230	439197	1.04	0.7842
1110			N-acetylglucosaminylasparagine	LC/MS pos early	48149	C04540	HMDB00489	123826	0.78	0.0268
1111			erythronate*	LC/MS polar	42420		HMDB00613	2781043	1.47	0.0000
1113			N-acetylglucosamine/N-acetylgalactosamine	LC/MS polar	46539		HMDB00215	24139	1.19	0.1328
1114		Advanced Glycation End-product	N6-carboxymethyllysine	LC/MS pos early	36713			123800	2.11	0.0001
1118			citrate	LC/MS neg	1564	C00158	HMDB00094	311	1.85	0.1601
1120			aconitate [cis or trans]	LC/MS neg	46173				8.96	0.0000
1124			alpha-ketoglutarate	LC/MS polar	528	C00026	HMDB00208	51	0.84	0.5680
1126			succinylcarnitine (C4-DC)	LC/MS pos early	37058		HMDB61717	71464481	1.55	0.1486
1127		TCA Cycle	succinate	LC/MS neg	1437	C00042	HMDB00254	1110	0.89	0.3137
1128	Energy		fumarate	LC/MS polar	1643	C00122	HMDB00134	444972	1.22	0.0517

1129			malate	LC/MS neg	1303	C00149	HMDB00156	525	1.33	0.0038
1134			tricarballylate	LC/MS neg	15729	C19806	HMDB31193	14925	5.27	0.0000
1139			2-methylcitrate/homocitrate	LC/MS neg	52282				2.43	0.0000
1141		Oxidative Phosphorylation	acetylphosphate	LC/MS polar	15488	C00227	HMDB01494	186	0.86	0.0173
1143			phosphate	LC/MS neg	42109	C00009	HMDB01429	1061	1.03	0.5504
1145		Fatty Acid Synthesis	malonylcarnitine	LC/MS pos early	37059		HMDB02095	22833583	1.38	0.0058
1161		Medium Chain Fatty Acid	caproate (6:0)	LC/MS neg	32489	C01585	HMDB00535	8892	0.74	0.0299
1162			heptanoate (7:0)	LC/MS neg	1644	C17714	HMDB00666	8094	0.74	0.1387
1163			caprylate (8:0)	LC/MS neg	32492	C06423	HMDB00482	379	0.90	0.9255
1169			5-dodecenoate (12:1n7)	LC/MS neg	33968		HMDB00529	5312378	0.79	0.8199
1171		Long Chain Fatty Acid	myristate (14:0)	LC/MS neg	1365	C06424	HMDB00806	11005	0.59	0.0058
1172			myristoleate (14:1n5)	LC/MS neg	32418	C08322	HMDB02000	5281119	0.83	0.2396
1175			pentadecanoate (15:0)	LC/MS neg	1361	C16537	HMDB00826	13849	0.73	0.0079
1176			palmitate (16:0)	LC/MS neg	1336	C00249	HMDB00220	985	0.86	0.0928
1177			palmitoleate (16:1n7)	LC/MS neg	33447	C08362	HMDB03229	445638	0.45	0.0004
1179			margarate (17:0)	LC/MS neg	1121		HMDB02259	10465	1.03	0.9369
1180			10-heptadecenoate (17:1n7)	LC/MS neg	33971		HMDB60038	5312435	0.49	0.0010
1184			oleate/vaccenate (18:1)	LC/MS neg	52285				0.82	0.0854
1191			nonadecanoate (19:0)	LC/MS neg	1356	C16535	HMDB00772	12591	1.33	0.0246
1192			10-nonadecenoate (19:1n9)	LC/MS neg	33972		HMDB13622	5312513	0.60	0.0053
1198			eicosenoate (20:1)	LC/MS neg	33587	C16526	HMDB02231	5282768	0.83	0.1965
1202			behenate (22:0)*	LC/MS neg	12125	C08281	HMDB00944	8215	1.65	0.0110
1203			erucate (22:1n9)	LC/MS neg	1552	C08316	HMDB02068	5281116	1.04	0.8744
1210		Polyunsaturated Fatty Acid (n3 and n6)	heneicosapentaenoate (21:5n3)	LC/MS neg	57658			11998573	0.65	0.0481
1211			hexadecadienoate (16:2n6)	LC/MS neg	57652		HMDB00477		1.02	0.9748
1215			stearidonate (18:4n3)	LC/MS neg	33969	C16300	HMDB06547	5312508	1.24	0.3361
1216			eicosapentaenoate (EPA; 20:5n3)	LC/MS neg	18467	C06428	HMDB01999	446284	1.13	0.6405
1217			docosapentaenoate (n3 DPA; 22:5n3)	LC/MS neg	32504	C16513	HMDB06528	6441454	0.79	0.1623
1218			docosahexaenoate (DHA; 22:6n3)	LC/MS neg	44675	C06429	HMDB02183	445580	0.92	0.5362
1222			nisinate (24:6n3)	LC/MS neg	57810		HMDB02007	11792612	0.70	0.0381
1223			linoleate (18:2n6)	LC/MS neg	1105	C01595	HMDB00673	5280450	0.98	0.7867
1225			linolenate [alpha or gamma; (18:3n3 or 6)]	LC/MS neg	34035	C06426	HMDB03073	5280934	0.98	0.8665
1227			dihomo-linolenate (20:3n3 or n6)	LC/MS neg	35718	C03242	HMDB02925	5280581	0.78	0.0567
1228			arachidonate (20:4n6)	LC/MS neg	1110	C00219	HMDB01043	444899	1.36	0.0225
1229			adrenate (22:4n6)	LC/MS neg	32980	C16527	HMDB02226	5497181	1.00	0.7884
1230			docosapentaenoate (n6 DPA; 22:5n6)	LC/MS neg	37478	C16513	HMDB01976	6441454	0.88	0.4523
1231			docosadienoate (22:2n6)	LC/MS neg	32415	C16533	HMDB61714	5282807	0.91	0.4837
1232			dihomo-linoleate (20:2n6)	LC/MS neg	17805	C16525	HMDB05060	6439848	0.64	0.0171
1234			mead acid (20:3n9)	LC/MS neg	35174		HMDB10378	5312531	1.08	0.8754
1289		Fatty Acid, Branched	15-methylpalmitate (i17:0)	LC/MS neg	38768			17903417	0.73	0.0513
1292			17-methylstearate (i19:0)	LC/MS neg	38296		HMDB37397	3083779	1.03	0.9716
1303		Fatty Acid, Dicarboxylate	glutarate (C5-DC)	LC/MS polar	396	C00489	HMDB00661	743	1.14	0.5738
1306			3-methylglutarate/2-methylglutarate	LC/MS polar	54676		HMDB00752		1.64	0.0085
1309			adipate (C6-DC)	LC/MS polar	21134	C06104	HMDB00448	196	1.84	0.0493
1311			2-hydroxyadipate	LC/MS polar	31934	C02360	HMDB00321	193530	1.08	0.7484
1313			3-methyladipate	LC/MS polar	36749		HMDB00555	12292	1.65	0.0288
1315			maleate	LC/MS polar	20676	C01384	HMDB00176	444266	1.31	0.0937
1316			pimelate (C7-DC)	LC/MS polar	15704	C02656	HMDB00857	385	2.13	0.1034
1317			heptenedioate (C7:1-DC)*	LC/MS polar	61866				1.56	0.0284
1322			azelate (C9-DC)	LC/MS neg	18362	C08261	HMDB00784	2266	0.78	0.1795
1324			sebacate (C10-DC)	LC/MS neg	32398	C08277	HMDB00792	5192	1.08	0.4678
1325			undecanedioate (C11-DC)	LC/MS neg	42395		HMDB00888	15816	0.69	0.0336
1326			dodecadienoate (12:2)*	LC/MS neg	62276				0.83	0.0388
1328			dodecanedioate (C12-DC)	LC/MS neg	32388	C02678	HMDB00623	12736	1.11	0.6243
1329			tetradecanedioate (C14-DC)	LC/MS neg	35669		HMDB00872	13185	1.12	0.8430
1330			hexadecanedioate (C16-DC)	LC/MS neg	35678	C19615	HMDB00672	10459	1.10	0.6051
1332			hexadecenedioate (C16:1-DC)*	LC/MS neg	61862				0.85	0.2916
1334			octadecenedioate (C18:1-DC)*	LC/MS neg	61861				1.22	0.0983
1335			octadecadienedioate (C18:2-DC)*	LC/MS neg	61860				1.06	0.9742

1382	Fatty Acid, Amino	2-aminoheptanoate	LC/MS pos early	43761			227939	0.64	0.0099
1398	Fatty Acid Metabolism (also BCAA Metabolism)	butyrylcarnitine (C4)	LC/MS pos early	32412	C02862	HMDB02013	439829	0.99	0.7301
1399		butyrylglycine	LC/MS neg	31850		HMDB00808	88412	2.86	0.0301
1401		propionylcarnitine (C3)	LC/MS pos early	32452	C03017	HMDB00824	107738	0.94	0.5840
1402		propionylglycine	LC/MS polar	31932		HMDB00783	98681	1.23	0.3343
1404		methylmalonate (MMA)	LC/MS polar	1496	C02170	HMDB00202	487	1.08	0.8784
1414	Fatty Acid Metabolism(Acyl Glycine)	hexanoylglycine	LC/MS neg	35436		HMDB00701	99463	1.49	0.1331
1419		N-palmitoylglycine	LC/MS neg	42092		HMDB13034	151008	0.80	0.1498
1428	Fatty Acid Metabolism(Acyl Carnitine)	acetylcarnitine (C2)	LC/MS pos early	32198	C02571	HMDB00201	1	0.62	0.0020
1430		3-hydroxybutyrylcarnitine (1)	LC/MS pos early	43264		HMDB13127	53481617	2.31	0.0111
1431		3-hydroxybutyrylcarnitine (2)	LC/MS pos early	52984		HMDB13127		1.84	0.0090
1436		octanoylcarnitine (C8)	LC/MS pos late	33936	C02838	HMDB00791	123701	1.25	0.3055
1443		myristoylcarnitine (C14)	LC/MS pos late	33952		HMDB05066	6426854	0.74	0.2613
1445		palmitoylcarnitine (C16)	LC/MS pos late	44681	C02990	HMDB00222	461	0.77	0.1329
1446		palmitoleoylcarnitine (C16:1)*	LC/MS pos late	53223			71464547	0.67	0.1068
1447		stearoylcarnitine (C18)	LC/MS pos late	34409		HMDB00848	6426855	1.04	0.8882
1448		linoleoylcarnitine (C18:2)*	LC/MS pos late	46223		HMDB06469	6450015	1.33	0.0887
1450		3-hydroxyoleoylcarnitine	LC/MS pos late	61840				0.60	0.0268
1451		oleoylcarnitine (C18:1)	LC/MS pos late	35160		HMDB05065	6441392	0.74	0.1587
1456		adipoylcarnitine (C6-DC)	LC/MS pos early	52988		HMDB61677	71296139	4.86	0.0001
1459		pimeloylcarnitine/3-methyladipoylcarnitine (C7-DC)	LC/MS pos early	53224				1.10	0.6465
1463		arachidoylcarnitine (C20)*	LC/MS pos late	57513		HMDB06460		3.51	0.0162
1464		arachidonoylcarnitine (C20:4)	LC/MS pos late	57518				1.34	0.1119
1466		behenoylcarnitine (C22)*	LC/MS pos late	57514				1.83	0.0072
1468		dihomo-linolenoylcarnitine (C20:3n3 or 6)*	LC/MS pos late	57521				0.70	0.0057
1469		dihomo-linoleoylcarnitine (C20:2)*	LC/MS pos late	57520				0.85	0.5508
1470		eicosenoylcarnitine (C20:1)*	LC/MS pos late	57519				1.44	0.3072
1471		erucoylcarnitine (C22:1)*	LC/MS pos late	57525				2.00	0.0878
1475		docosahexaenoylcarnitine (C22:6)*	LC/MS pos late	57523				1.75	0.0025
1477		lignoceroylcarnitine (C24)*	LC/MS pos late	57515				1.99	0.0036
1478		margaroylcarnitine (C17)*	LC/MS pos late	57512		HMDB06210		1.32	0.3544
1479		nervonoylcarnitine (C24:1)*	LC/MS pos late	57531				1.86	0.0226
1481		ximenoylcarnitine (C26:1)*	LC/MS pos late	57517				2.13	0.0021
1485		3-hydroxypalmitoylcarnitine	LC/MS pos late	61839				0.60	0.0308
1487	Carnitine Metabolism	deoxycarnitine	LC/MS pos early	36747	C01181	HMDB01161	134	0.98	0.6679
1488		carnitine	LC/MS pos early	15500	C00318	HMDB00062	10917	1.08	0.4390
1493	Ketone Bodies	3-hydroxybutyrate (BHBA)	LC/MS polar	542	C01089	HMDB00357	441	0.94	0.6050
1496	Fatty Acid Metabolism (Acyl Choline)	palmitoylcholine	LC/MS pos late	52944			151731	2.97	0.0422
1497		oleoylcholine	LC/MS pos late	53260				2.42	0.0957
1498		palmitoleylcholine	LC/MS pos late	53257				2.11	0.0765
1500		linoleoylcholine*	LC/MS pos late	57463				2.26	0.0686
1502		stearoylcholine*	LC/MS pos late	57464				6.13	0.0011
1504		arachidonoylcholine	LC/MS pos late	53261				2.29	0.0076
1511	Fatty Acid, Monohydroxy	2-hydroxyheptanoate*	LC/MS polar	61827			2750949	1.24	0.1603
1516		2-hydroxypalmitate	LC/MS neg	35675		HMDB31057	92836	1.05	0.6593
1518		2-hydroxystearate	LC/MS neg	17945	C03045		69417	1.07	0.3303
1525		3-hydroxyhexanoate	LC/MS neg	53230			151492	1.35	0.2452
1526		3-hydroxyoctanoate	LC/MS neg	22001		HMDB01954	26613	1.04	0.6758
1528		3-hydroxydecanoate	LC/MS neg	22053		HMDB02203	26612	1.10	0.6784
1530		3-hydroxylaurate	LC/MS neg	32457		HMDB00387	94216	1.05	0.9894
1545		16-hydroxypalmitate	LC/MS neg	39609	C18218	HMDB06294	10466	1.09	0.5126
1549		13-HODE + 9-HODE	LC/MS neg	37752			43013	1.01	0.8363
1570	Fatty Acid, Dihydroxy	12,13-DiHOME	LC/MS neg	38395	C14829	HMDB04705	10236635	0.90	0.3421
1571		9,10-DiHOME	LC/MS neg	38399	C14828	HMDB04704	9966640	1.02	0.6382
1573		19,20-DiHDPA	LC/MS neg	46286		HMDB10214	16061148	0.85	0.5832
1580	Fatty Acid, Oxidized	4-hydroxy-2-nonenal	LC/MS neg	38309		HMDB04362	1693	0.79	0.5929
1608	Eicosanoid	prostaglandin F2alpha	LC/MS neg	19398	C00639	HMDB01139	5280363	2.46	0.0025
1672		arachidonoyl ethanolamide	LC/MS pos late	32463	C11695	HMDB04080	5281969	0.84	0.3855
1675		N-arachidonoyltaurine	LC/MS neg	54748				0.32	0.0012

1676			N-oleoyltaurine	LC/MS neg	39732			6437033	0.20	0.0000
1677		Endocannabinoid	N-stearoyltaurine	LC/MS neg	39730			168274	0.45	0.0015
1678			N-palmitoyltaurine	LC/MS neg	39835				0.15	0.0000
1680			N-linoleoyltaurine*	LC/MS neg	61826				0.24	0.0002
1682			linoleoyl ethanolamide	LC/MS neg	52608		HMDB12252	5283446	0.74	0.4271
1698			myo-inositol	LC/MS polar	1124	C00137	HMDB00211	892	1.51	0.0003
1699		Inositol Metabolism	chiro-inositol	LC/MS polar	37112	C19891	HMDB34220		4.22	0.0000
1707			inositol 1-phosphate (I1P)	LC/MS polar	43849	C01177	HMDB00213	440194	2.13	0.0004
1727			choline	LC/MS pos early	15506	C00114	HMDB00097	305	1.37	0.0400
1728			choline phosphate	LC/MS pos early	34396	C00588	HMDB01565	1014	0.57	0.0001
1731			glycerophosphorylcholine (GPC)	LC/MS pos early	15990	C00670	HMDB00086	71920	0.68	0.0004
1733		Phospholipid Metabolism	phosphoethanolamine	LC/MS neg	1600	C00346	HMDB00224	1015	1.41	0.0210
1735			glycerophosphoethanolamine	LC/MS polar	37455	C01233	HMDB00114	123874	0.67	0.0041
1736			glycerophosphoserine*	LC/MS pos early	57404			3081457	0.57	0.0009
1737			glycerophosphoinositol*	LC/MS polar	47155			167572	0.50	0.0020
1738			trimethylamine N-oxide	LC/MS pos early	40406	C01104	HMDB00925	1145	1.21	0.3363
1756			1-myristoyl-2-palmitoyl-GPC (14:0/16:0)	LC/MS pos late	19258		HMDB07869	129657	1.06	0.2978
1760			1-myristoyl-2-arachidonoyl-GPC (14:0/20:4)*	LC/MS pos late	53195		HMDB07883		0.62	0.0123
1770			1,2-dipalmitoyl-GPC (16:0/16:0)	LC/MS pos late	19130		HMDB00564	452110	0.96	0.5292
1771			1-palmitoyl-2-palmitoleoyl-GPC (16:0/16:1)*	LC/MS pos late	52470		HMDB07969		0.59	0.0045
1772			1-palmitoyl-2-stearoyl-GPC (16:0/18:0)	LC/MS pos late	52616		HMDB07970		0.95	0.6134
1774			1-palmitoyl-2-oleoyl-GPC (16:0/18:1)	LC/MS pos late	52461		HMDB07972	6436017	1.08	0.1642
1777			1-palmitoyl-2-linoleoyl-GPC (16:0/18:2)	LC/MS pos late	42446		HMDB07973	5287971	1.07	0.2341
1780			1-palmitoyl-2-gamma-linolenoyl-GPC (16:0/18:3n6)*	LC/MS pos late	54812		HMDB07974		1.67	0.0056
1783			1-palmitoyl-2-dihomo-linolenoyl-GPC (16:0/20:3n3 or 6)*	LC/MS pos late	52454				0.45	0.0003
1787			1-palmitoyl-2-arachidonoyl-GPC (16:0/20:4n6)	LC/MS pos late	52462		HMDB07982	10747814	0.93	0.1489
1793		Phosphatidylcholine (PC)	1-palmitoyl-2-docosahexaenoyl-GPC (16:0/22:6)	LC/MS pos late	52610		HMDB07991	6441886	0.95	0.3770
1798			1-palmitoleoyl-2-linoleoyl-GPC (16:1/18:2)*	LC/MS pos late	52683		HMDB08006		0.81	0.0069
1799			1-palmitoleoyl-2-linolenoyl-GPC (16:1/18:3)*	LC/MS pos late	53180		HMDB08008		1.50	0.0164
1809			1-stearoyl-2-oleoyl-GPC (18:0/18:1)	LC/MS pos late	52438		HMDB08038		1.24	0.0038
1812			1-stearoyl-2-linoleoyl-GPC (18:0/18:2)*	LC/MS pos late	52452		HMDB08039		0.92	0.0665
1820			1-stearoyl-2-arachidonoyl-GPC (18:0/20:4)	LC/MS pos late	42450		HMDB08048	16219824	1.13	0.0116
1826			1-stearoyl-2-docosahexaenoyl-GPC (18:0/22:6)	LC/MS pos late	52611		HMDB08057		1.18	0.0022
1833			1-oleoyl-2-linoleoyl-GPC (18:1/18:2)*	LC/MS pos late	52453				0.71	0.0240
1843			1-oleoyl-2-docosahexaenoyl-GPC (18:1/22:6)*	LC/MS pos late	52697		HMDB08123		0.67	0.0051
1844			1,2-dilinoleoyl-GPC (18:2/18:2)	LC/MS pos late	52603		HMDB08138	5288075	0.90	0.3180
1845			1-linoleoyl-2-linolenoyl-GPC (18:2/18:3)*	LC/MS pos late	53176		HMDB08141		2.52	0.0012
1848			1-linoleoyl-2-arachidonoyl-GPC (18:2/20:4n6)*	LC/MS pos late	52710		HMDB08147		0.93	0.0870
1886			1,2-dipalmitoyl-GPE (16:0/16:0)*	LC/MS pos late	57341		HMDB08923	445468	0.66	0.0031
1889			1-palmitoyl-2-stearoyl-GPE (16:0/18:0)*	LC/MS pos late	57388		HMDB08925	5326793	1.17	0.0132
1890			1-palmitoyl-2-oleoyl-GPE (16:0/18:1)	LC/MS pos late	19263		HMDB05320	5283496	0.96	0.7923
1891			1-palmitoyl-2-linoleoyl-GPE (16:0/18:2)	LC/MS pos late	42449		HMDB05322	9546747	0.83	0.1139
1894			1-palmitoyl-2-arachidonoyl-GPE (16:0/20:4)*	LC/MS pos late	52464		HMDB05323	9546800	0.95	0.3341
1896			1-palmitoyl-2-docosahexaenoyl-GPE (16:0/22:6)*	LC/MS polar	52465		HMDB05324	9546799	1.03	0.6678
1903		Phosphatidylethanolamine (PE)	1-stearoyl-2-oleoyl-GPE (18:0/18:1)	LC/MS pos late	42448		HMDB08993		1.57	0.0014
1906			1-stearoyl-2-linoleoyl-GPE (18:0/18:2)*	LC/MS pos late	52446		HMDB08994	9546749	1.19	0.0369
1911			1-stearoyl-2-arachidonoyl-GPE (18:0/20:4)	LC/MS pos late	52447		HMDB09003	5289133	1.17	0.0000
1915			1-stearoyl-2-docosahexaenoyl-GPE (18:0/22:6)*	LC/MS pos late	52466		HMDB05334	9546798	1.13	0.0623
1917			1-oleoyl-2-linoleoyl-GPE (18:1/18:2)*	LC/MS pos late	52687		HMDB05349	9546753	0.85	0.1846
1920			1-oleoyl-2-arachidonoyl-GPE (18:1/20:4)*	LC/MS pos late	55041		HMDB09069		0.99	0.8522
1923			1-oleoyl-2-docosahexaenoyl-GPE (18:1/22:6)*	LC/MS pos late	53209				0.88	0.4011
1924			1,2-dilinoleoyl-GPE (18:2/18:2)*	LC/MS pos late	53174		HMDB09093	9546812	1.58	0.0049
1962			1-stearoyl-2-oleoyl-GPS (18:0/18:1)	LC/MS pos late	19265		HMDB10163	9547087	1.19	0.0167
1963		Phosphatidylserine (PS)	1-stearoyl-2-linoleoyl-GPS (18:0/18:2)	LC/MS pos late	53009			9547091	1.15	0.0939
1965			1-stearoyl-2-arachidonoyl-GPS (18:0/20:4)	LC/MS pos late	52235		HMDB12383		1.06	0.1777
1975	Lipid	Phosphatidylglycerol (PG)	1-palmitoyl-2-oleoyl-GPG (16:0/18:1)	LC/MS pos late	52448			5283509	1.14	0.1331
1976			1-palmitoyl-2-linoleoyl-GPG (16:0/18:2)	LC/MS polar	52628		HMDB10575		1.14	0.1951
1989			1-palmitoyl-2-oleoyl-GPI (16:0/18:1)*	LC/MS pos late	52669		HMDB09783		1.17	0.3780
1991			1-palmitoyl-2-linoleoyl-GPI (16:0/18:2)	LC/MS pos late	52450		HMDB09784		1.18	0.1072

1993	Phosphatidylinositol (PI)	1-palmitoyl-2-arachidonoyl-GPI (16:0/20:4)*	LC/MS pos late	52467		HMDB09789		0.61	0.0000
1997		1-stearoyl-2-linoleoyl-GPI (18:0/18:2)	LC/MS pos late	52468		HMDB09809		1.61	0.0149
2008		1-stearoyl-2-arachidonoyl-GPI (18:0/20:4)	LC/MS pos late	52449		HMDB09815		1.10	0.0166
2013		1-oleoyl-2-arachidonoyl-GPI (18:1/20:4) *	LC/MS pos late	54994		HMDB09844		0.74	0.0398
2045	Lysophospholipid	1-palmitoyl-GPC (16:0)	LC/MS pos late	33955		HMDB10382	86554	1.05	0.5358
2046		2-palmitoyl-GPC (16:0)*	LC/MS neg	35253		HMDB61702	15061532	1.03	0.8654
2047		1-palmitoleoyl-GPC (16:1)*	LC/MS pos late	33230		HMDB10383	24779461	0.78	0.0173
2048		2-palmitoleoyl-GPC (16:1)*	LC/MS pos late	35819		HMDB10383		0.40	0.0062
2053		1-stearoyl-GPC (18:0)	LC/MS pos late	33961		HMDB10384	497299	1.35	0.0093
2055		1-oleoyl-GPC (18:1)	LC/MS pos late	48258		HMDB02815	16081932	0.96	0.6188
2058		1-linoleoyl-GPC (18:2)	LC/MS pos late	34419	C04100	HMDB10386	11988421	1.12	0.4864
2075		1-arachidonoyl-GPC (20:4n6)*	LC/MS neg	34061	C05208	HMDB10395		1.10	0.4525
2086		1-lignoceroyl-GPC (24:0)	LC/MS pos late	49617		HMDB10405		1.58	0.0087
2093		1-palmitoyl-GPE (16:0)	LC/MS pos late	35631		HMDB11503	9547069	1.07	0.5124
2100		1-stearoyl-GPE (18:0)	LC/MS pos late	42398		HMDB11130	9547068	1.46	0.0116
2101		2-stearoyl-GPE (18:0)*	LC/MS neg	41220		HMDB11129		1.32	0.0993
2102		1-oleoyl-GPE (18:1)	LC/MS pos late	35628		HMDB11506	9547071	1.03	0.9954
2104		1-linoleoyl-GPE (18:2)*	LC/MS pos late	36600		HMDB11507	52925130	1.05	0.8340
2115		1-arachidonoyl-GPE (20:4n6)*	LC/MS neg	35186		HMDB11517	42607465	1.01	0.8832
2124		1-palmitoyl-GPS (16:0)*	LC/MS neg	46130			9547100	0.54	0.0041
2125		1-stearoyl-GPS (18:0)*	LC/MS polar	45966			9547101	1.64	0.1149
2126		1-oleoyl-GPS (18:1)	LC/MS neg	19260		HMDB61694	9547099	0.97	0.6289
2128		1-linoleoyl-GPS (18:2)*	LC/MS neg	43676				0.97	0.6433
2131		1-palmitoyl-GPG (16:0)*	LC/MS neg	45970			3300276	1.32	0.0658
2133		1-stearoyl-GPG (18:0)	LC/MS neg	34437				1.67	0.0030
2135		1-oleoyl-GPG (18:1)*	LC/MS neg	45968				1.36	0.0642
2137		1-linoleoyl-GPG (18:2)*	LC/MS neg	54885				1.39	0.0136
2138		1-palmitoyl-GPI (16:0)	LC/MS neg	35305		HMDB61695		0.61	0.0005
2141		1-stearoyl-GPI (18:0)	LC/MS neg	19324		HMDB61696		0.99	0.8104
2143		1-oleoyl-GPI (18:1)*	LC/MS neg	36602				0.85	0.3782
2145		1-linoleoyl-GPI (18:2)*	LC/MS neg	36594				1.46	0.0712
2149		1-arachidonoyl-GPI (20:4)*	LC/MS neg	34214		HMDB61690		1.17	0.2257
2238	Plasmalogen	1-(1-enyl-palmitoyl)-2-oleoyl-GPE (P-16:0/18:1)*	LC/MS pos late	52477		HMDB11342		1.08	0.4005
2239		1-(1-enyl-palmitoyl)-2-linoleoyl-GPE (P-16:0/18:2)*	LC/MS pos late	52677		HMDB11343		1.20	0.3915
2240		1-(1-enyl-palmitoyl)-2-palmitoyl-GPC (P-16:0/16:0)*	LC/MS pos late	52716		HMDB11206	11146967	1.10	0.2465
2242		1-(1-enyl-palmitoyl)-2-arachidonoyl-GPE (P-16:0/20:4)*	LC/MS pos late	52673		HMDB11352		1.20	0.0220
2246		1-(1-enyl-stearoyl)-2-oleoyl-GPE (P-18:0/18:1)	LC/MS pos late	52614		HMDB11375		1.37	0.0848
2254		1-(1-enyl-stearoyl)-2-arachidonoyl-GPE (P-18:0/20:4)*	LC/MS pos late	52475		HMDB05779	9547058	1.07	0.3015
2270	Lysoplasmalogen	1-(1-enyl-palmitoyl)-GPE (P-16:0)*	LC/MS neg	39270				1.47	0.0751
2274		1-(1-enyl-stearoyl)-GPE (P-18:0)*	LC/MS pos late	39271				1.23	0.2235
2277	Glycerolipid Metabolism	glycerol	LC/MS neg	15122	C00116	HMDB00131	753	0.94	0.4975
2278		glycerol 3-phosphate	LC/MS polar	43847	C00093	HMDB00126	754	0.78	0.0185
2283		glycerophosphoglycerol	LC/MS polar	48857	C03274		439964	0.72	0.0045
2284	Monoacylglycerol	1-myristoylglycerol (14:0)	LC/MS neg	35625	C01885	HMDB11561	79050	0.77	0.2993
2287		1-palmitoylglycerol (16:0)	LC/MS neg	21127		HMDB31074	14900	0.66	0.3037
2288		1-palmitoleoylglycerol (16:1)*	LC/MS neg	52431		HMDB11565		0.34	0.0077
2291		1-oleoylglycerol (18:1)	LC/MS neg	21184		HMDB11567	5283468	0.73	0.0460
2292		1-linoleoylglycerol (18:2)	LC/MS neg	27447			5283469	0.78	0.4707
2293		1-linolenoylglycerol (18:3)	LC/MS neg	34393		HMDB11569	53480978	0.76	0.3046
2297		1-dihomo-linolenylglycerol (20:3)	LC/MS neg	48341				0.68	0.2786
2298		1-arachidonylglycerol (20:4)	LC/MS neg	34397	C13857	HMDB11549	5282281	1.36	0.1262
2301		1-docosahexaenoylglycerol (22:6)	LC/MS neg	35153		HMDB11587		1.15	0.4297
2304		2-palmitoleoylglycerol (16:1)*	LC/MS neg	52432		HMDB11565		0.33	0.0081
2306		2-oleoylglycerol (18:1)	LC/MS neg	21232		HMDB11537	5319879	0.56	0.0049
2307		2-linoleoylglycerol (18:2)	LC/MS neg	32506		HMDB11538	5365676	0.79	0.1668
2308		2-arachidonoylglycerol (20:4)	LC/MS neg	19266	C13856	HMDB04666	5282280	1.35	0.1409
2310		2-docosahexaenoylglycerol (22:6)*	LC/MS neg	48675		HMDB11557		1.15	0.5040
2314		1-meadoylglycerol (20:3n9)*	LC/MS neg	62431				1.07	0.7271
2316		1-dihomo-linoleoylglycerol (20:2)	LC/MS neg	35103				0.70	0.3727

2320		diacylglycerol (14:0/18:1, 16:0/16:1) [2]*	LC/MS pos late	54954				0.35	0.0023
2321		diacylglycerol (16:1/18:2 [2], 16:0/18:3 [1])*	LC/MS pos late	54966				0.45	0.0001
2337		palmitoyl-oleoyl-glycerol (16:0/18:1) [2]*	LC/MS pos late	54942	C13861	HMDB07102		0.70	0.0254
2338		palmitoyl-linoleoyl-glycerol (16:0/18:2) [1]*	LC/MS pos late	52633		HMDB07103	9543695	0.73	0.0125
2339		palmitoyl-linoleoyl-glycerol (16:0/18:2) [2]*	LC/MS pos late	52634		HMDB07103		0.83	0.0469
2343		palmitoleoyl-linoleoyl-glycerol (16:1/18:2) [1]*	LC/MS pos late	54967		HMDB07132		0.41	0.0014
2345		palmitoyl-arachidonoyl-glycerol (16:0/20:4) [1]*	LC/MS pos late	54957		HMDB07112		1.48	0.0074
2346		palmitoyl-arachidonoyl-glycerol (16:0/20:4) [2]*	LC/MS pos late	54958		HMDB07112		1.07	0.6929
2347		palmitoleoyl-arachidonoyl-glycerol (16:1/20:4) [2]*	LC/MS pos late	57406				1.27	0.2846
2348		palmitoyl-docosahexaenoyl-glycerol (16:0/22:6) [1]*	LC/MS pos late	57373		HMDB07121		1.07	0.6383
2349		palmitoyl-docosahexaenoyl-glycerol (16:0/22:6) [2]*	LC/MS pos late	57374		HMDB07121		1.31	0.1029
2352		stearoyl-linoleoyl-glycerol (18:0/18:2) [2]*	LC/MS pos late	54947				1.32	0.0747
2355		oleoyl-oleoyl-glycerol (18:1/18:1) [2]*	LC/MS pos late	54946		HMDB07218		0.53	0.0020
2357	Diacylglycerol	oleoyl-linoleoyl-glycerol (18:1/18:2) [1]	LC/MS pos late	46798		HMDB07219		0.71	0.0631
2358		oleoyl-linoleoyl-glycerol (18:1/18:2) [2]	LC/MS pos late	46799		HMDB07219		0.75	0.0456
2360		oleoyl-linolenoyl-glycerol (18:1/18:3) [2]*	LC/MS pos late	54970		HMDB07220		0.32	0.0126
2362		linoleoyl-linoleoyl-glycerol (18:2/18:2) [1]*	LC/MS pos late	54968		HMDB07248		0.93	0.9661
2363		linoleoyl-linoleoyl-glycerol (18:2/18:2) [2]*	LC/MS pos late	54969		HMDB07248		1.07	0.6238
2364		linoleoyl-linolenoyl-glycerol (18:2/18:3) [1]*	LC/MS pos late	54963		HMDB07249		0.70	0.1506
2365		linoleoyl-linolenoyl-glycerol (18:2/18:3) [2]*	LC/MS pos late	54964		HMDB07250		0.58	0.1316
2368		stearoyl-arachidonoyl-glycerol (18:0/20:4) [1]*	LC/MS pos late	57450				1.55	0.0060
2369		stearoyl-arachidonoyl-glycerol (18:0/20:4) [2]*	LC/MS pos late	57449				1.41	0.0016
2370		oleoyl-arachidonoyl-glycerol (18:1/20:4) [1]*	LC/MS pos late	54960		HMDB07228		0.76	0.9410
2371		oleoyl-arachidonoyl-glycerol (18:1/20:4) [2]*	LC/MS pos late	54961		HMDB07228		1.01	0.8104
2372		linoleoyl-arachidonoyl-glycerol (18:2/20:4) [1]*	LC/MS pos late	54955		HMDB07257		1.01	0.9160
2373		linoleoyl-arachidonoyl-glycerol (18:2/20:4) [2]*	LC/MS pos late	54956		HMDB07257		1.86	0.0239
2375		stearoyl-docosahexaenoyl-glycerol (18:0/22:6) [2]*	LC/MS pos late	57368				1.45	0.0178
2400		sphinganine	LC/MS pos late	17769	C00836	HMDB00269	3126	1.06	0.5295
2402	Sphingolipid Synthesis	sphingadienine	LC/MS pos late	57426			6449795	0.82	0.2236
2403		phytosphingosine	LC/MS pos late	1510	C12144	HMDB04610	122121	1.58	0.0010
2406	Dihydroceramides	N-palmitoyl-sphinganine (d18:0/16:0)	LC/MS pos late	52604		HMDB11760	5283572	0.60	0.0000
2414		N-palmitoyl-sphingosine (d18:1/16:0)	LC/MS pos late	44877		HMDB04949	5283564	0.89	0.0757
2417		N-stearoyl-sphingosine (d18:1/18:0)*	LC/MS pos late	54979		HMDB04950	5283565	1.14	0.2129
2419		N-arachidoyl-sphingosine (d18:1/20:0)*	LC/MS pos late	57424		HMDB04951	5283566	1.11	0.1444
2425	Ceramides	N-behenoyl-sphingadienine (d18:2/22:0)*	LC/MS pos late	57372				1.34	0.0068
2439		ceramide (d18:1/14:0, d16:1/16:0)*	LC/MS pos late	57432				0.51	0.0001
2440		ceramide (d18:1/17:0, d17:1/18:0)*	LC/MS pos late	57434				1.24	0.0415
2443		ceramide (d16:1/24:1, d18:1/22:1)*	LC/MS pos late	57437				0.82	0.0726
2444		ceramide (d18:2/24:1, d18:1/24:2)*	LC/MS pos late	57443				1.05	0.4746
2448		glycosyl-N-palmitoyl-sphingosine (d18:1/16:0)	LC/MS pos late	53013				1.47	0.0004
2449		glycosyl-N-stearoyl-sphingosine (d18:1/18:0)	LC/MS pos late	52234				1.56	0.0002
2454	Hexosylceramides (HCER)	glycosyl-N-nervonoyl-sphingosine (d18:1/24:1)*	LC/MS pos late	57369				1.44	0.0076
2469		glycosyl ceramide (d18:1/20:0, d16:1/22:0)*	LC/MS pos late	57595				1.58	0.0001
2473		glycosyl ceramide (d18:2/24:1, d18:1/24:2)*	LC/MS pos late	57453				1.65	0.0028
2475	Lactosylceramides (LCER)	lactosyl-N-palmitoyl-sphingosine (d18:1/16:0)	LC/MS pos late	53010				0.86	0.2703
2479		lactosyl-N-nervonoyl-sphingosine (d18:1/24:1)*	LC/MS pos late	57370				1.00	0.8753
2487		palmitoyl dihydrosphingomyelin (d18:0/16:0)*	LC/MS pos late	52434			9939965	0.76	0.0003
2488	Dihydrosphingomyelins	behenoyl dihydrosphingomyelin (d18:0/22:0)*	LC/MS pos late	57331		HMDB12091		0.72	0.0230
2489		sphingomyelin (d18:0/18:0, d19:0/17:0)*	LC/MS pos late	57473		HMDB12087		1.01	0.9985
2491		palmitoyl sphingomyelin (d18:1/16:0)	LC/MS pos late	37506			9939941	1.02	0.7653
2492		stearoyl sphingomyelin (d18:1/18:0)	LC/MS pos late	19503	C00550	HMDB01348	6453725	0.99	0.7189
2493		behenoyl sphingomyelin (d18:1/22:0)*	LC/MS pos late	48492		HMDB12103		1.16	0.0977
2494		tricosanoyl sphingomyelin (d18:1/23:0)*	LC/MS pos late	52436		HMDB12105		1.26	0.0009
2495		lignoceroyl sphingomyelin (d18:1/24:0)	LC/MS pos late	57330				1.18	0.0590
2497		sphingomyelin (d18:2/23:1)*	LC/MS pos late	57482				1.23	0.0550
2498		sphingomyelin (d18:2/24:2)*	LC/MS pos late	57479				1.21	0.3601
2500		sphingomyelin (d18:1/14:0, d16:1/16:0)*	LC/MS pos late	42463		HMDB12097	11433862	0.61	0.0002
2502		sphingomyelin (d17:1/16:0, d18:1/15:0, d16:1/17:0)*	LC/MS pos late	52433				0.75	0.0118
2504		sphingomyelin (d18:2/16:0, d18:1/16:1)*	LC/MS pos late	42459				1.01	0.9842

2505		Sphingomyelins	sphingomyelin (d18:1/17:0, d17:1/18:0, d19:1/16:0)	LC/MS pos late	52615				1.36	0.0000
2506			sphingomyelin (d18:1/18:1, d18:2/18:0)	LC/MS pos late	37529		HMDB12101	6443882	1.46	0.0005
2507			sphingomyelin (d18:1/19:0, d19:1/18:0)*	LC/MS pos late	57475				1.19	0.0046
2508			sphingomyelin (d18:1/20:0, d16:1/22:0)*	LC/MS pos late	48490		HMDB12102		1.03	0.2879
2509			sphingomyelin (d18:1/20:1, d18:2/20:0)*	LC/MS pos late	48491				1.06	0.4557
2511			sphingomyelin (d18:1/21:0, d17:1/22:0, d16:1/23:0)*	LC/MS pos late	52495				1.25	0.0039
2513			sphingomyelin (d18:1/22:1, d18:2/22:0, d16:1/24:1)*	LC/MS pos late	48493		HMDB12104		0.94	0.0962
2514			sphingomyelin (d18:1/22:2, d18:2/22:1, d16:1/24:2)*	LC/MS pos late	57477				0.72	0.0042
2515			sphingomyelin (d18:2/23:0, d18:1/23:1, d17:1/24:1)*	LC/MS pos late	52435				1.27	0.0102
2516			sphingomyelin (d18:1/24:1, d18:2/24:0)*	LC/MS pos late	47153		HMDB12107		1.07	0.3841
2517			sphingomyelin (d18:2/24:1, d18:1/24:2)*	LC/MS pos late	52437				0.98	0.6365
2518			sphingomyelin (d18:1/25:0, d19:0/24:1, d20:1/23:0, d19:	LC/MS pos late	57478				0.96	0.8549
2519		Sphingosines	sphingosine	LC/MS pos late	17747	C00319	HMDB00252	5353955	1.32	0.0236
2527			hexadecasphingosine (d16:1)*	LC/MS pos late	57428				1.16	0.0933
2535		Mevalonate Metabolism	3-hydroxy-3-methylglutarate	LC/MS polar	531	C03761	HMDB00355	1662	1.83	0.0103
2550		Sterol	cholesterol	LC/MS pos late	63	C00187	HMDB00067	11025495	1.24	0.0003
2561			7-alpha-hydroxy-3-oxo-4-cholestenoate (7-Hoca)	LC/MS neg	36776	C17337	HMDB12458	3081085	2.93	0.0533
2574			4-cholesten-3-one	LC/MS pos late	38125	C00599	HMDB00921	91477	0.86	0.0279
2575			beta-sitosterol	LC/MS pos late	27414	C01753	HMDB00852	222284	0.67	0.0175
2577			campesterol	LC/MS pos late	33997	C01789	HMDB02869	173183	0.78	0.2692
2594			7-hydroxycholesterol (alpha or beta)	LC/MS pos late	47890		HMDB06119	107722	1.49	0.1708
2648		Corticosteroids	corticosterone	LC/MS neg	5983	C02140	HMDB01547	5753	0.32	0.0187
2674		Androgenic Steroids	epiandrosterone sulfate	LC/MS neg	33973			9929317	1.94	0.0108
2743		Primary Bile Acid Metabolism	cholate	LC/MS neg	22842	C00695	HMDB00619	221493	2.03	0.3269
2744			glycocholate	LC/MS neg	18476	C01921	HMDB00138	10140	8.15	0.0001
2745			taurocholate	LC/MS neg	18497	C05122	HMDB00036	6675	1.45	0.1798
2746			chenodeoxycholate	LC/MS neg	1563	C02528	HMDB00518	10133	4.55	0.0428
2747			glycochenodeoxycholate	LC/MS neg	32346	C05466	HMDB00637	12544	16.40	0.0000
2748			taurochenodeoxycholate	LC/MS neg	18494	C05465	HMDB00951	387316	2.38	0.0001
2749			beta-muricholate	LC/MS neg	31885	C17726	HMDB00415	5283853	1.09	0.7324
2750			alpha-muricholate	LC/MS neg	34092	C17647	HMDB00506	3083761	2.41	0.2965
2751			tauro-alpha-muricholate	LC/MS neg	42605				1.84	0.0206
2752			tauro-beta-muricholate	LC/MS neg	33983		HMDB00932	168408	0.87	0.3750
2754			glyco-alpha-muricholate**	LC/MS neg	62065				12.96	0.0002
2755			glyco-beta-muricholate**	LC/MS neg	62066				6.02	0.0005
2763		Secondary Bile Acid Metabolism	deoxycholate	LC/MS neg	1114	C04483	HMDB00626	222528	2.08	0.4966
2765			glycodeoxycholate	LC/MS neg	18477	C05464	HMDB00631	3035026	8.81	0.0015
2766			taurodeoxycholate	LC/MS neg	12261	C05463	HMDB00896	2733768	1.10	0.7355
2774			taurolithocholate	LC/MS neg	31889	C02592	HMDB00722	10595	7.41	0.0000
2775			taurolithocholate 3-sulfate	LC/MS neg	36850	C03642	HMDB02580	440071	1.59	0.0199
2780			glycoursodeoxycholate	LC/MS neg	39379		HMDB00708	12310288	8.85	0.0000
2782			tauroursodeoxycholate	LC/MS neg	39378		HMDB00874	9848818	1.35	0.1565
2786			6-oxolithocholate	LC/MS neg	46336			164960	3.96	0.0198
2790			taurohyocholate*	LC/MS neg	42603				0.66	0.1732
2792			taurohyodeoxycholic acid	LC/MS neg	43588			119046	2.56	0.0183
2793			glycohyodeoxycholate	LC/MS neg	43501			114611	20.97	0.0000
2799			taurocholenate sulfate*	LC/MS neg	32807				2.33	0.0373
2800			7-ketodeoxycholate	LC/MS neg	31904		HMDB00391	188292	2.09	0.6073
3374		Purine Metabolism, (Hypo)Xanthine/Inosine containing	inosine	LC/MS pos early	1123	C00294	HMDB00195	6021	0.81	0.0089
3375			hypoxanthine	LC/MS polar	3127	C00262	HMDB00157	790	0.99	0.8040
3376			xanthine	LC/MS polar	3147	C00385	HMDB00292	1188	0.95	0.0299
3378			xanthosine	LC/MS neg	15136	C01762	HMDB00299	64959	0.86	0.1602
3382			2'-deoxyinosine	LC/MS neg	15076	C05512	HMDB00071	65058	0.91	0.9836
3384			urate	LC/MS neg	1604	C00366	HMDB00289	1175	0.73	0.0090
3386			allantoin	LC/MS polar	1107	C02350	HMDB00462	204	1.12	0.1270
3387			allantoic acid	LC/MS polar	33434	C00499	HMDB01209	203	1.93	0.0000
3392			adenosine 5'-monophosphate (AMP)	LC/MS neg	32342	C00020	HMDB00045	6083	1.68	0.0015
3393			adenosine 3'-monophosphate (3'-AMP)	LC/MS neg	35142	C01367	HMDB03540	41211	1.06	0.6802
3394			adenosine 2'-monophosphate (2'-AMP)	LC/MS neg	36815	C00946	HMDB11617	94136	1.11	0.0660

3397			adenosine 3',5'-diphosphate	LC/MS neg	15691	C00054	HMDB00061	159296	1.25	0.1121
3399		Purine Metabolism, Adenine containing	adenosine	LC/MS pos early	555	C00212	HMDB00050	60961	0.98	0.9167
3400			adenine	LC/MS pos early	554	C00147	HMDB00034	190	0.77	0.0097
3405			N1-methyladenosine	LC/MS pos early	15650	C02494	HMDB03331	27476	1.50	0.2593
3418			2'-deoxyadenosine 5'-monophosphate	LC/MS neg	46333	C00360	HMDB00905	12599	1.21	0.3298
3424			N6-succinyladenosine	LC/MS neg	48130		HMDB00912	165243	3.87	0.0001
3427			guanosine 5'- monophosphate (5'-GMP)	LC/MS pos early	2849	C00144	HMDB01397	6804	2.31	0.0060
3432			guanosine	LC/MS neg	1573	C00387	HMDB00133	6802	1.47	0.0520
3433		Purine Metabolism, Guanine containing	guanine	LC/MS pos early	32352	C00242	HMDB00132	764	1.16	0.2506
3435			7-methylguanine	LC/MS neg	35114	C02242	HMDB00897	11361	2.97	0.0000
3449			2'-deoxyguanosine	LC/MS neg	1411	C00330	HMDB00085	187790	1.38	0.2032
3453			dihydroorotate	LC/MS polar	601	C00337	HMDB03349	648	1.50	0.0207
3454		Pyrimidine Metabolism, Orotate containing	orotate	LC/MS polar	1505	C00295	HMDB00226	967	1.95	0.1054
3456	Nucleotide		orotidine	LC/MS polar	35172		HMDB00788	92751	1.92	0.0001
3463			uridine 3'-monophosphate (3'-UMP)	LC/MS polar	39764	C01368		101543	2.83	0.0052
3465			uridine-2',3'-cyclic monophosphate	LC/MS neg	37137	C02355	HMDB11640	439715	3.43	0.0020
3466			uridine	LC/MS neg	606	C00299	HMDB00296	6029	0.94	0.4290
3467			uracil	LC/MS pos early	605	C00106	HMDB00300	1174	1.59	0.0141
3468			pseudouridine	LC/MS pos early	33442	C02067	HMDB00767	15047	3.16	0.0000
3469		Pyrimidine Metabolism, Uracil containing	5,6-dihydrouridine	LC/MS neg	61833			94312	4.62	0.0001
3470			2'-O-methyluridine	LC/MS neg	57655			102212	1.45	0.0340
3471			5-methyluridine (ribothymidine)	LC/MS neg	35136		HMDB00884	445408	0.90	0.9205
3482			2'-deoxyuridine	LC/MS neg	52602	C00526	HMDB00012	13712	0.89	0.7600
3486			beta-alanine	LC/MS pos early	55	C00099	HMDB00056	239	1.61	0.0002
3489			uridine 2'-monophosphate (2'-UMP)*	LC/MS neg	52444				4.28	0.0016
3492			cytidine 5'-monophosphate (5'-CMP)	LC/MS pos early	2372	C00055	HMDB00095	6131	1.08	0.2568
3496			cytidine	LC/MS pos early	514	C00475	HMDB00089	6175	1.05	0.6105
3497			cytosine	LC/MS pos early	573	C00380	HMDB00630	597	1.61	0.0614
3498			3-methylcytidine	LC/MS pos early	35132			159649	2.28	0.0000
3499			5-methylcytidine	LC/MS pos early	22119		HMDB00982	92918	0.91	0.0548
3500		Pyrimidine Metabolism, Cytidine containing	N4-acetylcytidine	LC/MS neg	35130		HMDB05923	107461	3.27	0.0001
3504			2'-deoxycytidine 5'-monophosphate	LC/MS pos early	533	C00239	HMDB01202	13945	0.97	0.8368
3506			2'-deoxycytidine	LC/MS pos early	15949	C00881	HMDB00014	13711	0.89	0.1143
3507			2'-O-methylcytidine	LC/MS pos early	57554			150971	1.70	0.0084
3508			5-methyl-2'-deoxycytidine	LC/MS pos early	38159	C03592	HMDB02224	440055	1.41	0.0049
3517			thymidine	LC/MS neg	2183	C00214	HMDB00273	5789	1.47	0.0969
3518		Pyrimidine Metabolism, Thymine containing	thymine	LC/MS neg	604	C00178	HMDB00262	1135	0.64	0.1391
3521			3-aminoisobutyrate	LC/MS pos early	1566	C05145	HMDB03911	64956	1.43	0.1046
3522		Purine and Pyrimidine Metabolism	methylphosphate	LC/MS pos early	37070		HMDB61711	13130	0.76	0.3385
3539			nicotinate ribonucleoside	LC/MS pos early	33471	C05841	HMDB06809	161234	2.30	0.0058
3541			nicotinamide	LC/MS pos early	594	C00153	HMDB01406	936	1.01	0.7906
3543			nicotinamide ribonucleotide (NMN)	LC/MS pos early	22152	C00455	HMDB00229	14180	0.94	0.9995
3544			nicotinamide riboside	LC/MS pos early	33013	C03150	HMDB00855	439924	1.03	0.7467
3545		Nicotinate and Nicotinamide Metabolism	nicotinamide adenine dinucleotide (NAD+)	LC/MS neg	5278	C00003	HMDB00902	5893	0.92	0.4405
3553			1-methylnicotinamide	LC/MS pos early	27665	C02918	HMDB00699	10129985	0.94	0.5928
3558			trigonelline (N'-methylnicotinate)	LC/MS pos early	32401	C01004	HMDB00875	5570	2.09	0.0000
3560			N1-Methyl-2-pyridone-5-carboxamide	LC/MS neg	40469	C05842	HMDB04193	69698	3.11	0.0061
3561			N1-Methyl-4-pyridone-3-carboxamide	LC/MS pos early	57584	C05843	HMDB04194	440810	1.89	0.0002
3562			adenosine 5'-diphosphoribose (ADP-ribose)	LC/MS pos early	558	C00301	HMDB01178	192	0.95	0.7684
3565			riboflavin (Vitamin B2)	LC/MS pos early	1827	C00255	HMDB00244	493570	0.91	0.2628
3566		Riboflavin Metabolism	flavin adenine dinucleotide (FAD)	LC/MS neg	2134	C00016	HMDB01248	643975	1.09	0.1556
3567			flavin mononucleotide (FMN)	LC/MS neg	15797	C00061	HMDB01520	710	0.74	0.0343
3568			pantothenate	LC/MS neg	1508	C00864	HMDB00210	6613	1.17	0.0090
3570			phosphopantetheine	LC/MS neg	15504	C01134	HMDB01416	987	0.72	0.1875
3571		Pantothenate and CoA Metabolism	3'-dephosphocoenzyme A	LC/MS neg	18289	C00882	HMDB01373	444485	1.01	0.9177
3573			coenzyme A	LC/MS neg	46322	C00010	HMDB01423	317	1.03	0.6622
3575			pantetheine	LC/MS neg	57555	C00831		439322	0.51	0.1709
3579			ascorbate (Vitamin C)	LC/MS pos early	32354	C00072	HMDB00044		0.70	0.0170
3580			dehydroascorbate	LC/MS neg	34302	C05422	HMDB01264	835	0.52	0.0035

3581		Ascorbate and Aldarate Metabolism	threonate	LC/MS polar	27738	C01620	HMDB00943	151152	0.80	0.0639
3584			oxalate (ethanedioate)	LC/MS neg	20694	C00209	HMDB02329	971	0.70	0.0397
3585	Cofactors and Vitamins		gulonate*	LC/MS polar	46957	C00257	HMDB03290	9794176	1.24	0.3505
3587			alpha-tocopherol	LC/MS pos late	1561	C02477	HMDB01893	14985	1.09	0.2768
3598			alpha-CEHC sulfate	LC/MS neg	47666				1.46	0.3490
3601		Tocopherol Metabolism	alpha-CEHC	LC/MS neg	48188		HMDB01518	9943542	1.75	0.0159
3606			gamma-CEHC sulfate*	LC/MS neg	62280				3.37	0.0000
3609			gamma-tocopherol/beta-tocopherol	LC/MS pos late	52473				0.95	0.4756
3615		Folate Metabolism	5-methyltetrahydrofolate (5MeTHF)	LC/MS neg	18330	C00440	HMDB01396	146	0.95	0.6031
3616		Tetrahydrobiopterin Metabolism	biopterin	LC/MS neg	12358	C06313	HMDB00468	445040	1.16	0.1387
3617			dihydrobiopterin	LC/MS pos early	35129	C00268	HMDB00038	1879	0.99	0.9307
3620		Pterin Metabolism	pterin	LC/MS neg	43023	C00715	HMDB00802	73000	0.97	0.6422
3631			heme	LC/MS pos late	41754	C00032	HMDB03178	26945	2.08	0.0234
3632		Hemoglobin and Porphyrin Metabolism	bilirubin (Z,Z)	LC/MS pos late	43807	C00486	HMDB00054	5280352	1.21	0.2938
3635			biliverdin	LC/MS pos late	2137	C00500	HMDB01008	5353439	1.02	0.8018
3644			thiamin (Vitamin B1)	LC/MS pos early	5341	C00378	HMDB00235	1130	1.40	0.0001
3645		Thiamine Metabolism	thiamin monophosphate	LC/MS pos early	15798	C01081	HMDB02666	3382778	1.29	0.0008
3646			thiamin diphosphate	LC/MS neg	35670	C00068	HMDB01372	1132	1.28	0.5962
3652			retinol (Vitamin A)	LC/MS pos late	1806	C00473	HMDB00305	445354	1.06	0.5468
3654		Vitamin A Metabolism	carotene diol (1)	LC/MS pos late	57635				1.35	0.2272
3659			retinal	LC/MS pos late	40807	C00376	HMDB01358	638015	0.77	0.2078
3668			pyridoxamine	LC/MS pos early	2150	C00534	HMDB01431	1052	1.38	0.0170
3669			pyridoxamine phosphate	LC/MS pos early	3138	C00647	HMDB01555	1053	1.05	0.5175
3670		Vitamin B6 Metabolism	pyridoxal phosphate	LC/MS neg	5331	C00018	HMDB01491	1051	1.14	0.2792
3671			pyridoxal	LC/MS pos early	1651	C00250	HMDB01545	1050	1.12	0.2645
3672			pyridoxate	LC/MS neg	31555	C00847	HMDB00017	6723	1.47	0.2470
3674			hippurate	LC/MS neg	15753	C01586	HMDB00714	464	2.03	0.0008
3678			2-hydroxyhippurate (salicylurate)	LC/MS polar	18281	C07588	HMDB00840	10253	4.72	0.0000
3682			4-hydroxyhippurate	LC/MS neg	35527		HMDB13678	151012	0.80	0.5010
3688			benzoate	LC/MS neg	15778	C00180	HMDB01870	243	0.70	0.3374
3706		Benzoate Metabolism	catechol sulfate	LC/MS neg	35320		HMDB59724	3083879	4.31	0.0000
3709			guaiacol sulfate	LC/MS neg	46111		HMDB60013	22473	2.83	0.0007
3724			4-ethylphenylsulfate	LC/MS neg	36099	C13637			3.00	0.0000
3746			p-cresol sulfate	LC/MS neg	36103		HMDB11635	4615423	9.04	0.0073
3757			3-(3-hydroxyphenyl)propionate sulfate	LC/MS neg	45415			187488	1.27	0.6891
3762			3-phenylpropionate (hydrocinnamate)	LC/MS neg	15749	C05629	HMDB00764	107	2.19	0.0590
3845			genistein	LC/MS neg	32448	C06563	HMDB03217	5280961	5.00	0.0004
3846			genistein glucuronide*	LC/MS neg	62063				4.91	0.0001
3910			betonicine	LC/MS pos early	38100	C08269	HMDB29412	164642	3.26	0.0000
3914			gluconate	LC/MS polar	587	C00257	HMDB00625	10690	0.32	0.1919
3934			beta-guanidinopropanoate	LC/MS pos early	35101	C03065	HMDB13222	67701	0.70	0.0062
3956			cinnamoylglycine	LC/MS neg	38637		HMDB11621	709625	1.74	0.1231
3973			daidzein	LC/MS neg	32453	C10208	HMDB03312	5281708	3.04	0.0035
3976			genistein sulfate*	LC/MS neg	62061				4.65	0.0006
3984			enterolactone	LC/MS neg	39626			10685477	3.16	0.0000
3986			equol	LC/MS neg	36774	C14131	HMDB02209	91469	2.93	0.0001
3987			equol glucuronide	LC/MS neg	41948				3.69	0.0004
3988	Xenobiotics		equol sulfate	LC/MS neg	40478				2.35	0.0005
3989		Food Component/Plant	ergothioneine	LC/MS pos early	37459	C05570	HMDB03045	3032311	0.86	0.3477
3991			erythritol	LC/MS polar	20699	C00503	HMDB02994	222285	3.90	0.0000
3994			ferulic acid 4-sulfate	LC/MS neg	47114		HMDB29200	6305574	3.13	0.0017
3997			formononetin	LC/MS neg	35657	C00858	HMDB05808	5280378	5.24	0.0003
4013			histidine betaine (hercynine)*	LC/MS pos early	62278			3083620	0.76	0.1815
4016			homostachydrine*	LC/MS pos early	33009	C08283	HMDB33433	441447	1.98	0.0000
4019			indoleacrylate	LC/MS neg	22114		HMDB00734	15030923	0.92	0.5943
4051			N-glycolylneuraminate	LC/MS pos early	37123	C03410	HMDB00833	123802	1.80	0.0013
4128			stachydrine	LC/MS pos early	34384	C10172	HMDB04827	115244	3.16	0.0000
4152			methyl glucopyranoside (alpha + beta)	LC/MS neg	46144				1.06	0.7181
4183			daidzein sulfate (2)	LC/MS neg	48728				8.86	0.0006

4188			daidzein sulfate (1)	LC/MS neg	48727				2.07	0.0160
4211			4-ethylphenol glucuronide	LC/MS neg	52937				0.85	0.2140
4268		Bacterial/Fungal	tartronate (hydroxymalonate)	LC/MS neg	20693	C02287	HMDB35227	45	0.61	0.0026
4655		Drug - Topical Agents	salicylate	LC/MS polar	1515	C00805	HMDB01895	338	2.55	0.0001
4730			sulfate*	LC/MS neg	46960	C00059	HMDB01448	1118	1.35	0.0010
4733			O-sulfo-L-tyrosine	LC/MS neg	45413			514186	1.63	0.0001
4751		Chemical	2-aminophenol sulfate	LC/MS neg	43266		HMDB61116	181670	2.08	0.0031
4828			perfluorooctanesulfonate (PFOS)	LC/MS polar	57564	C18142	HMDB59586	74483	0.21	0.0000
4855			3-hydroxypyridine sulfate	LC/MS neg	48448				5.67	0.0001
4869			thioproline	LC/MS pos early	53231			93176	2.19	0.0039

METABOLOMICS FROM MUSCLE TISSUE

Heat map of statistically significant biochemicals profiled in this study.

Comparison groups significantly different:

- (red) $p \leq 0.05$, group means fold of change ≥ 1.00
- (green) $p \leq 0.05$, group means fold of change < 1.00

Comparison groups trending towards significant difference:

- (pink) $0.05 < p < 0.10$, group means fold of change ≥ 1.00
- (light green) $0.05 < p < 0.10$, group means fold of change < 1.00

Pathway Sort Order	Super Pathway	Sub Pathway	Biochemical Name	Platform	Comp ID	KEGG	HMDB	PUBCHEM	Fold of Change<br>Welch's Two-Sample t-Test<br>All Samples<br>Nephrectomy / Sham	Statistical Values<br>Welch's Two-Sample t-Test<br>All Samples<br>Nephrectomy / Sham<br>p-value
1			glycine	LC/MS pos early	58	C00037	HMDB00123	750	1.40	0.0072
2			N-acetylglycine	LC/MS pos early	27710		HMDB00532	10972	1.94	0.0000
4			sarcosine	LC/MS pos early	1516	C00213	HMDB00271	1088	1.15	0.4140
5			dimethylglycine	LC/MS pos early	5086	C01026	HMDB00092	673	2.31	0.0000
6			betaine	LC/MS pos early	3141	C00719	HMDB00043	247	1.62	0.0002
9		Glycine, Serine and Threonine Metabolism	serine	LC/MS pos early	1648	C00065	HMDB00187	5951	0.94	0.4795
10			N-acetylserine	LC/MS pos early	37076		HMDB02931	65249	0.87	0.0349
15			2-methylserine	LC/MS pos early	53229	C02115		94309	1.92	0.2368
16			threonine	LC/MS pos early	1284	C00188	HMDB00167	6288	0.84	0.0093
17			N-acetylthreonine	LC/MS neg	33939			152204	1.01	0.9373
21			homoserine	LC/MS polar	18351	C00263	HMDB00719	12647	1.25	0.1248
23			homoserine lactone	LC/MS pos early	15696	C02926		445963	0.92	0.4216
28			alanine	LC/MS pos early	1126	C00041	HMDB00161	5950	1.05	0.4619
30			N-acetylalanine	LC/MS polar	1585	C02847	HMDB00766	88064	1.01	0.8780
31			N-methylalanine	LC/MS pos early	37069	C02721	HMDB01906	5288725	1.25	0.5625
34		Alanine and Aspartate Metabolism	aspartate	LC/MS pos early	443	C00049	HMDB00191	5960	1.37	0.4296
35			N-acetylaspartate (NAA)	LC/MS pos early	22185	C01042	HMDB00812	65065	1.69	0.1014
38			asparagine	LC/MS pos early	512	C00152	HMDB00168	6267	0.93	0.7466
39			N-acetylasparagine	LC/MS pos early	33942		HMDB06028	99715	2.78	0.0002
41			glutamate	LC/MS pos early	57	C00025	HMDB00148	611	1.20	0.1306
42			glutamine	LC/MS pos early	53	C00064	HMDB00641	5961	1.13	0.1897
43			alpha-ketoglutaramate*	LC/MS polar	62101				2.77	0.0024
44			N-acetylglutamate	LC/MS polar	15720	C00624	HMDB01138	70914	1.94	0.0099
45			N-acetylglutamine	LC/MS pos early	33943	C02716	HMDB06029	182230	1.42	0.1596
47		Glutamate Metabolism	4-hydroxyglutamate	LC/MS pos early	40499	C03079	HMDB01344	439902	2.29	0.0653
50			glutamate, gamma-methyl ester	LC/MS pos early	33487		HMDB61715	68662	1.47	0.0708
51			pyroglutamine*	LC/MS pos early	46225			134508	5.03	0.0001
52			N-acetyl-aspartyl-glutamate (NAAG)	LC/MS pos early	35665	C12270	HMDB01067	5255	1.90	0.1874
53			beta-citrylglutamate	LC/MS neg	54923	C20775		72715786	2.89	0.1119
56			N-methyl-GABA	LC/MS pos early	39577	C15987		70703	2.21	0.0002
65			histidine	LC/MS neg	59	C00135	HMDB00177	6274	1.32	0.0005
66			1-methylhistidine	LC/MS pos early	30460	C01152	HMDB00001	92105	4.07	0.0005
67			3-methylhistidine	LC/MS neg	15677	C01152	HMDB00479	64969	1.53	0.0046
68			N-acetylhistidine	LC/MS polar	33946	C02997	HMDB32055	75619	2.27	0.0463
69			N-acetyl-3-methylhistidine*	LC/MS neg	43256			193270	3.25	0.1597
70			N-acetyl-1-methylhistidine*	LC/MS pos early	43255			193270	3.13	0.0434

72			trans-urocanate	LC/MS polar	607	C00785	HMDB00301	736715	1.14	0.7902
73			cis-urocanate	LC/MS polar	40410		HMDB34174	1549103	2.01	0.3854
74			imidazole propionate	LC/MS pos early	40730		HMDB02271	70630	8.12	0.0022
75			formiminoglutamate	LC/MS pos early	43493	C00439	HMDB00854	439233	1.70	0.2264
76		Histidine Metabolism	imidazole lactate	LC/MS pos early	15716	C05568	HMDB02320	440129	4.47	0.0010
77			carnosine	LC/MS pos early	1768	C00386	HMDB00033	439224	0.84	0.0216
78			homocarnosine	LC/MS neg	1633	C00884	HMDB00745	10243361	1.31	0.3337
79			N-acetylcarnosine	LC/MS pos early	43488		HMDB12881	9903482	1.01	0.9306
80			anserine	LC/MS pos early	15747	C01262	HMDB00194	112072	0.74	0.0108
81			histamine	LC/MS pos early	1574	C00388	HMDB00870	774	1.61	0.1451
82			1-methylhistamine	LC/MS pos early	43831	C05127	HMDB00898	3614	#DIV/0!	0.3115
83			1-methyl-4-imidazoleacetate	LC/MS pos early	32350	C05828	HMDB02820	75810	8.03	0.0548
84			1-ribosyl-imidazoleacetate*	LC/MS pos early	61868	C05131	HMDB02331	5117448	5.31	0.0001
85			4-imidazoleacetate	LC/MS pos early	32349	C02835	HMDB02024	96215	1.22	0.5489
89			N-acetylhistamine	LC/MS neg	48679	C05135	HMDB13253	69602	2.11	0.1127
91			lysine	LC/MS pos early	1301	C00047	HMDB00182	5962	0.99	0.9481
92			N2-acetyllysine	LC/MS polar	36751	C12989	HMDB00446	92907	2.44	0.0058
93			N6-acetyllysine	LC/MS polar	36752	C02727	HMDB00206	92832	1.45	0.0001
98			N6,N6,N6-trimethyllysine	LC/MS pos early	1498	C03793	HMDB01325	440120	1.01	0.9436
99		Lysine Metabolism	5-hydroxylysine	LC/MS pos early	15685	C16741	HMDB00450	1029	2.45	0.0035
100			5-(galactosylhydroxy)-L-lysine	LC/MS pos early	43582				1.41	0.0109
102			2-aminoadipate	LC/MS polar	6146	C00956	HMDB00510	469	1.97	0.0032
110			pipecolate	LC/MS pos early	1444	C00408	HMDB00070	849	2.69	0.0000
114			5-aminovalerate	LC/MS pos early	18319	C00431	HMDB03355	138	1.30	0.1364
115			N,N,N-trimethyl-5-aminovalerate	LC/MS pos early	57687				1.04	0.8133
116			phenylalanine	LC/MS pos early	64	C00079	HMDB00159	6140	1.26	0.0593
117		Phenylalanine Metabolism	N-acetylphenylalanine	LC/MS neg	33950	C03519	HMDB00512	74839	3.79	0.0005
118			1-carboxyethylphenylalanine	LC/MS neg	62566				2.16	0.0237
122			phenyllactate (PLA)	LC/MS neg	22130	C05607	HMDB00779	3848	3.75	0.1056
132			tyrosine	LC/MS pos early	1299	C00082	HMDB00158	6057	1.04	0.7385
133			N-acetyltyrosine	LC/MS polar	32390		HMDB00866	68310	1.43	0.7934
134			1-carboxyethyltyrosine	LC/MS neg	62564				1.21	0.6981
142			4-hydroxyphenylpyruvate	LC/MS neg	1669	C01179	HMDB00707	979	0.89	0.4160
145			3-(4-hydroxyphenyl)lactate	LC/MS neg	32197	C03672	HMDB00755	9378	1.21	0.0915
149			phenol sulfate	LC/MS neg	32553	C02180	HMDB60015	74426	4.36	0.0120
150		Tyrosine Metabolism	phenol glucuronide	LC/MS neg	52747		HMDB60014	87235	5.11	0.0985
163			3-methoxytyrosine	LC/MS pos early	12017		HMDB01434	1670	1.11	0.4057
176			O-methyltyrosine	LC/MS pos early	37451		HMDB14903	76957	1.55	0.0433
178			gentisate	LC/MS polar	18280	C00628	HMDB00152	3469	1.56	0.4224
185			p-cresol glucuronide*	LC/MS neg	48841		HMDB11686	154035	3.61	0.0307
190			4-hydroxycinnamate sulfate	LC/MS neg	48410			6070438	4.35	0.1164
198			catechol glucuronide	LC/MS neg	53243			75124209	3.11	0.0126
207			tryptophan	LC/MS pos early	54	C00078	HMDB00929	6305	1.10	0.4791
208			N-acetyltryptophan	LC/MS neg	33959	C03137	HMDB13713	700653	4.08	0.1555
214			C-glycosyltryptophan	LC/MS neg	48782			10981970	1.54	0.0038
218			kynurenine	LC/MS pos early	15140	C00328	HMDB00684	161166	1.34	0.6268
220			N-acetylkynurenine (2)	LC/MS neg	48757				2.85	0.0130
222		Tryptophan Metabolism	kynurenate	LC/MS neg	1417	C01717	HMDB00715	3845	1.31	0.6496
231			serotonin	LC/MS pos early	2342	C00780	HMDB00259	5202	1.43	0.1268
238			indolelactate	LC/MS neg	18349	C02043	HMDB00671	92904	2.85	0.0044
239			indoleacetate	LC/MS neg	27513	C00954	HMDB00197	802	1.44	0.4641
241	Amino Acid		indolepropionate	LC/MS neg	32405		HMDB02302	3744	0.60	0.0747
248			indoleacetylglycine	LC/MS neg	54730			446640	4.18	0.0116
251			3-indoxyl sulfate	LC/MS neg	27672		HMDB00682	10258	4.12	0.0246
257			leucine	LC/MS pos early	60			5246661	1.00	0.9825
258			N-acetylleucine	LC/MS neg	1587	C02710	HMDB11756	70912	1.67	0.0767
259			1-carboxyethylleucine	LC/MS neg	62559				1.22	0.3906
261			4-methyl-2-oxopentanoate	LC/MS neg	22116	C00233	HMDB00695	70	0.60	0.0397
264			alpha-hydroxyisocaproate	LC/MS neg	22132	C03264	HMDB00746	83697	1.49	0.0905

268		isovalerylglycine	LC/MS neg	35107		HMDB00678	546304	2.16	0.0102
269		isovalerylcarnitine (C5)	LC/MS pos early	34407		HMDB00688	6426851	1.04	0.8599
276		beta-hydroxyisovalerate	LC/MS polar	12129		HMDB00754	69362	1.38	0.0581
279		3-methylglutaconate	LC/MS polar	57747		HMDB00522	1551553	4.34	0.0095
292		isoleucine	LC/MS pos early	1125	C00407	HMDB00172	6306	0.92	0.2781
294		N-acetylisoleucine	LC/MS neg	33967		HMDB61684	2802421	1.58	0.1144
295		1-carboxyethylisoleucine	LC/MS neg	62558				1.73	0.1204
296		3-methyl-2-oxovalerate	LC/MS neg	15676	C00671	HMDB03736	47	0.64	0.0684
297	Leucine, Isoleucine and Valine Metabolism	alpha-hydroxyisovalerate	LC/MS polar	46537		HMDB00407	99823	2.27	0.0111
299		2-methylbutyrylcarnitine (C5)	LC/MS pos early	45095		HMDB00378	6426901	0.71	0.1879
302		tiglylcarnitine (C5:1-DC)	LC/MS pos early	35428		HMDB02366	22833596	0.57	0.0198
305		3-hydroxy-2-ethylpropionate	LC/MS polar	32397		HMDB00396	188979	1.49	0.0510
307		ethylmalonate	LC/MS polar	15765		HMDB00622	11756	1.87	0.0281
308		methylsuccinate	LC/MS polar	15745		HMDB01844	10349	2.35	0.0036
314		valine	LC/MS pos early	1649	C00183	HMDB00883	6287	0.82	0.0159
315		N-acetylvaline	LC/MS neg	1591		HMDB11757	66789	1.22	0.2246
316		1-carboxyethylvaline	LC/MS neg	62562				1.27	0.1378
318		3-methyl-2-oxobutyrate	LC/MS polar	44526	C00141	HMDB00019	49	0.62	0.0328
319		2-hydroxy-3-methylvalerate	LC/MS neg	36746		HMDB00317	164623	2.18	0.3791
321		isobutyrylcarnitine (C4)	LC/MS pos early	33441		HMDB00736	168379	1.37	0.1622
322		isobutyrylglycine	LC/MS neg	35437		HMDB00730	10855600	1.61	0.2311
323		3-hydroxyisobutyrate	LC/MS polar	1549	C06001	HMDB00336	87	1.99	0.0147
327		methionine	LC/MS pos early	1302	C00073	HMDB00696	6137	1.07	0.3531
328		N-acetylmethionine	LC/MS neg	1589	C02712	HMDB11745	448580	1.21	0.2523
329		N-formylmethionine	LC/MS neg	2829	C03145	HMDB01015	439750	1.21	0.1425
331		methionine sulfone	LC/MS polar	44878			69961	4.60	0.0000
332		methionine sulfoxide	LC/MS pos early	18374	C02989	HMDB02005	158980	1.22	0.3273
338		S-adenosylmethionine (SAM)	LC/MS pos early	15915	C00019	HMDB01185	34756	1.00	0.9412
339		S-adenosylhomocysteine (SAH)	LC/MS neg	42382	C00021	HMDB00939	439155	1.09	0.3929
343		cystathionine	LC/MS pos early	15705	C02291	HMDB00099	439258	2.64	0.0000
345	Methionine, Cysteine, SAM and Taurine Metabolism	cysteine	LC/MS pos early	1868	C00097	HMDB00574	5862	1.38	0.2918
346		N-acetylcysteine	LC/MS neg	1586	C06809	HMDB01890	12035	1.14	0.2890
347		S-methylcysteine	LC/MS pos early	39592		HMDB02108	24417	0.80	0.1066
348		S-methylcysteine sulfoxide	LC/MS pos early	43378		HMDB29432	82142	0.75	0.0883
357		hypotaurine	LC/MS polar	590	C00519	HMDB00965	107812	1.09	0.5714
358		taurine	LC/MS neg	2125	C00245	HMDB00251	1123	1.09	0.2486
359		N-acetyltaurine	LC/MS neg	48187			159864	1.00	0.9908
361		taurocyamine	LC/MS pos early	35117	C01959	HMDB03584	68340	0.84	0.1730
363		3-sulfo-L-alanine	LC/MS polar	47089	C00506	HMDB02757	72886	1.12	0.7946
365		arginine	LC/MS pos early	1638	C00062	HMDB00517	232	0.94	0.7246
366		argininosuccinate	LC/MS pos early	15497	C03406	HMDB00052	828	4.56	0.0592
367		urea	LC/MS pos early	1670	C00086	HMDB00294	1176	2.74	0.0000
369		ornithine	LC/MS pos early	1493	C00077	HMDB03374	6262	1.04	0.7793
372		2-oxoarginine*	LC/MS pos early	55072	C03771	HMDB04225	558	1.10	0.6511
373		citrulline	LC/MS pos early	2132	C00327	HMDB00904	9750	2.57	0.0011
374		homoarginine	LC/MS pos early	22137	C01924	HMDB00670	9085	1.19	0.3788
375		homocitrulline	LC/MS polar	22138	C02427	HMDB00679	65072	3.88	0.0002
376		proline	LC/MS pos early	1898	C00148	HMDB00162	145742	1.00	0.9964
379	Urea cycle; Arginine and Proline Metabolism	dimethylarginine (SDMA + ADMA)	LC/MS pos early	36808	C03626	HMDB01539	123831	2.14	0.0021
380		N-acetylarginine	LC/MS pos early	33953	C02562	HMDB04620	67427	1.27	0.2830
381		N-acetylcitrulline	LC/MS neg	48434	C15532	HMDB00856	656979	5.50	0.0317
383		N-delta-acetylornithine	LC/MS neg	43249			9920500	1.70	0.0000
385		N2,N5-diacetylornithine	LC/MS neg	43591			10398396	2.94	0.0182
388		trans-4-hydroxyproline	LC/MS pos early	32306	C01157	HMDB00725	5810	1.79	0.0001
390		pro-hydroxy-pro	LC/MS pos early	35127		HMDB06695	11673055	3.95	0.0016
393		N-methylproline	LC/MS pos early	37431			557	1.59	0.1050
396		argininate*	LC/MS pos early	57461		HMDB03148	160437	1.60	0.0461
399		guanidinoacetate	LC/MS pos early	43802	C00581	HMDB00128	763	1.73	0.0253
400	Creatine Metabolism	creatine	LC/MS pos early	27718	C00300	HMDB00064	586	0.92	0.0679

401		Creatine Metabolism	creatinine	LC/MS pos early	513	C00791	HMDB00562	588	0.91	0.3037
402			creatine phosphate	LC/MS neg	33951	C02305	HMDB01511	587	0.87	0.7809
407			putrescine	LC/MS pos early	1408	C00134	HMDB01414	1045	1.25	0.4834
409			N-acetyl-isoputreanine*	LC/MS polar	62309				2.98	0.0002
410			spermidine	LC/MS pos early	485	C00315	HMDB01257	1102	1.42	0.1102
414		Polyamine Metabolism	acisoga	LC/MS pos early	43258			129397	6.40	0.0086
418			5-methylthioadenosine (MTA)	LC/MS pos early	1419	C00170	HMDB01173	439176	0.89	0.2332
419			N-acetylputrescine	LC/MS pos early	37496	C02714	HMDB02064	122356	1.71	0.0395
425			(N(1) + N(8))-acetylspermidine	LC/MS pos early	57814				3.38	0.0651
426		Guanidino and Acetamido Metabolism	1-methylguanidine	LC/MS pos early	48114	C02294	HMDB01522	10111	2.37	0.0000
427			4-guanidinobutanoate	LC/MS pos early	15681	C01035	HMDB03464	500	5.34	0.0001
430			glutathione, reduced (GSH)	LC/MS pos early	2127	C00051	HMDB00125	124886	1.28	0.2036
431			glutathione, oxidized (GSSG)	LC/MS pos early	27727	C00127	HMDB03337	65359	1.28	0.1208
433			cysteine-glutathione disulfide	LC/MS pos early	35159		HMDB00656	4247235	1.16	0.7355
434			S-methylglutathione	LC/MS pos early	33944	C11347		3605667	1.37	0.0991
435			S-lactoylglutathione	LC/MS pos early	15731	C03451	HMDB01066	440018	0.91	0.8218
436		Glutathione Metabolism	cysteinylglycine	LC/MS pos early	35637	C01419	HMDB00078	439498	1.53	0.3897
439			5-oxoproline	LC/MS neg	1494	C01879	HMDB00267	7405	1.27	0.1578
440			2-aminobutyrate	LC/MS pos early	42374	C02261	HMDB00650	439691	1.06	0.4717
442			2-hydroxybutyrate/2-hydroxyisobutyrate	LC/MS polar	52281				1.62	0.0089
444			ophthalmate	LC/MS pos early	34592		HMDB05765	7018721	1.06	0.7648
447			4-hydroxy-nonenal-glutathione	LC/MS neg	48487				0.95	0.7323
453			gamma-glutamylglutamate	LC/MS pos early	36738	C05282	HMDB11737	92865	1.08	0.7733
454			gamma-glutamylglutamine	LC/MS pos early	2730	C05283	HMDB11738	150914	1.03	0.9257
455			gamma-glutamylglycine	LC/MS pos early	33949		HMDB11667	165527	1.61	0.2098
456			gamma-glutamylhistidine	LC/MS pos early	18245			7017195	1.84	0.0654
457			gamma-glutamylisoleucine*	LC/MS neg	34457		HMDB11170	14253342	2.43	0.2137
458			gamma-glutamylleucine	LC/MS pos early	18369		HMDB11171	151023	1.39	0.1345
459			gamma-glutamyl-alpha-lysine	LC/MS pos early	55015			65254	1.99	0.0200
460		Gamma-glutamyl Amino Acid	gamma-glutamyl-epsilon-lysine	LC/MS pos early	33934		HMDB03869	7015685	1.73	0.0475
461			gamma-glutamylmethionine	LC/MS pos early	44872		HMDB29155	7009567	1.56	0.4010
462			gamma-glutamylphenylalanine	LC/MS pos early	33422		HMDB00594	111299	2.20	0.1320
463			gamma-glutamylthreonine	LC/MS pos early	33364		HMDB29159	76078708	1.16	0.2715
466			gamma-glutamylvaline	LC/MS pos early	43829		HMDB11172	7015683	1.38	0.1281
468			gamma-glutamylcitrulline*	LC/MS pos early	62104				3.29	0.0075
469			gamma-glutamyl-2-aminobutyrate	LC/MS pos early	37092				1.16	0.2705
480	Peptide		alanylleucine	LC/MS pos early	37093		HMDB28691	259583	1.17	0.6637
612			glycylleucine	LC/MS pos early	34398	C02155	HMDB00759	92843	1.42	0.0593
622			glycylvaline	LC/MS pos early	18357		HMDB28854	97417	1.05	0.7317
646			isoleucylglycine	LC/MS neg	40008		HMDB28907	342532	1.36	0.0523
659			leucylalanine	LC/MS pos early	40010		HMDB28922	259321	1.15	0.6303
664			leucylglycine	LC/MS pos early	40045		HMDB28929	79070	1.14	0.5256
711			phenylalanylalanine	LC/MS neg	41374			5488196	1.16	0.2080
715		Dipeptide	phenylalanylglycine	LC/MS neg	41370		HMDB28995	98207	1.64	0.0539
734			prolylglycine	LC/MS pos early	40703		HMDB11178	6426709	0.93	0.3819
772			threonylphenylalanine	LC/MS neg	31530		HMDB29068	4099799	1.15	0.5546
814			valylglutamine	LC/MS neg	42079		HMDB29125	5253209	1.04	0.9001
815			valylglycine	LC/MS neg	40475		HMDB29127	136487	1.12	0.5729
818			valylleucine	LC/MS pos early	39994		HMDB29131	352039	1.13	0.7672
828			leucylglutamine*	LC/MS pos early	48189		HMDB28927	4305457	1.24	0.4249
897		Acetylated Peptides	phenylacetylglycine	LC/MS neg	33945	C05598	HMDB00821	68144	3.92	0.0381
922			1,5-anhydroglucitol (1,5-AG)	LC/MS neg	20675	C07326	HMDB02712	64960	0.93	0.3583
925			glucose	LC/MS polar	48152	C00031	HMDB00122	79025	0.95	0.3371
926			glucose 6-phosphate	LC/MS polar	31260	C00668	HMDB01401	5958	0.73	0.2147
927			glucose 1-phosphate	LC/MS polar	15561	C00103	HMDB01586	65533	0.79	0.3269
929			fructose-6-phosphate	LC/MS polar	12021	C05345	HMDB00124	69507	0.62	0.2083
931			fructose 1,6-bisphosphate	LC/MS neg	15926	C05378	HMDB01058	172313	0.90	0.8073
934		Glycolysis, Gluconeogenesis, and Pyruvate Metabolism	dihydroxyacetone phosphate (DHAP)	LC/MS pos early	15522	C00111	HMDB01473	668	1.38	0.2004
938			2-phosphoglycerate	LC/MS polar	35629	C00631	HMDB03391	59	1.25	0.4269

939			3-phosphoglycerate	LC/MS neg	1414	C00597	HMDB00807	724	1.07	0.8141
940			phosphoenolpyruvate (PEP)	LC/MS neg	597	C00074	HMDB00263	1005	1.19	0.6654
941			pyruvate	LC/MS polar	48990	C00022	HMDB00243	1060	1.00	0.9911
942			lactate	LC/MS polar	527	C00186	HMDB00190	612	0.91	0.1389
945			glycerate	LC/MS polar	1572	C00258	HMDB00139	752	0.90	0.3299
948			6-phosphogluconate	LC/MS neg	15442	C00345	HMDB01316	91493	1.03	0.7892
949			ribulose 5-phosphate	LC/MS polar	1474	C00199	HMDB00618	439184	1.37	0.1124
950		Pentose Phosphate Pathway	ribose 5-phosphate	LC/MS polar	561	C00117	HMDB01548		0.55	0.0660
951			ribose 1-phosphate	LC/MS polar	1763	C00620	HMDB01489	439236	1.86	0.0011
952			5-phosphoribosyl diphosphate (PRPP)	LC/MS neg	36840	C00119	HMDB00280	7339	1.52	0.3125
953			sedoheptulose-7-phosphate	LC/MS pos early	35649	C05382	HMDB01068	616	1.03	0.9483
959			ribose	LC/MS polar	1471	C00121	HMDB00283	5779	1.44	0.1418
960			ribitol	LC/MS polar	15772	C00474	HMDB00508	6912	1.05	0.5710
961			ribonate	LC/MS polar	27731	C01685	HMDB00867	5460677	1.21	0.1291
963		Pentose Metabolism	xylulose 5-phosphate	LC/MS polar	37285	C00231	HMDB00868	439190	1.49	0.0891
982			arabitol/xylitol	LC/MS polar	48885	C01904		6912	3.19	0.0000
984			arabonate/xylonate	LC/MS polar	48255				3.56	0.0001
985	Carbohydrate		sedoheptulose	LC/MS polar	53237		HMDB03219	5459879	1.52	0.1197
986			ribulonate/xylulonate*	LC/MS polar	61858				1.43	0.0043
990			maltotetraose	LC/MS polar	15910	C02052	HMDB01296	446495	1.21	0.4548
992		Glycogen Metabolism	maltotriose	LC/MS polar	44688	C01835	HMDB01262	439586	1.34	0.2006
995			maltose	LC/MS polar	15586	C00208	HMDB00163	10991489	1.42	0.1483
1017		Disaccharides and Oligosaccharides	sucrose	LC/MS polar	1519	C00089	HMDB00258	5988	1.19	0.7215
1035			fructose	LC/MS polar	48195	C00095	HMDB00660	5984	0.91	0.3659
1040			mannitol/sorbitol	LC/MS polar	46142	C00794	HMDB00247	5780	1.37	0.5222
1041			mannose	LC/MS polar	48153	C00159	HMDB00169	18950	1.08	0.5611
1042		Fructose, Mannose and Galactose Metabolism	mannose-6-phosphate	LC/MS polar	1469	C00275	HMDB01078	439198	0.82	0.2884
1051			galactitol (dulcitol)	LC/MS polar	1117	C01697	HMDB00107	11850	0.47	0.0188
1052			galactose 1-phosphate	LC/MS polar	15706	C00446	HMDB00645	123912	1.92	0.0054
1060			galactonate	LC/MS polar	27719	C00880	HMDB00565	128869	1.14	0.2291
1065			UDP-glucose	LC/MS polar	32344	C00029	HMDB00286	8629	0.90	0.6061
1069			UDP-galactose	LC/MS polar	15860	C00052	HMDB00302	18068	0.85	0.4320
1070		Nucleotide Sugar	UDP-glucuronate	LC/MS polar	2763	C00167	HMDB00935	17473	1.09	0.7326
1076			UDP-N-acetylglucosamine/galactosamine	LC/MS polar	46148				1.36	0.0094
1077			cytidine 5'-monophospho-N-acetylneuraminic acid	LC/MS polar	36831	C00128	HMDB01176	448209	1.67	0.0244
1084			glucosamine-6-phosphate	LC/MS polar	580	C00352	HMDB01254	439217	0.93	0.7747
1088			glucuronate	LC/MS polar	15443	C00191	HMDB00127	444791	1.96	0.0267
1092			N-acetylglucosamine 6-phosphate	LC/MS polar	15107	C00357	HMDB02817	439219	1.39	0.2261
1093		Aminosugar Metabolism	N-acetyl-glucosamine 1-phosphate	LC/MS polar	15741	C04256	HMDB01367	440364	1.37	0.0233
1099			N-acetylneuraminate	LC/MS polar	32377	C00270	HMDB00230	439197	1.26	0.1956
1110			N-acetylglucosaminylasparagine	LC/MS pos early	48149	C04540	HMDB00489	123826	0.86	0.0082
1111			erythronate*	LC/MS polar	42420		HMDB00613	2781043	1.97	0.0002
1113			N-acetylglucosamine/N-acetylgalactosamine	LC/MS pos early	46539		HMDB00215	24139	1.17	0.5400
1114		Advanced Glycation End-product	N6-carboxymethyllysine	LC/MS pos early	36713			123800	0.86	0.3873
1118			citrate	LC/MS neg	1564	C00158	HMDB00094	311	1.99	0.2950
1120			aconitate [cis or trans]	LC/MS neg	46173				2.24	0.1421
1122			isocitrate	LC/MS polar	12110	C00311	HMDB00193	1198	3.17	0.3342
1123			isocitric lactone	LC/MS polar	54724			98259	3.00	0.0021
1124			alpha-ketoglutarate	LC/MS polar	528	C00026	HMDB00208	51	0.90	0.6989
1126		TCA Cycle	succinylcarnitine (C4-DC)	LC/MS pos early	37058		HMDB61717	71464481	0.79	0.2709
1127	Energy		succinate	LC/MS polar	1437	C00042	HMDB00254	1110	1.10	0.7067
1128			fumarate	LC/MS polar	1643	C00122	HMDB00134	444972	1.47	0.0083
1129			malate	LC/MS neg	1303	C00149	HMDB00156	525	1.37	0.0114
1134			tricarballylate	LC/MS polar	15729	C19806	HMDB31193	14925	2.60	0.0177
1139			2-methylcitrate/homocitrate	LC/MS neg	52282				1.90	0.0012
1141		Oxidative Phosphorylation	acetylphosphate	LC/MS polar	15488	C00227	HMDB01494	186	1.15	0.3676
1143			phosphate	LC/MS pos early	42109	C00009	HMDB01429	1061	0.94	0.1322
1145		Fatty Acid Synthesis	malonylcarnitine	LC/MS pos early	37059		HMDB02095	22833583	0.74	0.0781
1146			malonate	LC/MS polar	15872	C00383	HMDB00691	867	1.24	0.2230

1168	Medium Chain Fatty Acid	laurate (12:0)	LC/MS neg	1645	C02679	HMDB00638	3893	1.03	0.8269
1171		myristate (14:0)	LC/MS neg	1365	C06424	HMDB00806	11005	0.94	0.5314
1172		myristoleate (14:1n5)	LC/MS neg	32418	C08322	HMDB02000	5281119	0.95	0.8047
1175		pentadecanoate (15:0)	LC/MS neg	1361	C16537	HMDB00826	13849	1.04	0.7047
1176		palmitate (16:0)	LC/MS neg	1336	C00249	HMDB00220	985	1.05	0.6200
1177		palmitoleate (16:1n7)	LC/MS neg	33447	C08362	HMDB03229	445638	0.84	0.5960
1179		margarate (17:0)	LC/MS neg	1121		HMDB02259	10465	1.02	0.8211
1180		10-heptadecenoate (17:1n7)	LC/MS neg	33971		HMDB60038	5312435	1.00	0.9832
1182	Long Chain Fatty Acid	stearate (18:0)	LC/MS neg	1358	C01530	HMDB00827	5281	1.03	0.6201
1184		oleate/vaccenate (18:1)	LC/MS neg	52285				1.17	0.4590
1191		nonadecanoate (19:0)	LC/MS neg	1356	C16535	HMDB00772	12591	1.00	0.9654
1192		10-nonadecenoate (19:1n9)	LC/MS neg	33972		HMDB13622	5312513	0.94	0.7467
1195		arachidate (20:0)	LC/MS neg	1118	C06425	HMDB02212	10467	0.99	0.9101
1198		eicosenoate (20:1)	LC/MS neg	33587	C16526	HMDB02231	5282768	1.15	0.4719
1202		behenate (22:0)*	LC/MS neg	12125	C08281	HMDB00944	8215	0.89	0.7794
1203		erucate (22:1n9)	LC/MS neg	1552	C08316	HMDB02068	5281116	1.10	0.7494
1210		heneicosapentaenoate (21:5n3)	LC/MS neg	57658			11998573	1.20	0.3988
1211		hexadecadienoate (16:2n6)	LC/MS neg	57652		HMDB00477		1.45	0.1691
1212		hexadecatrienoate (16:3n3)	LC/MS neg	57651			5312428	1.25	0.3422
1215		stearidonate (18:4n3)	LC/MS neg	33969	C16300	HMDB06547	5312508	1.28	0.3900
1216		eicosapentaenoate (EPA; 20:5n3)	LC/MS neg	18467	C06428	HMDB01999	446284	1.25	0.4650
1217		docosapentaenoate (n3 DPA; 22:5n3)	LC/MS neg	32504	C16513	HMDB06528	6441454	1.33	0.1479
1218		docosahexaenoate (DHA; 22:6n3)	LC/MS neg	44675	C06429	HMDB02183	445580	1.27	0.2538
1219		docosatrienoate (22:3n3)	LC/MS neg	32417	C16534	HMDB02823	5312556	2.77	0.0303
1222		nisinate (24:6n3)	LC/MS neg	57810		HMDB02007	11792612	0.89	0.6712
1223	Polyunsaturated Fatty Acid (n3 and n6)	linoleate (18:2n6)	LC/MS neg	1105	C01595	HMDB00673	5280450	1.23	0.4155
1225		linolenate [alpha or gamma; (18:3n3 or 6)]	LC/MS neg	34035	C06426	HMDB03073	5280934	1.16	0.6114
1227		dihomo-linolenate (20:3n3 or n6)	LC/MS neg	35718	C03242	HMDB02925	5280581	1.18	0.4146
1228		arachidonate (20:4n6)	LC/MS neg	1110	C00219	HMDB01043	444899	1.48	0.1533
1229		adrenate (22:4n6)	LC/MS neg	32980	C16527	HMDB02226	5497181	1.13	0.5867
1230		docosapentaenoate (n6 DPA; 22:5n6)	LC/MS neg	37478	C16513	HMDB01976	6441454	1.20	0.4084
1231		docosadienoate (22:2n6)	LC/MS neg	32415	C16533	HMDB61714	5282807	1.35	0.1842
1232		dihomo-linoleate (20:2n6)	LC/MS neg	17805	C16525	HMDB05060	6439848	1.14	0.4722
1234		mead acid (20:3n9)	LC/MS neg	35174		HMDB10378	5312531	1.29	0.2798
1235		docosatrienoate (22:3n6)*	LC/MS neg	57467				1.17	0.5585
1289	Fatty Acid, Branched	15-methylpalmitate (i17:0)	LC/MS neg	38768			17903417	0.90	0.3713
1292		17-methylstearate (i19:0)	LC/MS neg	38296		HMDB37397	3083779	0.83	0.3410
1303		glutarate (C5-DC)	LC/MS polar	396	C00489	HMDB00661	743	1.69	0.0143
1307		2-hydroxyglutarate	LC/MS polar	37253	C02630	HMDB00606	43	1.29	0.0646
1309		adipate (C6-DC)	LC/MS polar	21134	C06104	HMDB00448	196	1.55	0.0437
1311		2-hydroxyadipate	LC/MS polar	31934	C02360	HMDB00321	193530	1.30	0.4266
1312		3-hydroxyadipate*	LC/MS polar	62069		HMDB00345	151913	1.26	0.4084
1322	Fatty Acid, Dicarboxylate	azelate (C9-DC)	LC/MS polar	18362	C08261	HMDB00784	2266	1.36	0.4613
1324		sebacate (C10-DC)	LC/MS polar	32398	C08277	HMDB00792	5192	1.81	0.1291
1326		dodecadienoate (12:2)*	LC/MS neg	62276				1.05	0.7708
1330		hexadecanedioate (C16-DC)	LC/MS neg	35678	C19615	HMDB00672	10459	1.25	0.1357
1331		dodecenedioate (C12:1-DC)*	LC/MS neg	61864				0.91	0.7895
1334		octadecenedioate (C18:1-DC)*	LC/MS neg	61861				1.10	0.7243
1382	Fatty Acid, Amino	2-aminoheptanoate	LC/MS pos early	43761			227939	1.21	0.4727
1398		butyrylcarnitine (C4)	LC/MS pos early	32412	C02862	HMDB02013	439829	1.74	0.0518
1399		butyrylglycine	LC/MS neg	31850		HMDB00808	88412	3.07	0.0065
1401	Fatty Acid Metabolism (also BCAA Metabolism)	propionylcarnitine (C3)	LC/MS pos early	32452	C03017	HMDB00824	107738	1.05	0.7410
1402		propionylglycine	LC/MS neg	31932		HMDB00783	98681	1.51	0.0844
1404		methylmalonate (MMA)	LC/MS polar	1496	C02170	HMDB00202	487	1.04	0.8862
1419	Fatty Acid Metabolism(Acyl Glycine)	N-palmitoylglycine	LC/MS neg	42092		HMDB13034	151008	1.30	0.1111
1428		acetylcarnitine (C2)	LC/MS pos early	32198	C02571	HMDB00201	1	0.89	0.4900
1430		3-hydroxybutyrylcarnitine (1)	LC/MS pos early	43264		HMDB13127	53481617	1.58	0.0807
1431		3-hydroxybutyrylcarnitine (2)	LC/MS pos early	52984		HMDB13127		1.23	0.1612
1433		hexanoylcarnitine (C6)	LC/MS pos early	32328		HMDB00705	6426853	1.35	0.2312

1436	Fatty Acid Metabolism(Acyl Carnitine)	octanoylcarnitine (C8)	LC/MS pos late	33936	C02838	HMDB00791	123701	1.15	0.6196
1438		decanoylcarnitine (C10)	LC/MS pos late	33941		HMDB00651	10245190	1.28	0.4741
1440		5-dodecenoylcarnitine (C12:1)	LC/MS pos late	61769				0.98	0.9123
1441		cis-4-decenoylcarnitine (C10:1)	LC/MS pos late	38178				1.25	0.4004
1442		laurylcarnitine (C12)	LC/MS pos late	34534		HMDB02250	10427569	1.21	0.4616
1443		myristoylcarnitine (C14)	LC/MS pos late	33952		HMDB05066	6426854	1.02	0.9375
1445		palmitoylcarnitine (C16)	LC/MS pos late	44681	C02990	HMDB00222	461	0.86	0.2735
1446		palmitoleoylcarnitine (C16:1)*	LC/MS pos late	53223			71464547	0.91	0.6259
1447		stearoylcarnitine (C18)	LC/MS pos late	34409		HMDB00848	6426855	0.99	0.9696
1448		linoleoylcarnitine (C18:2)*	LC/MS pos late	46223		HMDB06469	6450015	1.08	0.6738
1449		linolenoylcarnitine (C18:3)*	LC/MS pos late	57511				0.81	0.3164
1450		3-hydroxyoleoylcarnitine	LC/MS pos late	61840				1.21	0.6276
1451		oleoylcarnitine (C18:1)	LC/MS pos late	35160		HMDB05065	6441392	0.93	0.6342
1453		myristoleoylcarnitine (C14:1)*	LC/MS pos late	48182			90659872	1.20	0.5139
1463		arachidoylcarnitine (C20)*	LC/MS pos late	57513		HMDB06460		1.73	0.0802
1464		arachidonoylcarnitine (C20:4)	LC/MS pos late	57518				0.96	0.8161
1465		adrenoylcarnitine (C22:4)*	LC/MS pos late	57528				0.88	0.5481
1468		dihomo-linolenoylcarnitine (C20:3n3 or 6)*	LC/MS pos late	57521				0.77	0.1268
1469		dihomo-linoleoylcarnitine (C20:2)*	LC/MS pos late	57520				0.86	0.5305
1470		eicosenoylcarnitine (C20:1)*	LC/MS pos late	57519				1.13	0.6422
1471		erucoylcarnitine (C22:1)*	LC/MS pos late	57525				1.45	0.1427
1473		docosatrienoylcarnitine (C22:3)*	LC/MS pos late	57527				1.03	0.9103
1474		docosapentaenoylcarnitine (C22:5n3)*	LC/MS pos late	57529				0.70	0.2222
1475		docosahexaenoylcarnitine (C22:6)*	LC/MS pos late	57523				0.58	0.1360
1477		lignoceroylcarnitine (C24)*	LC/MS pos late	57515				1.11	0.4763
1478		margaroylcarnitine (C17)*	LC/MS pos late	57512		HMDB06210		0.96	0.8640
1479		nervonoylcarnitine (C24:1)*	LC/MS pos late	57531				1.19	0.4848
1482		pentadecanoylcarnitine (C15)*	LC/MS pos late	57522				1.02	0.9142
1485		3-hydroxypalmitoylcarnitine	LC/MS pos late	61839				1.13	0.7488
1487	Carnitine Metabolism	deoxycarnitine	LC/MS pos early	36747	C01181	HMDB01161	134	1.02	0.8924
1488		carnitine	LC/MS pos early	15500	C00318	HMDB00062	10917	0.73	0.0005
1493	Ketone Bodies	3-hydroxybutyrate (BHBA)	LC/MS polar	542	C01089	HMDB00357	441	2.53	0.0855
1509	Fatty Acid, Monohydroxy	4-hydroxybutyrate (GHB)	LC/MS polar	34585	C00989	HMDB00710	10413	1.39	0.1016
1516		2-hydroxypalmitate	LC/MS neg	35675		HMDB31057	92836	1.07	0.6834
1534		3-hydroxyoleate*	LC/MS neg	61843				1.22	0.4842
1549		13-HODE + 9-HODE	LC/MS neg	37752			43013	0.92	0.6999
1552		14-HDoHE/17-HDoHE	LC/MS neg	53034				1.06	0.8876
1570	Fatty Acid, Dihydroxy	12,13-DiHOME	LC/MS neg	38395	C14829	HMDB04705	10236635	0.80	0.5636
1571		9,10-DiHOME	LC/MS neg	38399	C14828	HMDB04704	9966640	0.56	0.1873
1651	Eicosanoid	12-HETE	LC/MS neg	37536		HMDB06111	5312983	1.63	0.4230
1657		12-HHTrE	LC/MS neg	57393	C20388	HMDB12535	5283141	1.85	0.5179
1664	Endocannabinoid	oleoyl ethanolamide	LC/MS pos late	38102		HMDB02088	5283454	1.00	0.9992
1676		N-oleoyltaurine	LC/MS neg	39732			6437033	1.41	0.0625
1677		N-stearoyltaurine	LC/MS neg	39730			168274	1.09	0.4051
1678		N-palmitoyltaurine	LC/MS neg	39835				1.13	0.3907
1680		N-linoleoyltaurine*	LC/MS neg	61826				1.46	0.0758
1682		linoleoyl ethanolamide	LC/MS neg	52608		HMDB12252	5283446	0.97	0.7874
1698	Inositol Metabolism	myo-inositol	LC/MS polar	1124	C00137	HMDB00211	892	2.05	0.0017
1699		chiro-inositol	LC/MS polar	37112	C19891	HMDB34220		3.50	0.0000
1727	Phospholipid Metabolism	choline	LC/MS pos early	15506	C00114	HMDB00097	305	1.37	0.0161
1728		choline phosphate	LC/MS pos early	34396	C00588	HMDB01565	1014	0.99	0.9270
1729		cytidine 5'-diphosphocholine	LC/MS polar	34418	C00307	HMDB01413	13804	1.17	0.3287
1731		glycerophosphorylcholine (GPC)	LC/MS pos early	15990	C00670	HMDB00086	71920	1.12	0.6228
1733		phosphoethanolamine	LC/MS neg	1600	C00346	HMDB00224	1015	1.32	0.1044
1734		cytidine-5'-diphosphoethanolamine	LC/MS polar	34410	C00570	HMDB01564	123727	1.41	0.0178
1735		glycerophosphoethanolamine	LC/MS polar	37455	C01233	HMDB00114	123874	1.54	0.0718
1737		glycerophosphoinositol*	LC/MS polar	47155			167572	1.82	0.2218
1738		trimethylamine N-oxide	LC/MS pos early	40406	C01104	HMDB00925	1145	5.26	0.0000
1756		1-myristoyl-2-palmitoyl-GPC (14:0/16:0)	LC/MS pos late	19258		HMDB07869	129657	0.92	0.4388

1760			1-myristoyl-2-arachidonoyl-GPC (14:0/20:4)*	LC/MS pos late	53195		HMDB07883		1.06	0.7153
1770			1,2-dipalmitoyl-GPC (16:0/16:0)	LC/MS pos late	19130		HMDB00564	452110	1.00	0.9787
1771			1-palmitoyl-2-palmitoleoyl-GPC (16:0/16:1)*	LC/MS pos late	52470		HMDB07969		0.87	0.1133
1772			1-palmitoyl-2-stearoyl-GPC (16:0/18:0)	LC/MS pos late	52616		HMDB07970		0.85	0.4281
1774			1-palmitoyl-2-oleoyl-GPC (16:0/18:1)	LC/MS pos late	52461		HMDB07972	6436017	1.00	0.9979
1777			1-palmitoyl-2-linoleoyl-GPC (16:0/18:2)	LC/MS pos late	42446		HMDB07973	5287971	0.96	0.6308
1780			1-palmitoyl-2-gamma-linolenoyl-GPC (16:0/18:3n6)*	LC/MS pos late	54812		HMDB07974		0.89	0.3994
1783			1-palmitoyl-2-dihomo-linolenoyl-GPC (16:0/20:3n3 or 6)*	LC/MS pos late	52454				0.72	0.0305
1787			1-palmitoyl-2-arachidonoyl-GPC (16:0/20:4n6)	LC/MS pos late	52462		HMDB07982	10747814	0.98	0.8240
1793		Phosphatidylcholine (PC)	1-palmitoyl-2-docosahexaenoyl-GPC (16:0/22:6)	LC/MS pos late	52610		HMDB07991	6441886	0.98	0.8063
1799			1-palmitoleoyl-2-linolenoyl-GPC (16:1/18:3)*	LC/MS pos late	53180		HMDB08008		1.12	0.4243
1809			1-stearoyl-2-oleoyl-GPC (18:0/18:1)	LC/MS pos late	52438		HMDB08038		1.00	0.9961
1812			1-stearoyl-2-linoleoyl-GPC (18:0/18:2)*	LC/MS pos late	52452		HMDB08039		1.02	0.8409
1820			1-stearoyl-2-arachidonoyl-GPC (18:0/20:4)	LC/MS pos late	42450		HMDB08048	16219824	1.13	0.3902
1826			1-stearoyl-2-docosahexaenoyl-GPC (18:0/22:6)	LC/MS pos late	52611		HMDB08057		1.18	0.4283
1830			1,2-dioleoyl-GPC (18:1/18:1)	LC/MS pos late	52457			10350317	1.05	0.7003
1833			1-oleoyl-2-linoleoyl-GPC (18:1/18:2)*	LC/MS pos late	52453				1.20	0.0243
1843			1-oleoyl-2-docosahexaenoyl-GPC (18:1/22:6)*	LC/MS pos late	52697		HMDB08123		1.10	0.3991
1844			1,2-dilinoleoyl-GPC (18:2/18:2)	LC/MS pos late	52603		HMDB08138	5288075	1.21	0.1370
1845			1-linoleoyl-2-linolenoyl-GPC (18:2/18:3)*	LC/MS pos late	53176		HMDB08141		1.28	0.2020
1848			1-linoleoyl-2-arachidonoyl-GPC (18:2/20:4n6)*	LC/MS pos late	52710		HMDB08147		1.25	0.1117
1890			1-palmitoyl-2-oleoyl-GPE (16:0/18:1)	LC/MS pos late	19263		HMDB05320	5283496	0.98	0.9203
1891			1-palmitoyl-2-linoleoyl-GPE (16:0/18:2)	LC/MS pos late	42449		HMDB05322	9546747	0.92	0.5926
1894			1-palmitoyl-2-arachidonoyl-GPE (16:0/20:4)*	LC/MS pos late	52464		HMDB05323	9546800	0.93	0.5861
1896			1-palmitoyl-2-docosahexaenoyl-GPE (16:0/22:6)*	LC/MS pos late	52465		HMDB05324	9546799	1.11	0.5640
1903			1-stearoyl-2-oleoyl-GPE (18:0/18:1)	LC/MS pos late	42448		HMDB08993		0.96	0.8891
1906			1-stearoyl-2-linoleoyl-GPE (18:0/18:2)*	LC/MS pos late	52446		HMDB08994	9546749	0.87	0.4089
1911		Phosphatidylethanolamine (PE)	1-stearoyl-2-arachidonoyl-GPE (18:0/20:4)	LC/MS pos late	52447		HMDB09003	5289133	1.01	0.9659
1915			1-stearoyl-2-docosahexaenoyl-GPE (18:0/22:6)*	LC/MS pos late	52466		HMDB05334	9546798	0.97	0.8190
1917			1-oleoyl-2-linoleoyl-GPE (18:1/18:2)*	LC/MS pos late	52687		HMDB05349	9546753	1.13	0.5463
1920			1-oleoyl-2-arachidonoyl-GPE (18:1/20:4)*	LC/MS pos late	55041		HMDB09069		1.27	0.2476
1923			1-oleoyl-2-docosahexaenoyl-GPE (18:1/22:6)*	LC/MS pos late	53209				1.31	0.1538
1924			1,2-dilinoleoyl-GPE (18:2/18:2)*	LC/MS pos late	53174		HMDB09093	9546812	0.97	0.8262
1926			1-linoleoyl-2-arachidonoyl-GPE (18:2/20:4)*	LC/MS pos late	53189		HMDB09102		1.11	0.4698
1962			1-stearoyl-2-oleoyl-GPS (18:0/18:1)	LC/MS pos late	19265		HMDB10163	9547087	1.08	0.7872
1963		Phosphatidylserine (PS)	1-stearoyl-2-linoleoyl-GPS (18:0/18:2)	LC/MS polar	53009			9547091	0.79	0.3685
1965			1-stearoyl-2-arachidonoyl-GPS (18:0/20:4)	LC/MS pos late	52235		HMDB12383		1.11	0.5995
1971			1,2-dipalmitoyl-GPG (16:0/16:0)	LC/MS polar	52625		HMDB10570	11846227	0.47	0.0015
1975		Phosphatidylglycerol (PG)	1-palmitoyl-2-oleoyl-GPG (16:0/18:1)	LC/MS polar	52448			5283509	0.68	0.0126
1982	Lipid		1-stearoyl-2-oleoyl-GPG (18:0/18:1)	LC/MS polar	52623				0.64	0.0087
1989			1-palmitoyl-2-oleoyl-GPI (16:0/18:1)*	LC/MS polar	52669		HMDB09783		0.96	0.7378
1991			1-palmitoyl-2-linoleoyl-GPI (16:0/18:2)	LC/MS polar	52450		HMDB09784		0.94	0.8199
1993		Phosphatidylinositol (PI)	1-palmitoyl-2-arachidonoyl-GPI (16:0/20:4)*	LC/MS polar	52467		HMDB09789		0.96	0.8621
1997			1-stearoyl-2-linoleoyl-GPI (18:0/18:2)	LC/MS pos late	52468		HMDB09809		1.44	0.1385
2008			1-stearoyl-2-arachidonoyl-GPI (18:0/20:4)	LC/MS pos late	52449		HMDB09815		1.04	0.8250
2013			1-oleoyl-2-arachidonoyl-GPI (18:1/20:4) *	LC/MS pos late	54994		HMDB09844		1.21	0.4709
2031			1-palmitoyl-GPA (16:0)	LC/MS pos late	34428	C04036	HMDB00327	6419701	1.10	0.7769
2045			1-palmitoyl-GPC (16:0)	LC/MS pos late	33955		HMDB10382	86554	1.05	0.6815
2046			2-palmitoyl-GPC (16:0)*	LC/MS neg	35253		HMDB61702	15061532	1.14	0.1939
2047			1-palmitoleoyl-GPC (16:1)*	LC/MS pos late	33230		HMDB10383	24779461	1.12	0.4761
2053			1-stearoyl-GPC (18:0)	LC/MS pos late	33961		HMDB10384	497299	1.28	0.1001
2055			1-oleoyl-GPC (18:1)	LC/MS pos late	48258		HMDB02815	16081932	1.25	0.0675
2058			1-linoleoyl-GPC (18:2)	LC/MS pos late	34419	C04100	HMDB10386	11988421	1.31	0.2137
2060			1-linolenoyl-GPC (18:3)*	LC/MS neg	45951		HMDB10388		1.32	0.4673
2075			1-arachidonoyl-GPC (20:4n6)*	LC/MS neg	34061	C05208	HMDB10395		1.46	0.0033
2093			1-palmitoyl-GPE (16:0)	LC/MS pos late	35631		HMDB11503	9547069	1.25	0.2810
2100			1-stearoyl-GPE (18:0)	LC/MS pos late	42398		HMDB11130	9547068	1.18	0.2800
2101			2-stearoyl-GPE (18:0)*	LC/MS neg	41220		HMDB11129		1.19	0.3978
2102			1-oleoyl-GPE (18:1)	LC/MS neg	35628		HMDB11506	9547071	1.40	0.2943

2104		Lysophospholipid	1-linoleoyl-GPE (18:2)*	LC/MS neg	32635		HMDB11507	52925130	1.33	0.1971
2115			1-arachidonoyl-GPE (20:4n6)*	LC/MS neg	35186		HMDB11517	42607465	1.53	0.0449
2125			1-stearoyl-GPS (18:0)*	LC/MS neg	45966			9547101	1.27	0.4362
2126			1-oleoyl-GPS (18:1)	LC/MS pos late	19260		HMDB61694	9547099	1.10	0.5924
2128			1-linoleoyl-GPS (18:2)*	LC/MS neg	43676				1.46	0.5542
2131			1-palmitoyl-GPG (16:0)*	LC/MS neg	45970			3300276	0.81	0.1508
2133			1-stearoyl-GPG (18:0)	LC/MS neg	34437				0.46	0.0006
2135			1-oleoyl-GPG (18:1)*	LC/MS neg	45968				0.73	0.0276
2137			1-linoleoyl-GPG (18:2)*	LC/MS neg	54885				1.24	0.0371
2138			1-palmitoyl-GPI (16:0)	LC/MS neg	35305		HMDB61695		1.46	0.1130
2141			1-stearoyl-GPI (18:0)	LC/MS neg	19324		HMDB61696		1.09	0.5762
2143			1-oleoyl-GPI (18:1)*	LC/MS neg	36602				1.36	0.2222
2145			1-linoleoyl-GPI (18:2)*	LC/MS neg	36594				1.71	0.0037
2149			1-arachidonoyl-GPI (20:4)*	LC/MS neg	34214		HMDB61690		1.44	0.0032
2240			1-(1-enyl-palmitoyl)-2-oleoyl-GPE (P-16:0/18:1)*	LC/MS pos late	52477		HMDB11342		0.94	0.7160
2241			1-(1-enyl-palmitoyl)-2-linoleoyl-GPE (P-16:0/18:2)*	LC/MS pos late	52677		HMDB11343		0.94	0.5943
2242			1-(1-enyl-palmitoyl)-2-palmitoyl-GPC (P-16:0/16:0)*	LC/MS pos late	52716		HMDB11206	11146967	0.60	0.0581
2243			1-(1-enyl-palmitoyl)-2-palmitoleoyl-GPC (P-16:0/16:1)*	LC/MS pos late	52713		HMDB11207		0.48	0.0250
2244		Plasmalogen	1-(1-enyl-palmitoyl)-2-arachidonoyl-GPE (P-16:0/20:4)*	LC/MS pos late	52673		HMDB11352		1.01	0.9691
2246			1-(1-enyl-palmitoyl)-2-oleoyl-GPC (P-16:0/18:1)*	LC/MS pos late	52478				0.50	0.0856
2252			1-(1-enyl-palmitoyl)-2-arachidonoyl-GPC (P-16:0/20:4)*	LC/MS pos late	52689		HMDB11220		0.85	0.2906
2253			1-(1-enyl-palmitoyl)-2-linoleoyl-GPC (P-16:0/18:2)*	LC/MS pos late	52682		HMDB11211		0.60	0.0335
2257			1-(1-enyl-stearoyl)-2-arachidonoyl-GPE (P-18:0/20:4)*	LC/MS pos late	52475		HMDB05779	9547058	0.97	0.8716
2271			1-(1-enyl-palmitoyl)-GPC (P-16:0)*	LC/MS pos late	52474		HMDB10407	10917802	0.72	0.0068
2272		Lysoplasmalogen	1-(1-enyl-palmitoyl)-GPE (P-16:0)*	LC/MS pos late	39270				1.14	0.3033
2274			1-(1-enyl-oleoyl)-GPE (P-18:1)*	LC/MS pos late	44621				1.11	0.4328
2276			1-(1-enyl-stearoyl)-GPE (P-18:0)*	LC/MS pos late	39271				0.94	0.6935
2279			glycerol	LC/MS neg	15122	C00116	HMDB00131	753	1.17	0.3098
2280		Glycerolipid Metabolism	glycerol 3-phosphate	LC/MS polar	43847	C00093	HMDB00126	754	1.16	0.7511
2285			glycerophosphoglycerol	LC/MS polar	48857	C03274		439964	0.67	0.0039
2286			1-myristoylglycerol (14:0)	LC/MS neg	35625	C01885	HMDB11561	79050	1.42	0.0935
2288			1-pentadecanoylglycerol (15:0)	LC/MS neg	47898			190750	1.11	0.5365
2289			1-palmitoylglycerol (16:0)	LC/MS neg	21127		HMDB31074	14900	2.35	0.1114
2290			1-palmitoleoylglycerol (16:1)*	LC/MS neg	52431		HMDB11565		1.32	0.2620
2293			1-oleoylglycerol (18:1)	LC/MS neg	21184		HMDB11567	5283468	1.42	0.2879
2294			1-linoleoylglycerol (18:2)	LC/MS neg	27447			5283469	1.44	0.0840
2295			1-linolenoylglycerol (18:3)	LC/MS neg	34393		HMDB11569	53480978	1.44	0.0376
2299			1-dihomo-linolenylglycerol (20:3)	LC/MS neg	48341				1.94	0.0199
2300			1-arachidonylglycerol (20:4)	LC/MS neg	34397	C13857	HMDB11549	5282281	2.68	0.0002
2303		Monoacylglycerol	1-docosahexaenoylglycerol (22:6)	LC/MS neg	35153		HMDB11587		2.09	0.0026
2304			2-myristoylglycerol (14:0)	LC/MS neg	34383		HMDB11530	137938	1.68	0.0923
2305			2-palmitoylglycerol (16:0)	LC/MS neg	33419		HMDB11533	123409	1.49	0.2933
2306			2-palmitoleoylglycerol (16:1)*	LC/MS neg	52432		HMDB11565		1.41	0.2754
2308			2-oleoylglycerol (18:1)	LC/MS neg	21232		HMDB11537	5319879	1.48	0.1785
2309			2-linoleoylglycerol (18:2)	LC/MS neg	32506		HMDB11538	5365676	1.32	0.2480
2310			2-arachidonoylglycerol (20:4)	LC/MS neg	19266	C13856	HMDB04666	5282280	1.96	0.1005
2312			2-docosahexaenoylglycerol (22:6)*	LC/MS neg	48675		HMDB11557		1.96	0.0201
2314			1-heptadecenoylglycerol (17:1)*	LC/MS neg	62449				1.32	0.4134
2318			1-dihomo-linoleoylglycerol (20:2)	LC/MS neg	35103				1.27	0.4504
2323			diacylglycerol (16:1/18:2 [2], 16:0/18:3 [1])*	LC/MS pos late	54966				0.69	0.1560
2339			palmitoyl-oleoyl-glycerol (16:0/18:1) [2]*	LC/MS pos late	54942	C13861	HMDB07102		1.11	0.6365
2340			palmitoyl-linoleoyl-glycerol (16:0/18:2) [1]*	LC/MS pos late	52633		HMDB07103	9543695	1.42	0.2570
2341			palmitoyl-linoleoyl-glycerol (16:0/18:2) [2]*	LC/MS pos late	52634		HMDB07103		1.08	0.6975
2342			palmitoyl-linolenoyl-glycerol (16:0/18:3) [2]*	LC/MS pos late	54965		HMDB07105		1.02	0.9072
2346			palmitoyl-dihomo-linolenoyl-glycerol (16:0/20:3n3 or 6) [2	LC/MS pos late	54941				1.43	0.3669
2347			palmitoyl-arachidonoyl-glycerol (16:0/20:4) [1]*	LC/MS pos late	54957		HMDB07112		1.76	0.3339
2348			palmitoyl-arachidonoyl-glycerol (16:0/20:4) [2]*	LC/MS pos late	54958		HMDB07112		1.37	0.2466
2350			palmitoyl-docosahexaenoyl-glycerol (16:0/22:6) [1]*	LC/MS pos late	57373		HMDB07121		1.76	0.0586
2351			palmitoyl-docosahexaenoyl-glycerol (16:0/22:6) [2]*	LC/MS pos late	57374		HMDB07121		1.34	0.2522

2354	Diacylglycerol	stearoyl-linoleoyl-glycerol (18:0/18:2) [2]*	LC/MS pos late	54947				1.29	0.3873
2357		oleoyl-oleoyl-glycerol (18:1/18:1) [2]*	LC/MS pos late	54946		HMDB07218		1.19	0.3987
2359		oleoyl-linoleoyl-glycerol (18:1/18:2) [1]	LC/MS pos late	46798		HMDB07219		1.48	0.1025
2360		oleoyl-linoleoyl-glycerol (18:1/18:2) [2]	LC/MS pos late	46799		HMDB07219		1.12	0.5933
2362		oleoyl-linolenoyl-glycerol (18:1/18:3) [2]*	LC/MS pos late	54970		HMDB07220		1.10	0.6649
2364		linoleoyl-linoleoyl-glycerol (18:2/18:2) [1]*	LC/MS pos late	54968		HMDB07248		1.66	0.1503
2365		linoleoyl-linoleoyl-glycerol (18:2/18:2) [2]*	LC/MS pos late	54969		HMDB07248		1.47	0.1703
2366		linoleoyl-linolenoyl-glycerol (18:2/18:3) [1]*	LC/MS pos late	54963		HMDB07249		1.28	0.5115
2367		linoleoyl-linolenoyl-glycerol (18:2/18:3) [2]*	LC/MS pos late	54964		HMDB07250		0.92	0.6235
2370		stearoyl-arachidonoyl-glycerol (18:0/20:4) [1]*	LC/MS pos late	57450				2.78	0.0465
2371		stearoyl-arachidonoyl-glycerol (18:0/20:4) [2]*	LC/MS pos late	57449				1.63	0.1470
2373		oleoyl-arachidonoyl-glycerol (18:1/20:4) [2]*	LC/MS pos late	54961		HMDB07228		1.29	0.2926
2374		linoleoyl-arachidonoyl-glycerol (18:2/20:4) [1]*	LC/MS pos late	54955		HMDB07257		1.82	0.0136
2375		linoleoyl-arachidonoyl-glycerol (18:2/20:4) [2]*	LC/MS pos late	54956		HMDB07257		1.58	0.0953
2376		stearoyl-docosahexaenoyl-glycerol (18:0/22:6) [1]*	LC/MS pos late	57387				1.30	0.4481
2377		stearoyl-docosahexaenoyl-glycerol (18:0/22:6) [2]*	LC/MS pos late	57368				1.32	0.3904
2378		linoleoyl-docosahexaenoyl-glycerol (18:2/22:6) [1]*	LC/MS pos late	54949				2.40	0.1071
2379		linoleoyl-docosahexaenoyl-glycerol (18:2/22:6) [2]*	LC/MS pos late	54950		HMDB07266		1.32	0.2123
2402	Sphingolipid Synthesis	sphinganine	LC/MS pos late	17769	C00836	HMDB00269	3126	1.18	0.4945
2404		sphingadienine	LC/MS pos late	57426			6449795	1.06	0.6998
2405		phytosphingosine	LC/MS pos late	1510	C12144	HMDB04610	122121	1.08	0.6376
2408	Dihydroceramides	N-palmitoyl-sphinganine (d18:0/16:0)	LC/MS pos late	52604		HMDB11760	5283572	0.52	0.0153
2409		N-stearoyl-sphinganine (d18:0/18:0)*	LC/MS pos late	1759			5283573	0.69	0.2191
2416	Ceramides	N-palmitoyl-sphingosine (d18:1/16:0)	LC/MS pos late	44877		HMDB04949	5283564	1.19	0.5887
2419		N-stearoyl-sphingosine (d18:1/18:0)*	LC/MS pos late	54979		HMDB04950	5283565	0.94	0.6764
2425		N-palmitoyl-sphingadienine (d18:2/16:0)*	LC/MS pos late	57416				0.92	0.8467
2426		N-stearoyl-sphingadienine (d18:2/18:0)*	LC/MS pos late	57417				0.91	0.5381
2441		ceramide (d18:1/14:0, d16:1/16:0)*	LC/MS pos late	57432				1.15	0.6090
2442		ceramide (d18:1/17:0, d17:1/18:0)*	LC/MS pos late	57434				1.15	0.5214
2444		ceramide (d18:1/20:0, d16:1/22:0, d20:1/18:0)*	LC/MS pos late	57440				1.41	0.1786
2446		ceramide (d18:2/24:1, d18:1/24:2)*	LC/MS pos late	57443				1.17	0.6319
2450	Hexosylceramides (HCER)	glycosyl-N-palmitoyl-sphingosine (d18:1/16:0)	LC/MS pos late	53013				1.49	0.1304
2451		glycosyl-N-stearoyl-sphingosine (d18:1/18:0)	LC/MS pos late	52234				1.18	0.4782
2489	Dihydrosphingomyelins	palmitoyl dihydrosphingomyelin (d18:0/16:0)*	LC/MS pos late	52434			9939965	1.09	0.7193
2491		sphingomyelin (d18:0/18:0, d19:0/17:0)*	LC/MS pos late	57473		HMDB12087		1.16	0.4559
2493	Sphingomyelins	palmitoyl sphingomyelin (d18:1/16:0)	LC/MS pos late	37506			9939941	1.08	0.5786
2494		stearoyl sphingomyelin (d18:1/18:0)	LC/MS pos late	19503	C00550	HMDB01348	6453725	0.94	0.5858
2495		behenoyl sphingomyelin (d18:1/22:0)*	LC/MS pos late	48492		HMDB12103		1.07	0.8304
2496		tricosanoyl sphingomyelin (d18:1/23:0)*	LC/MS pos late	52436		HMDB12105		1.00	0.9902
2497		lignoceroyl sphingomyelin (d18:1/24:0)	LC/MS pos late	57330				0.91	0.6472
2500		sphingomyelin (d18:2/24:2)*	LC/MS pos late	57479				0.61	0.0712
2502		sphingomyelin (d18:1/14:0, d16:1/16:0)*	LC/MS pos late	42463		HMDB12097	11433862	1.08	0.6632
2504		sphingomyelin (d17:1/16:0, d18:1/15:0, d16:1/17:0)*	LC/MS pos late	52433				0.95	0.8000
2506		sphingomyelin (d18:2/16:0, d18:1/16:1)*	LC/MS pos late	42459				1.15	0.4531
2507		sphingomyelin (d18:1/17:0, d17:1/18:0, d19:1/16:0)	LC/MS pos late	52615				1.10	0.5627
2508		sphingomyelin (d18:1/18:1, d18:2/18:0)	LC/MS pos late	37529		HMDB12101	6443882	0.95	0.6421
2509		sphingomyelin (d18:1/19:0, d19:1/18:0)*	LC/MS pos late	57475				1.13	0.5326
2510		sphingomyelin (d18:1/20:0, d16:1/22:0)*	LC/MS pos late	48490		HMDB12102		1.18	0.4102
2511		sphingomyelin (d18:1/20:1, d18:2/20:0)*	LC/MS pos late	48491				1.19	0.5948
2515		sphingomyelin (d18:1/22:1, d18:2/22:0, d16:1/24:1)*	LC/MS pos late	48493		HMDB12104		0.99	0.9680
2517		sphingomyelin (d18:2/23:0, d18:1/23:1, d17:1/24:1)*	LC/MS pos late	52435				0.56	0.2223
2518		sphingomyelin (d18:1/24:1, d18:2/24:0)*	LC/MS pos late	47153		HMDB12107		1.04	0.8748
2519		sphingomyelin (d18:2/24:1, d18:1/24:2)*	LC/MS pos late	52437				1.13	0.6353
2521	Sphingosines	sphingosine	LC/MS pos late	17747	C00319	HMDB00252	5353955	1.23	0.3244
2529		hexadecasphingosine (d16:1)*	LC/MS pos late	57428				1.26	0.4307
2537	Mevalonate Metabolism	3-hydroxy-3-methylglutarate	LC/MS polar	531	C03761	HMDB00355	1662	2.21	0.0073
2552	Sterol	cholesterol	LC/MS pos late	63	C00187	HMDB00067	11025495	1.19	0.2879
2563		7-alpha-hydroxy-3-oxo-4-cholestenoate (7-Hoca)	LC/MS neg	36776	C17337	HMDB12458	3081085	1.76	0.1834
2576		4-cholesten-3-one	LC/MS pos late	38125	C00599	HMDB00921	91477	0.99	0.9247

2650		Corticosteroids	corticosterone	LC/MS neg	5983	C02140	HMDB01547	5753	0.93	0.7475
2745			cholate	LC/MS neg	22842	C00695	HMDB00619	221493	2.14	0.1492
2746			glycocholate	LC/MS neg	18476	C01921	HMDB00138	10140	2.17	0.1593
2747			taurocholate	LC/MS neg	18497	C05122	HMDB00036	6675	1.57	0.4759
2748			chenodeoxycholate	LC/MS neg	1563	C02528	HMDB00518	10133	2.42	0.2044
2749			glycochenodeoxycholate	LC/MS neg	32346	C05466	HMDB00637	12544	2.00	0.2529
2750		Primary Bile Acid Metabolism	taurochenodeoxycholate	LC/MS neg	18494	C05465	HMDB00951	387316	1.52	0.3854
2751			beta-muricholate	LC/MS neg	31885	C17726	HMDB00415	5283853	1.78	0.4367
2752			alpha-muricholate	LC/MS neg	34092	C17647	HMDB00506	3083761	2.76	0.1726
2753			tauro-alpha-muricholate	LC/MS neg	42605				1.50	0.3837
2754			tauro-beta-muricholate	LC/MS neg	33983		HMDB00932	168408	1.45	0.5422
2756			glyco-alpha-muricholate**	LC/MS neg	62065				2.04	0.3680
2757			glyco-beta-muricholate**	LC/MS neg	62066				0.96	0.9282
2765			deoxycholate	LC/MS neg	1114	C04483	HMDB00626	222528	2.19	0.1394
2768			taurodeoxycholate	LC/MS neg	12261	C05463	HMDB00896	2733768	1.62	0.4489
2779			ursodeoxycholate	LC/MS neg	1605	C07880	HMDB00946	31401	1.93	0.4219
2784			tauroursodeoxycholate	LC/MS neg	39378		HMDB00874	9848818	1.55	0.4010
2788		Secondary Bile Acid Metabolism	6-oxolithocholate	LC/MS neg	46336			164960	3.18	0.5782
2793			hyodeoxycholate	LC/MS neg	27531	C15517	HMDB00733	5283820	3.55	0.1084
2794			taurohyodeoxycholic acid	LC/MS neg	43588			119046	1.72	0.3861
2795			glycohyodeoxycholate	LC/MS neg	43501			114611	3.01	0.2398
2802			7-ketodeoxycholate	LC/MS neg	31904		HMDB00391	188292	2.70	0.3934
3372			AICA ribonucleotide	LC/MS pos early	38325	C04677	HMDB01517	65110	1.11	0.5976
3373			inosine 5'-triphosphate	LC/MS neg	36862	C00081	HMDB00189	8583	0.72	0.4293
3375			inosine 5'-monophosphate (IMP)	LC/MS pos early	2133	C00130	HMDB00175	8582	0.91	0.1249
3376			inosine	LC/MS neg	1123	C00294	HMDB00195	6021	1.54	0.0020
3377			hypoxanthine	LC/MS neg	3127	C00262	HMDB00157	790	1.82	0.0160
3378		Purine Metabolism, (Hypo)Xanthine/Inosine containing	xanthine	LC/MS neg	3147	C00385	HMDB00292	1188	1.40	0.0258
3380			xanthosine	LC/MS polar	15136	C01762	HMDB00299	64959	2.08	0.0902
3384			2'-deoxyinosine	LC/MS neg	15076	C05512	HMDB00071	65058	1.07	0.8322
3386			urate	LC/MS neg	1604	C00366	HMDB00289	1175	1.34	0.1616
3388			allantoin	LC/MS polar	1107	C02350	HMDB00462	204	3.04	0.0000
3389			allantoic acid	LC/MS polar	33434	C00499	HMDB01209	203	#DIV/0!	0.0000
3393			adenosine 5'-diphosphate (ADP)	LC/MS neg	3108	C00008	HMDB01341	6022	0.97	0.9200
3394			adenosine 5'-monophosphate (AMP)	LC/MS pos early	32342	C00020	HMDB00045	6083	1.17	0.5346
3395			adenosine 3'-monophosphate (3'-AMP)	LC/MS neg	35142	C01367	HMDB03540	41211	2.12	0.0806
3397			adenosine 3',5'-cyclic monophosphate (cAMP)	LC/MS neg	2831	C00575	HMDB00058	6076	1.23	0.2563
3399			adenosine 3',5'-diphosphate	LC/MS neg	15691	C00054	HMDB00061	159296	1.60	0.0709
3400		Purine Metabolism, Adenine containing	adenylosuccinate	LC/MS neg	18360	C03794	HMDB00536	195	1.17	0.5428
3401			adenosine	LC/MS pos early	555	C00212	HMDB00050	60961	2.64	0.2548
3402			adenine	LC/MS neg	554	C00147	HMDB00034	190	1.08	0.4199
3407			N1-methyladenosine	LC/MS neg	15650	C02494	HMDB03331	27476	2.66	0.0319
3417			N6-carbamoylthreonyladenosine	LC/MS neg	35157		HMDB41623	161466	5.93	0.0223
3426			N6-succinyladenosine	LC/MS neg	48130		HMDB00912	165243	2.16	0.0237
3429			guanosine 5'- monophosphate (5'-GMP)	LC/MS pos early	2849	C00144	HMDB01397	6804	1.15	0.3595
3434		Purine Metabolism, Guanine containing	guanosine	LC/MS neg	1573	C00387	HMDB00133	6802	1.85	0.0179
3437			7-methylguanine	LC/MS neg	35114	C02242	HMDB00897	11361	3.93	0.0000
3455	Nucleotide		dihydroorotate	LC/MS polar	601	C00337	HMDB03349	648	2.43	0.0539
3456		Pyrimidine Metabolism, Orotate containing	orotate	LC/MS polar	1505	C00295	HMDB00226	967	1.64	0.0931
3458			orotidine	LC/MS polar	35172		HMDB00788	92751	1.34	0.0137
3461			uridine 5'-triphosphate (UTP)	LC/MS neg	33448	C00075	HMDB00285	6133	1.00	0.9919
3462			uridine 5'-diphosphate (UDP)	LC/MS neg	5345	C00015	HMDB00295	6031	0.83	0.5586
3463			uridine 5'-monophosphate (UMP)	LC/MS pos early	2856	C00105	HMDB00288	6030	1.05	0.8322
3468			uridine	LC/MS neg	606	C00299	HMDB00296	6029	1.43	0.0170
3469		Pyrimidine Metabolism, Uracil containing	uracil	LC/MS pos early	605	C00106	HMDB00300	1174	1.48	0.0592
3470			pseudouridine	LC/MS neg	33442	C02067	HMDB00767	15047	4.70	0.0000
3471			5,6-dihydrouridine	LC/MS neg	61833			94312	3.95	0.0000
3484			2'-deoxyuridine	LC/MS polar	1412	C00526	HMDB00012	13712	1.08	0.4693
3487			3-ureidopropionate	LC/MS pos early	3155	C02642	HMDB00026	111	2.54	0.0084

3488			beta-alanine	LC/MS pos early	55	C00099	HMDB00056	239	0.88	0.0172
3492		Pyrimidine Metabolism, Cytidine containing	cytidine triphosphate	LC/MS neg	2844	C00063	HMDB00082	6176	0.94	0.8266
3493			cytidine diphosphate	LC/MS neg	2841	C00112	HMDB01546	6132	0.83	0.5455
3494			cytidine 5'-monophosphate (5'-CMP)	LC/MS pos early	2372	C00055	HMDB00095	6131	0.91	0.5790
3498			cytidine	LC/MS neg	514	C00475	HMDB00089	6175	1.53	0.0008
3499			cytosine	LC/MS pos early	573	C00380	HMDB00630	597	1.42	0.1961
3500			3-methylcytidine	LC/MS pos early	35132			159649	5.02	0.0312
3501			5-methylcytidine	LC/MS pos early	22119		HMDB00982	92918	0.91	0.1027
3502			N4-acetylcytidine	LC/MS neg	35130		HMDB05923	107461	3.57	0.0000
3508			2'-deoxycytidine	LC/MS neg	15949	C00881	HMDB00014	13711	0.83	0.0503
3509			2'-O-methylcytidine	LC/MS pos early	57554			150971	2.37	0.1107
3510			5-methyl-2'-deoxycytidine	LC/MS pos early	38159	C03592	HMDB02224	440055	1.68	0.0000
3519		Pyrimidine Metabolism, Thymine containing	thymidine	LC/MS neg	2183	C00214	HMDB00273	5789	1.60	0.0096
3520			thymine	LC/MS neg	604	C00178	HMDB00262	1135	1.72	0.0149
3524		Purine and Pyrimidine Metabolism	methylphosphate	LC/MS neg	37070		HMDB61711	13130	1.12	0.2541
3539	Cofactors and Vitamins	Nicotinate and Nicotinamide Metabolism	quinolinate	LC/MS polar	1899	C03722	HMDB00232	1066	5.92	0.0009
3543			nicotinamide	LC/MS pos early	594	C00153	HMDB01406	936	1.16	0.0475
3545			nicotinamide ribonucleotide (NMN)	LC/MS pos early	22152	C00455	HMDB00229	14180	0.79	0.1372
3546			nicotinamide riboside	LC/MS pos early	33013	C03150	HMDB00855	439924	1.74	0.2391
3547			nicotinamide adenine dinucleotide (NAD+)	LC/MS pos early	5278	C00003	HMDB00902	5893	0.64	0.1040
3548			nicotinamide adenine dinucleotide reduced (NADH)	LC/MS neg	31475	C00004	HMDB01487	439153	0.89	0.7127
3554			nicotinamide N-oxide	LC/MS pos early	39582		HMDB02730	72661	3.70	0.0100
3555			1-methylnicotinamide	LC/MS pos early	27665	C02918	HMDB00699	10129985	3.19	0.0011
3560			trigonelline (N'-methylnicotinate)	LC/MS pos early	32401	C01004	HMDB00875	5570	3.23	0.0000
3562			N1-Methyl-2-pyridone-5-carboxamide	LC/MS neg	40469	C05842	HMDB04193	69698	5.76	0.0000
3563			N1-Methyl-4-pyridone-3-carboxamide	LC/MS pos early	57584	C05843	HMDB04194	440810	2.77	0.0000
3564			adenosine 5'-diphosphoribose (ADP-ribose)	LC/MS neg	558	C00301	HMDB01178	192	1.13	0.2617
3567		Riboflavin Metabolism	riboflavin (Vitamin B2)	LC/MS neg	1827	C00255	HMDB00244	493570	1.77	0.0021
3568			flavin adenine dinucleotide (FAD)	LC/MS neg	2134	C00016	HMDB01248	643975	1.33	0.0920
3569			flavin mononucleotide (FMN)	LC/MS neg	15797	C00061	HMDB01520	710	1.15	0.4031
3570		Pantothenate and CoA Metabolism	pantothenate	LC/MS neg	1508	C00864	HMDB00210	6613	1.25	0.1595
3572			phosphopantetheine	LC/MS neg	15504	C01134	HMDB01416	987	1.41	0.0431
3575			coenzyme A	LC/MS neg	46322	C00010	HMDB01423	317	1.44	0.4947
3577			pantetheine	LC/MS neg	57555	C00831		439322	1.88	0.0335
3581		Ascorbate and Aldarate Metabolism	ascorbate (Vitamin C)	LC/MS pos early	32354	C00072	HMDB00044		1.30	0.5053
3582			dehydroascorbate	LC/MS neg	34302	C05422	HMDB01264	835	1.24	0.0603
3583			threonate	LC/MS polar	27738	C01620	HMDB00943	151152	1.04	0.8453
3586			oxalate (ethanedioate)	LC/MS neg	20694	C00209	HMDB02329	971	1.13	0.3788
3587			gulonate*	LC/MS polar	46957	C00257	HMDB03290	9794176	1.73	0.0912
3589		Tocopherol Metabolism	alpha-tocopherol	LC/MS pos late	1561	C02477	HMDB01893	14985	1.45	0.0888
3595			alpha-tocotrienol	LC/MS pos late	37502	C14153	HMDB06327	5282347	1.19	0.4970
3608			gamma-CEHC sulfate*	LC/MS neg	62280				4.86	0.0033
3611			gamma-tocopherol/beta-tocopherol	LC/MS pos late	52473				1.05	0.8560
3618		Tetrahydrobiopterin Metabolism	biopterin	LC/MS neg	12358	C06313	HMDB00468	445040	1.57	0.0294
3622		Pterin Metabolism	pterin	LC/MS neg	43023	C00715	HMDB00802	73000	1.52	0.5061
3633		Hemoglobin and Porphyrin Metabolism	heme	LC/MS pos late	41754	C00032	HMDB03178	26945	0.89	0.6220
3637			biliverdin	LC/MS pos late	2137	C00500	HMDB01008	5353439	0.99	0.9388
3646		Thiamine Metabolism	thiamin (Vitamin B1)	LC/MS pos early	5341	C00378	HMDB00235	1130	2.17	0.0215
3647			thiamin monophosphate	LC/MS neg	15798	C01081	HMDB02666	3382778	1.50	0.1940
3654		Vitamin A Metabolism	retinol (Vitamin A)	LC/MS pos late	1806	C00473	HMDB00305	445354	1.18	0.6031
3671		Vitamin B6 Metabolism	pyridoxamine phosphate	LC/MS pos early	3138	C00647	HMDB01555	1053	1.27	0.2538
3672			pyridoxal phosphate	LC/MS neg	5331	C00018	HMDB01491	1051	1.06	0.6676
3674			pyridoxate	LC/MS neg	31555	C00847	HMDB00017	6723	2.15	0.0708
3676			hippurate	LC/MS neg	15753	C01586	HMDB00714	464	2.28	0.0165
3680			2-hydroxyhippurate (salicylurate)	LC/MS polar	18281	C07588	HMDB00840	10253	1.79	0.2466
3682			3-hydroxyhippurate	LC/MS neg	39600		HMDB06116	450268	3.38	0.1555
3684			4-hydroxyhippurate	LC/MS neg	35527		HMDB13678	151012	2.92	0.0049
3688			4-hydroxymandelate	LC/MS polar	1568	C03198	HMDB00822	328	2.71	0.0272
3690			benzoate	LC/MS neg	15778	C00180	HMDB01870	243	1.22	0.3477

3708			catechol sulfate	LC/MS neg	35320		HMDB59724	3083879	3.02	0.0095
3711			guaiacol sulfate	LC/MS neg	46111		HMDB60013	22473	2.97	0.0021
3714		Benzoate Metabolism	4-methylcatechol sulfate	LC/MS neg	46146				2.70	0.0130
3719			methyl-4-hydroxybenzoate	LC/MS neg	34386	D01400	HMDB32572	7456	9.46	0.3393
3723			4-acetylphenol sulfate	LC/MS neg	44620			4684006	3.96	0.0077
3726			4-ethylphenylsulfate	LC/MS neg	36099	C13637			2.70	0.0009
3727			4-vinylphenol sulfate	LC/MS neg	36098	C05627	HMDB04072	6426766	10.93	0.0413
3741			methyl-4-hydroxybenzoate sulfate	LC/MS neg	48429				2.03	0.0239
3748			p-cresol sulfate	LC/MS neg	36103		HMDB11635	4615423	3.47	0.0078
3759			3-(3-hydroxyphenyl)propionate sulfate	LC/MS neg	45415			187488	2.01	0.1430
3762			3-(3-hydroxyphenyl)propionate	LC/MS neg	35635	C11457	HMDB00375	91	0.92	0.8006
3764			3-phenylpropionate (hydrocinnamate)	LC/MS neg	15749	C05629	HMDB00764	107	1.18	0.6958
3847			genistein	LC/MS neg	32448	C06563	HMDB03217	5280961	0.94	0.9155
3848			genistein glucuronide*	LC/MS neg	62063				5.35	0.0706
3885			2,3-dihydroxyisovalerate	LC/MS polar	38276	C04039	HMDB12141	677	2.36	0.0614
3889			2,8-quinolinediol sulfate	LC/MS neg	48691				2.73	0.0824
3912			betonicine	LC/MS pos early	38100	C08269	HMDB29412	164642	2.95	0.0000
3916			gluconate	LC/MS polar	587	C00257	HMDB00625	10690	1.20	0.1434
3936			beta-guanidinopropanoate	LC/MS pos early	35101	C03065	HMDB13222	67701	0.96	0.8043
3958			cinnamoylglycine	LC/MS neg	38637		HMDB11621	709625	3.06	0.0038
3975			daidzein	LC/MS neg	32453	C10208	HMDB03312	5281708	1.75	0.2300
3976			daidzein glucuronide*	LC/MS neg	62070		HMDB41718	11316354	5.04	0.0919
3988	Xenobiotics		equol	LC/MS neg	36774	C14131	HMDB02209	91469	1.58	0.4838
3989			equol glucuronide	LC/MS neg	41948				4.27	0.0548
3990		Food Component/Plant	equol sulfate	LC/MS neg	40478				1.24	0.5418
3991			ergothioneine	LC/MS pos early	37459	C05570	HMDB03045	3032311	1.20	0.2756
3996			ferulic acid 4-sulfate	LC/MS polar	47114		HMDB29200	6305574	2.99	0.0594
4018			homostachydrine*	LC/MS pos early	33009	C08283	HMDB33433	441447	2.07	0.0000
4021			indoleacrylate	LC/MS neg	22114		HMDB00734	15030923	1.18	0.5317
4047			N-(2-furoyl)glycine	LC/MS neg	31536		HMDB00439	21863	2.25	0.0848
4053			N-glycolylneuraminate	LC/MS polar	37123	C03410	HMDB00833	123802	1.34	0.3260
4105			quinate	LC/MS polar	18335	C00296	HMDB03072	6508	2.60	0.0004
4130			stachydrine	LC/MS pos early	34384	C10172	HMDB04827	115244	3.14	0.0000
4155			methyl glucopyranoside (alpha + beta)	LC/MS neg	46144				3.11	0.0001
4183			pyrraline	LC/MS neg	48428		HMDB33143	122228	4.19	0.0009
4186			daidzein sulfate (2)	LC/MS neg	48728				1.55	0.2534
4190			N-acetylpyrraline	LC/MS neg	48438				2.07	0.1191
4214			4-ethylphenol glucuronide	LC/MS neg	52937				2.69	0.0056
4271		Bacterial/Fungal	tartronate (hydroxymalonate)	LC/MS neg	20693	C02287	HMDB35227	45	1.09	0.5237
4304			N-methylpipecolate	LC/MS pos early	47101			11286529	4.10	0.0021
4658		Drug - Topical Agents	salicylate	LC/MS polar	1515	C00805	HMDB01895	338	2.30	0.0086
4733			sulfate*	LC/MS neg	46960	C00059	HMDB01448	1118	2.52	0.0257
4736			O-sulfo-L-tyrosine	LC/MS neg	45413			514186	2.71	0.0165
4754			2-aminophenol sulfate	LC/MS neg	43266		HMDB61116	181670	2.77	0.0033
4790			bicine	LC/MS pos early	20708		HMDB11727	8761	1.46	0.5355
4798			dimethyl sulfone	LC/MS pos early	43424	C11142	HMDB04983	6213	4.18	0.1131
4800		Chemical	ectoine	LC/MS polar	35651	C06231		126041	#DIV/0!	0.4980
4831			perfluorooctanesulfonate (PFOS)	LC/MS neg	57564	C18142	HMDB59586	74483	2.38	0.0033
4855			1,2,3-benzenetriol sulfate (2)	LC/MS neg	48762				1.42	0.8500
4858			3-hydroxypyridine sulfate	LC/MS neg	48448				3.37	0.0302
4868			6-hydroxyindole sulfate	LC/MS neg	48698				4.45	0.3798
4872			thioproline	LC/MS pos early	53231			93176	1.20	0.5516
